# Acid/Base‐Responsive Circularly Polarized Luminescence Emitters with Configurationally Stable Nitrogen Stereogenic Centers

**DOI:** 10.1002/adma.202417326

**Published:** 2025-05-15

**Authors:** Pablo García‐Cerezo, Marcos D. Codesal, Arthur H. G. David, Laura Le Bras, Seifallah Abid, Xuesong Li, Delia Miguel, Masoud Kazem‐Rostami, Benoît Champagne, Araceli G. Campaña, J. Fraser Stoddart, Victor Blanco

**Affiliations:** ^1^ Departamento de Química Orgánica Facultad de Ciencias Unidad de Excelencia de Química Aplicada a Biomedicina y Medioambiente (UEQ) Universidad de Granada (UGR) Avda. Fuente Nueva S/N Granada 18071 Spain; ^2^ Department of Chemistry Northwestern University 2145 Sheridan Road Evanston IL 60208 USA; ^3^ Laboratoire MOLTECH‐Anjou (UMR CNRS 6200) Université Angers 2 Bd Lavoisier Angers Cedex 49045 France; ^4^ CNRS Chrono‐environnement (UMR 6249) Université Marie et Louis Pasteur Besançon F‐25000 France; ^5^ Department of Chemistry University of Wyoming Laramie WY 82072 USA; ^6^ Nanoscopy‐UGR Laboratory. Physical Chemistry Department UEQ Faculty of Pharmacy University of Granada C. U. Cartuja Granada 18071 Spain; ^7^ Laboratory of Theoretical Chemistry Namur Institute of Structured Matter (NISM) University of Namur rue de Bruxelles, 61 Namur 5000 Belgium; ^8^ Department of Chemistry The University of Hong Kong Hong Kong, SAR 999077 China; ^9^ Stoddart Institute of Molecular Science Department of Chemistry Zhejiang University Hangzhou 310027 China; ^10^ ZJU‐Hangzhou Global Scientific and Technological Innovation Center Hangzhou Hangzhou 311215 China; ^11^ Center for Regenerative Medicine and Department of Medicine Northwestern University 303 East Superior Street Chicago IL 60611 USA; ^12^ School of Chemistry University of New South Wales Sydney NSW 2052 Australia

**Keywords:** chiroptical switch, circularly polarized luminescence, configurationally stable nitrogens, fluorophores, Tröger's Base

## Abstract

A way to prevent the fast configurational interconversion of tertiary amines is to invoke Tröger's base analogs, which display methano‐ or ethano‐bridged diazocine cores fused to aromatic rings. These derivatives are configurationally stable, even in acidic media when their structures bear ethylene bridges. Here, a two‐ to three‐step synthesis is presented of methano‐ and ethano‐bridged Tröger's base analogs with two peripheral fluorophores, i.e., anthracene, pyrene, and 9,9‐dimethylfluorene units. These compounds, possessing two nitrogen stereogenic centers, exhibit good circularly polarized luminescence (CPL) dissymmetry factors (|*g*_lum_| up to 1.2 × 10^−3^) and brightnesses (*B*_CPL_ up to 26.3 M^−1^ cm^−1^), as well as excellent fluorescence quantum yields, demonstrating the Tröger´s base core to be a convenient scaffold to prepare CPL emitters upon functionalization with simple achiral fluorophores. Furthermore, the configurationally stable ethano‐bridged Tröger's base analogs are employed to modulate their CPL response, generating a CPL switch through their protonation/deprotonation by consecutive additions of acid and base. The reversibility of the switching process is demonstrated for two cycles without altering the CPL performance of the molecule. It is believed that this straightforward and efficient approach to building CPL emitters employing the Tröger's base core could lead to its incorporation in CPL‐based sensors and materials.

## Graphical abstract

Circularly Polarized Luminescence (CPL) emitters based on Tröger's base analogues bearing two peripheral fluorophores (anthracene, pyrene or 9,9‐dimethylfluorene) are prepared following a straightforward and versatile synthetic approach from common precursors. The use of configurationally stable ethano‐bridged Tröger's base analogs leads to an acid/base‐driven CPL switch.

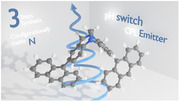

## 1 Introduction

Chirality is of the utmost importance in nature and life since a plethora of naturally occurring organic compounds include stereogenic centers. The vast majority of point chiral stereogenic centers are, however, limited to carbon atoms, although the bond geometry of other atoms, such as nitrogen, can also give rise to chirality. The difficulty in obtaining configurationally stable point chiral nitrogen atoms resides in the fact the lone pair of nitrogen atoms is inverting^[^ ^1^ ^]^ through quantum tunneling, resulting in the fast configurational inversion of tertiary amines carrying three different substituents. This racemization process can be hampered by the preparation^[^ ^2^ ^]^ of ammonium cations, which are widely used in medicine^[^ ^3^ ^]^ and asymmetric catalysis,^[^ ^4^ ^]^ *N*‐oxides^[^ ^5^ ^]^ or chloro‐aziridines.^[^ ^6^ ^]^ Other alternatives are Tröger's base analogs,^[^ ^7^ ^]^ discovered in 1887 by Julius Tröger.^[^ ^8^ ^]^ Their structures, which were elucidated^[^ ^9^ ^]^ and confirmed^[^ ^10^ ^]^ by X‐ray crystallography decades later, consist of bicyclic compounds with central methanodiazocine cores and two peripheral aromatic rings. The chiral resolution of these V‐shaped compounds, possessing *C*_2_ symmetry and two point chiral nitrogen atoms, was performed in 1944 by Prelog and Wieland.^[^ ^11^ ^]^ The bicyclic structure of the Tröger's base core locks the configuration at the chiral N centers, precluding its inversion, which is possible only if a covalent bond between the methylene group and one of the N atoms is broken. Nonetheless, methano‐bridged Tröger's base analogues racemize in acidic media.^[^ ^11^, ^12^ ^]^ The classical proposed mechanism postulates the formation of a methylene‐iminium cation, breaking the methano‐bridge, and allowing for the configurational inversion of the nitrogen atoms,^[^ ^12^, ^13^ ^]^ with a reported enantiomerization barrier of ca. 100 kJ mol^−1^.^[^ ^12c^ ^]^ An alternative mechanism based on a retro‐hetero Diels‐Alder ring opening, followed by a subsequent hetero Diels‐Alder closing has also been proposed in the gas phase.^[^ ^12a^ ^]^ The presence of an ethylene bridge on Tröger's base analogs, however, renders this racemization process under acidic conditions impossible and, hence, affords configurationally stable nitrogen stereogenic centers.^[^ ^14^ ^]^ Tröger's base analogs, which have found a myriad of applications in organic electronics and photonics,^[^ ^15^ ^]^ biotechnology^[^ ^16^ ^]^ and sensing,^[^ ^17^ ^]^ have also been incorporated as creative building blocks into innovative materials, e.g., polymers^[^ ^18^ ^]^ and metal‐organic frameworks.^[^ ^19^ ^]^

Circularly polarized luminescence (CPL) is a property of non‐racemic chiral luminescent species describing their preferential emission of left‐ or right‐handed circularly polarized light.^[^ ^20^ ^]^ The investigation on this chiroptical property is rapidly accelerating^[^ ^21^ ^]^ as CPL acquisition technology becomes more widely available, leading to the emergence of novel applications ranging from chiroptical security inks,^[^ ^22^ ^]^ and sensing,^[^ ^23^ ^]^ to circularly polarized organic light‐emitting diodes (CP‐OLEDs)^[^ ^24^ ^]^ and lasers.^[^ ^25^ ^]^ Among CPL‐emissive organic molecules,^[^ ^26^ ^]^ the majority of those with covalent point chirality possess asymmetric carbon atoms.^[^ ^21^, ^27^ ^]^ The investigation of CPL‐emissive compounds with point chiral nitrogen stereogenic centers remains underexplored, with just a few examples reported.^[^ ^28^, ^29^ ^]^ Thus, Jiang, Wang, and co‐workers^[^ ^28^ ^]^ have built a CPL‐emissive methano‐bridged Troger's base analog while Yoshigoe and co‐workers^[^ ^29^ ^]^ have recently developed a methano‐bridged Troger's base‐containing cycloparaphenylene (CPP). The chiral nitrogen atoms in the system prepared by Jiang and Wang,^[^ ^28^ ^]^ however, are configurationally unstable under acidic conditions on account of the presence of the methylene bridge. Although the CPP‐methano‐bridged Troger's base hybrid reported by Yoshigoe^[^ ^29^ ^]^ is configurationally stable on account of the rigidity of the cyclophane prohibiting the chiral inversion process under acidic conditions, its synthesis requires numerous demanding steps. The synthesis of simpler CPL emitters with configurationally stable chiral nitrogen atoms is yet to be explored. In this context, the construction of ethano‐bridged Tröger's base analogs with configurationally stable nitrogen stereogenic centers, capable of emitting circularly polarized light, is fundamentally relevant and could be of key importance in the future development of smart photonic materials, sensors, and devices.

**Figure 1:**
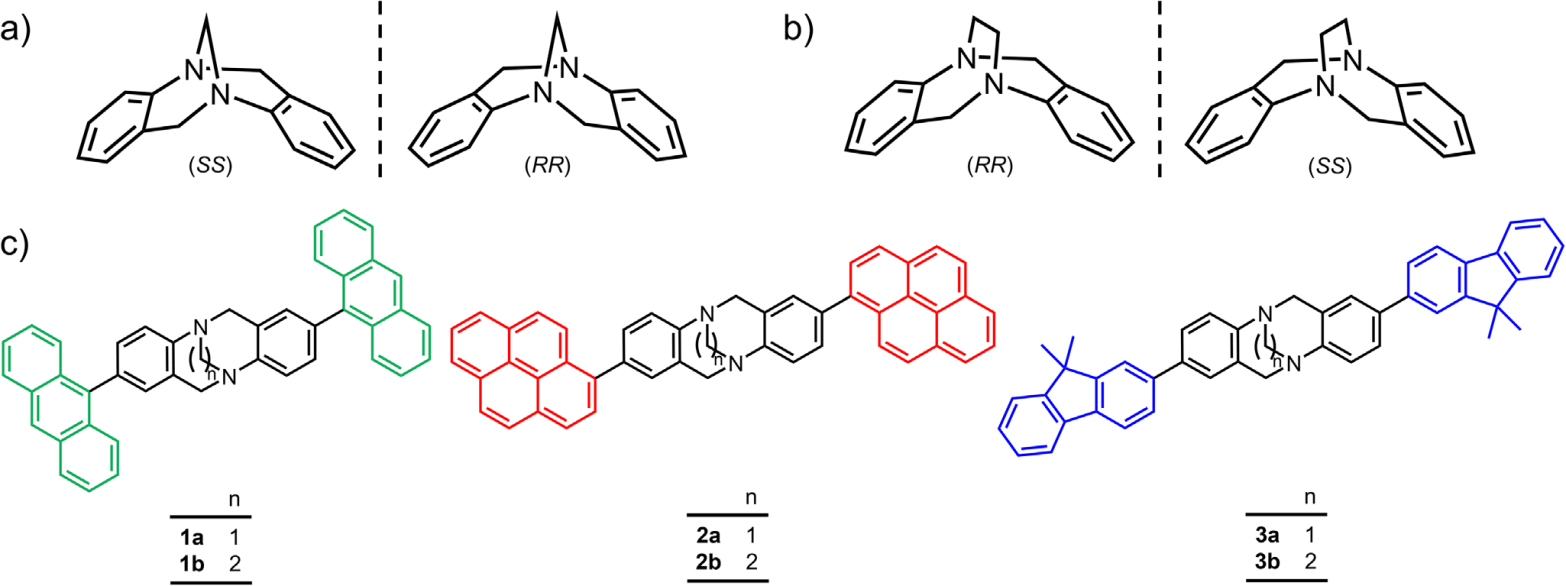
Structural formulae of a) (*RR*) and (*SS*) methano‐ and b) ethano‐bridged Tröger's base cores displaying the enantiomers and the point chirality of the nitrogen atoms. c) Structural formulae of the photoluminescent point‐chiral methano‐ and ethano‐bridged Tröger's base analogs **1a,b‐3a,b** investigated in this research, bearing two emissive anthracene, pyrene, or 9,9‐dimethylfluorene units. The Tröger's base analogs **1b‐3b** possess configurationally stable stereogenic nitrogen centers in acid media on account of the ethano bridge.

Herein, we report the efficient and straightforward three‐step syntheses of Tröger's base analogs bearing two peripheral fluorophores namely, anthracene, pyrene or 9,9‐dimethylfluorene units while displaying point chirality from configurationally stable nitrogen stereogenic centers (**Figure** **1**). UV–vis absorption and emission spectroscopies have allowed us to visualize the optical properties of the different structures. Moreover, the chiral resolution of the enantiomers by chiral stationary phase high performance liquid chromatography (CSP‐HPLC) also led to their study by electronic circular dichroism (ECD) and CPL, demonstrating that the chiroptical properties of the appended fluorophores are induced by the nitrogen‐based chiral core. Following this approach, it is possible to confer a CPL response (|*g*_lum_| up to 1.2 × 10^−3^ and CPL brightnesses (*B*_CPL_) up to 26.3 M^−1^ cm^−1^) to otherwise achiral fluorophores which is in line with other standard organic CPL emitters. Remarkably, the (chir)optical properties of the ethano‐bridged Tröger's base analogs can be tuned upon the addition of acid in a reversible manner, leading to the design of a CPL switch.

## 2 Results and Discussion

### 2.1 Synthesis

**Scheme 1:**
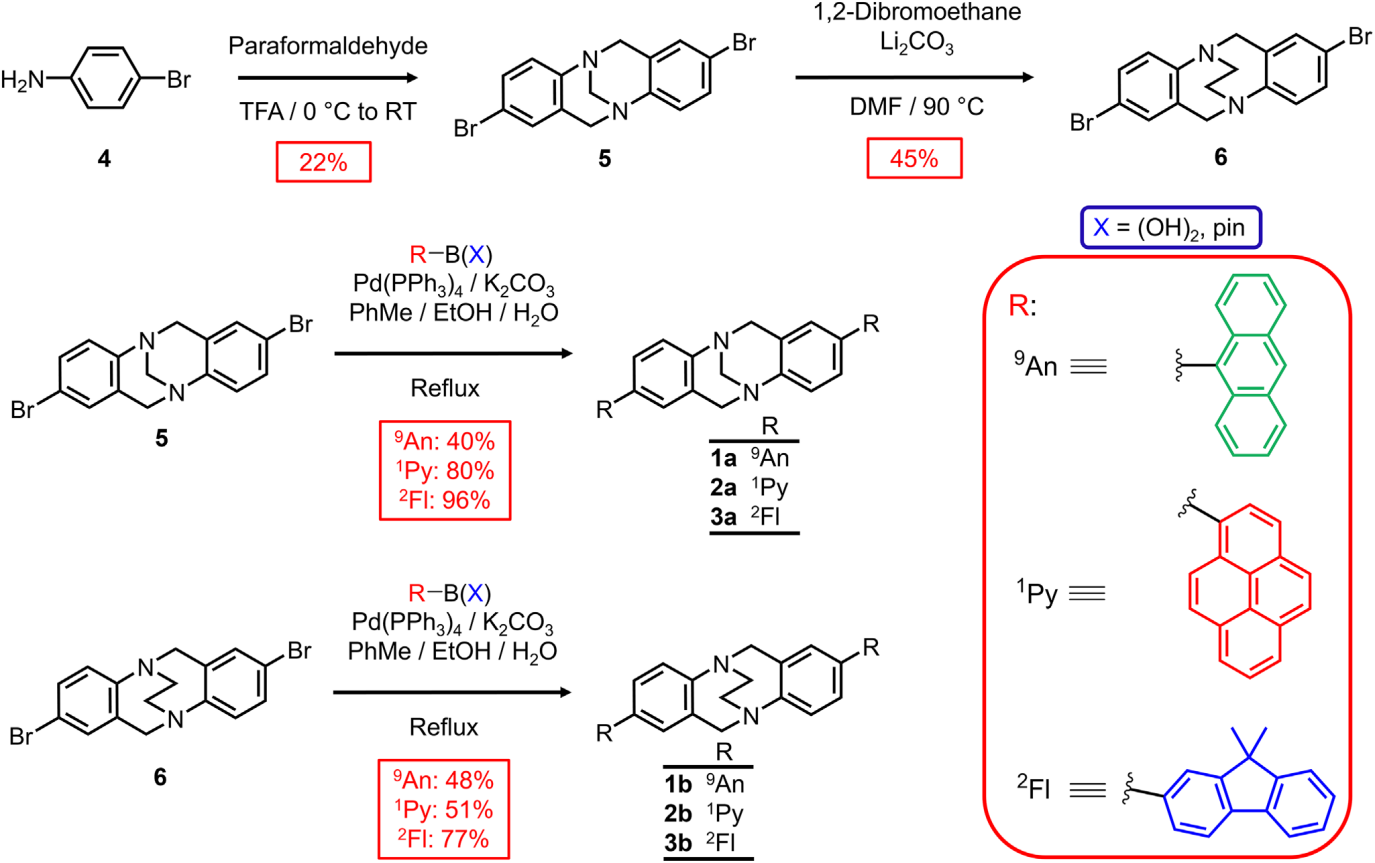
Synthesis of the fluorescent point chiral methano‐ and ethano‐bridged Tröger's base analogs **1a,b‐3a,b**.

The synthetic route (**Scheme** **1**) starts with the formation of the 2,8‐dibromo methano‐bridged Tröger's base analog^[^ ^30^ ^]^ **5** as a result of a condensation reaction between 4‐bromoaniline (**4**) and paraformaldehyde under acidic conditions in 22% yield. Treatment of **5** with 1,2‐dibromoethane in the presence of Li_2_CO_3_ affords 2,8‐dibromo ethano‐bridged Tröger's base analog^[^ ^31^ ^]^ **6** in 45% yield. Suzuki–Miyaura coupling^[^ ^32^ ^]^ between 2,8‐dibromo‐functionalized intermediates **5** or **6**, and the aryl boronic acid or esters of the corresponding fluorophores gave the desired methano‐ and ethano‐bridged Tröger's base analogs **1a,b‐3a,b** bearing two peripheral luminescent moieties—namely anthracene, pyrene or 9,9‐dimethylfluorene units—in moderate to excellent yields (40−96%). These compounds were characterized by NMR spectroscopy and high‐resolution mass spectrometry (HRMS) (See Supporting Information for details). Noteworthy is the fact that intermediates **5** and **6** offer the opportunity to graft a variety of fluorophores by means of cross‐coupling reactions. This synthetic strategy toward the production of chiral compounds with appended fluorophores, and therefore, potential CPL emission, is straightforward and only requires two to three steps, a situation which is rather uncommon for CPL emitters. This approach has a potential for great versatility as, in principle, it could lead to a variety of emissive chiral compounds with CPL activity, by attaching different fluorophores to the Tröger's base core.

### 2.2 Optical Properties

**Figure 2:**
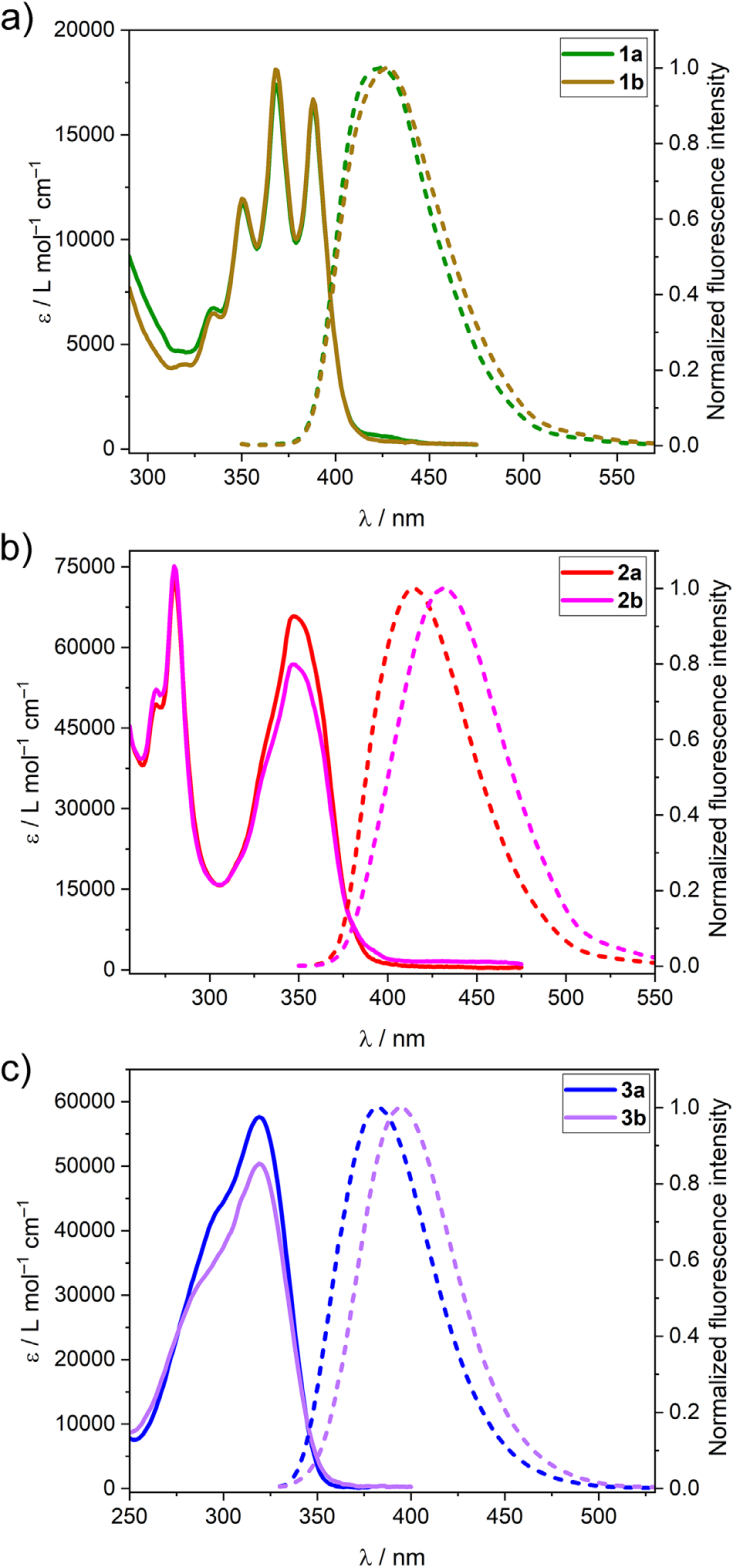
UV–vis absorption (solid lines) and normalized emission (dashed lines) spectra of methano‐ and ethano‐bridged Tröger's base analogs a) **1a,b** (λ_exc_ = 340 nm), b) **2a,b** (λ_exc_ = 340 nm), and c) **3a,b** (λ_exc_ = 320 nm) recorded at r.t. in CH_2_Cl_2_ at concentrations of ca. 6 × 10^−5^ m (for **1a,b**) and 2 × 10^−5^ m (for **2a,b‐3a,b**).

The optical properties of the racemic mixtures of anthracene‐, pyrene‐ and 9,9‐dimethylfluorene‐functionalized Tröger's base analogs **1a,b‐3a,b** were investigated by steady‐state UV–vis absorption and fluorescence spectroscopies. Both UV–vis absorption spectra (**Figure** **2**a, solid lines) of anthracene‐functionalized Tröger's base analogs **1a** and **1b** are very similar. They exhibit a structured absorption band between 310 and 420 nm with three peaks whose maxima are to be found at 350, 368 (ɛ = 1.7 × 10^4^ M^−1^ cm^−1^ for **1a**, and ɛ = 1.8 × 10^4^ M^−1^ cm^−1^ for **1b**), and 388 nm. The fluorescence spectra (Figure 2a, dashed lines) of **1a** and **1b** are also similar and show an emission band between 380 and 510 nm centered at 424 for **1a** (Φ_F_ = 48%, τ = 4.77 ns) and 427 nm for **1b** (Φ_F_ = 54%, τ = 5.44 ns). These optical properties can be attributed to the 9‐phenylanthracene units,^[^ ^33^ ^]^ since the methano‐ or ethano‐bridged Tröger's base cores do not show any significant absorbance above 310 nm. Regarding pyrene‐functionalized Tröger's base analogs **2a** and **2b**, their UV–vis absorption spectra (Figure 2b) display similar shapes portrayed by two absorption bands, one between 250 and 300 nm centered on 280 nm, and the other between 300 and 380 nm centered on 347 nm. Their molar extinction coefficients are, however, different—ɛ_347_ = 6.6 × 10^4^ M^−1^ cm^−1^ for **2a**, and ɛ_347_ = 5.7 × 10^4^ M^−1^ cm^−1^ for **2b**. The fluorescence spectra (Figure 2b) of both **2a** and **2b** display an emission band between 370 and 525 nm. Nonetheless, the fluorescence maximum of **2b** (λ_em_ = 431 nm, Φ_F_ = 61%, τ = 3.05 ns) is red‐shifted compared to that (λ_em_ = 415 nm, Φ_F_ = 66%, τ = 4.27 ns) of **2a**. The photoluminescence of both compounds **2a** and **2b** is characteristic of the peripheral 2‐phenylpyrene units.^[^ ^34^ ^]^ Finally, 9,9‐dimethylfluorene‐based Tröger's base analogues **3a** and **3b** show similar UV–vis absorption spectra (Figure 2c) with the main absorption band between 250 and 355 nm, centered on 319 nm. Their molar extinction coefficients, however, are different: ɛ_319_ = 5.8 × 10^4^ M^−1^ cm^−1^ for **3a**, and ɛ_319_ = 5.0 × 10^4^ M^−1^ cm^−1^ for **3b**. In common with the pyrene‐based Tröger's base analogs, both **3a** and **3b** emit (Figure 2c) in the same spectral region, between 330 and 480 nm. The emission maximum of **3b** (λ_em_ = 394 nm, Φ_F_ = 76%, τ = 1.91 ns), however, is red‐shifted compared to that (λ_em_ = 382 nm, Φ_F_ = 76%, τ = 1.28 ns) of **3a**. The fluorescence of **3a** and **3b** can be attributed to the peripheral fluorene units.^[^ ^35^ ^]^ It is worth emphasizing that the quantum yields of all Tröger's base analogs are outstanding: they are higher when a 9,9‐dimethylfluorene substituent is incorporated into the structure, reaching a high value of 76%.

### 2.3 Chiroptical Properties

Chiral resolution of Tröger's base analogues **1a,b‐3a,b** was achieved by using CSP‐HPLC.^[^ ^36^ ^]^ The reader is directed to the Supporting Information for the detailed chiral resolution procedures. It is worth noting that, although in this work we use CSP‐HPLC for the enantiomeric resolution, the enantiomers of the Tröger base derivatives could be also accessible by diastereomeric resolution by co‐crystallization of the dibromo precursors with (2*R*,3*R*)‐ or (2*S*,3*S*)‐*O,O’*‐dibenzoyl tartaric acid,^[^ ^37^ ^]^ followed by the corresponding Suzuki coupling. As proof‐of‐concept, we applied this strategy for the preparation of (*RR*)‐**2a** (see Supporting Information for details).

**Figure 3:**
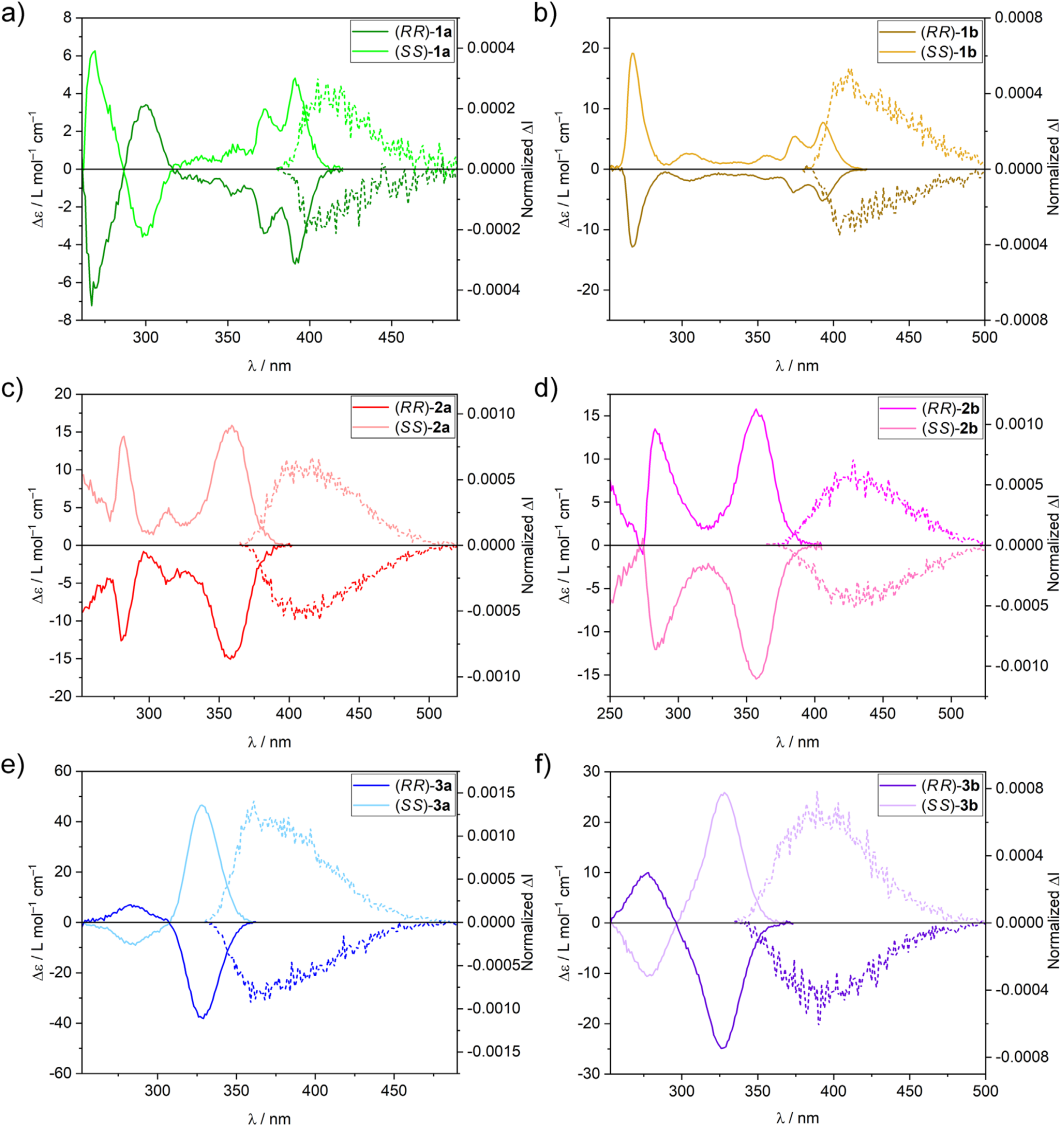
Chiroptical properties of the photoluminescent methano‐ and ethano‐bridged Tröger's base analogs (*RR*)/(*SS*)‐**1a,b‐3a,b**. ECD (solid lines) and CPL (dashed lines) spectra of: a) (*RR*)/(*SS*)‐**1a** (λ_exc_ = 403 nm), b) (*RR*)/(*SS*)‐**1b** (λ_exc_ = 403 nm), c) (*RR*)/(*SS*)‐**2a** (λ_exc_ = 372 nm), d) (*RR*)/(*SS*)‐**2b** (λ_exc_ = 372 nm), e) (*RR*)/(*SS*)‐**3a** (λ_exc_ = 340 nm), and f) (*RR*)/(*SS*)‐**3b** (λ_exc_ = 342 nm) recorded at r.t. in CH_2_Cl_2_ at concentrations of ca. 6 × 10^−5^ m (for **1a,b**) and 2 × 10^−5^ m (for **2a,b‐3a,b**).

Next, the chiroptical properties of these enantiopure fluorophore‐functionalized Tröger's base analogs (*RR*)/(*SS*)‐**1a,b‐3a,b** were investigated by a combination of ECD and CPL spectroscopies. **Table** **1** summarizes the main optical and chiroptical parameters for the compounds. ECD Spectra (**Figure** **3**a,b, solid lines) of enantiopure anthracene‐functionalized Tröger's base analogs (*RR*)/(*SS*)‐**1a,b** exhibit several bands within the range 250−415 nm with alternated Cotton effects for **1a** and one sign of dichroism value for **1b**. The spectra of both compounds are mirror images, as expected for pairs of enantiomers. With respect to the lowest energy transition, (*SS*)‐**1a** and (*SS*)‐**1b** show positive Cotton effects at 391 nm (|Δɛ| ≈ 5 M^−1^ cm^−1^, |*g*_abs_| = |Δɛ|/ɛ ≈ 4 × 10^−4^), and 393 nm (|Δɛ| ≈ 8 M^−1^ cm^−1^, |*g*_abs_| ≈ 7 × 10^−4^), respectively. For both enantiopure pyrene‐functionalized Tröger's base analogs (*RR*)/(*SS*)‐**2a,b**, their ECD spectra (Figure 3c,d) show a similar shape composed of several bands in the range 250−395 nm with only one sign of dichroism value. Those spectra are ideal mirror images. With respect to the lowest energy transition, both (*SS*)‐**2a** and (*RR*)‐**2b** show very similar behavior since they feature positive Cotton effects at 359 nm (|Δɛ| ≈ 15 M^−1^ cm^−1^, |*g*_abs_| ≈ 3 × 10^−4^), and 357 nm (|Δɛ| ≈ 16 M^−1^ cm^−1^, |*g*_abs_| ≈ 3 × 10^−4^), respectively. Regarding enantiopure 9,9‐dimethylfluorene‐functionalized Tröger's base analogs (*RR*)/(*SS*)‐**3a,b**, their ECD spectra (Figure 3e,f) are mirror images and exhibit again similar shapes consisting of two bands with alternated Cotton effects. Their magnitude is, however, significantly different since, for the lowest energy transition, (*SS*)‐**3a** and (*SS*)‐**3b** display positive Cotton effects at 328 nm (|Δɛ| ≈ 47 M^−1^ cm^−1^, |*g*_abs_| ≈ 9 × 10^−4^), and 328 nm (|Δɛ| ≈ 26 M^−1^ cm^−1^, |*g*_abs_| ≈ 6 × 10^−4^), respectively.

**Table 1:** Summary of the Photophysical Properties of the Chiral Tröger's Base Analogues **1a,b‐3a,b** in CH_2_Cl_2_.

Compound	λ_abs_ / nm	ɛ / M^−1^ cm^−1^	λ_em_ ^a)^ / nm	Φ_F_ / %	τ^b)^ / ns	|*g*_abs_|^c)^	|*g*_lum_|	*B*_CPL_ ^d)^/ M^−1^ cm^−1^
**1a**	368	1.7 × 10^4^	424	48	4.77	4 × 10^−4^	2.5 × 10^−4^	1.02
**1b**	368	1.8 × 10^4^	427	54	5.44	7 × 10^−4^	4.6 × 10^−4^	2.24
**2a**	347	6.6 × 10^4^	415	66	4.27	3 × 10^−4^	5.8 × 10^−4^	12.6
**2b**	347	5.7 × 10^4^	431	61	3.05	3 × 10^−4^	5.3 × 10^−4^	9.21
**3a**	319	5.8 × 10^4^	382	76	1.28	9 × 10^−4^	1.2 × 10^−3^	26.3
**3b**	319	5.0 × 10^4^	394	76	1.91	6 × 10^−4^	6.5 × 10^−4^	12.3

^a)^ Obtained from the fluorescence spectrum of the compounds using the following excitation wavelength: λ_exc_ = 340 nm (for **1a,b**, and **2a,b**); 320 nm (for **3a,b**);

^b)^ Corresponds to τ_1_ or τ_average_, see Table S14 (Supporting Information) for more details;

^c)^ The |*g*_abs_| values given in this Table correspond to the dimensionless dissymmetry factor values at the maximum of the lowest energy transitions;

^d)^ Calculated at λ_exc_ = 368 nm (for **1a,b**); 347 nm (for **2a,b**); 319 nm (for **3a,b**).

**Figure 4:**
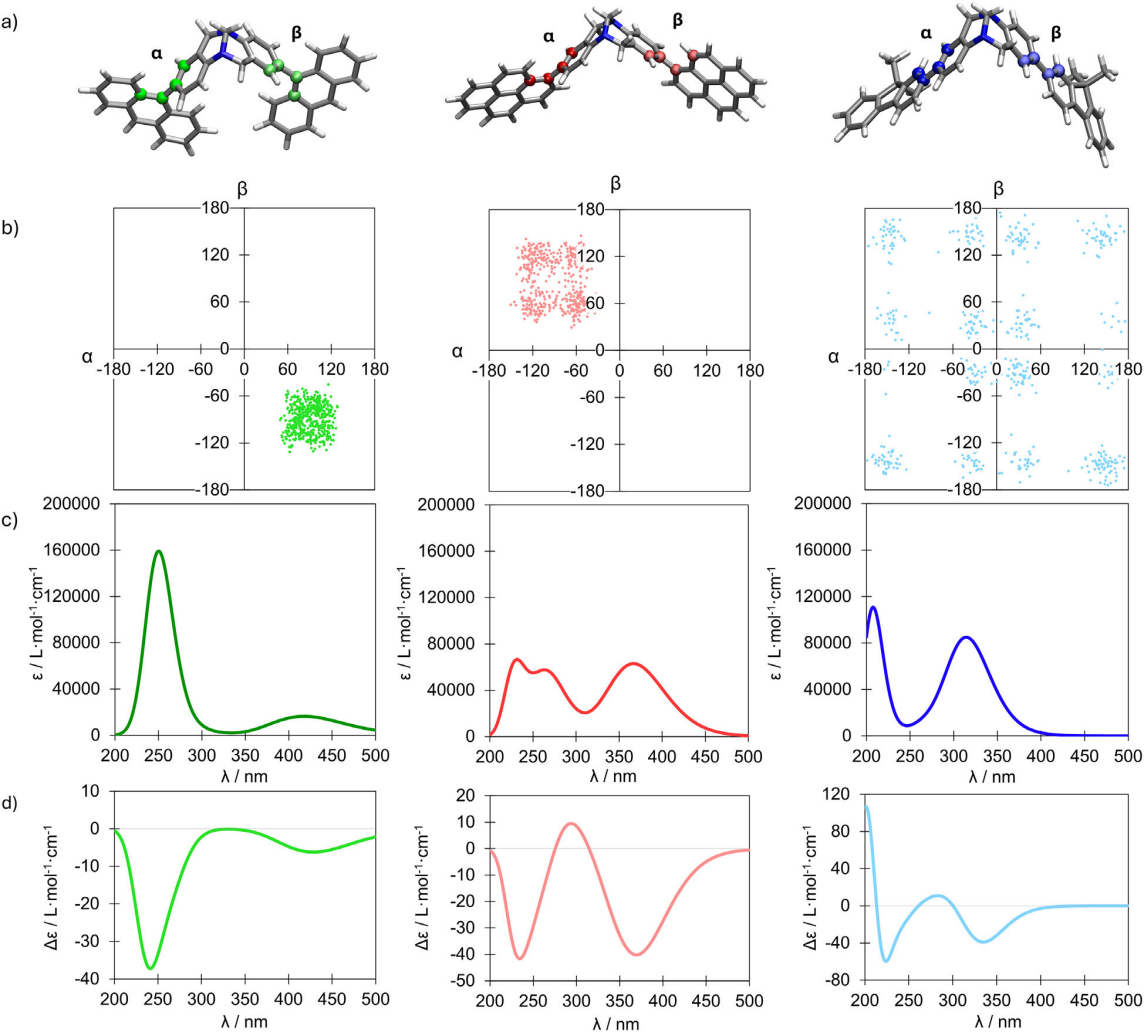
a) Structural formulae of methano‐bridged Tröger's base analogs (*RR*)‐**1a‐3a** showing the two dihedral angles α and β. b) Representation of the dihedral couple (α;β) of (*RR*)‐**1a** (left), (*RR*)‐**2a** (middle), and (*RR*)‐**3a** (right) along the 20 ns MD simulation, where each point represents a snapshot of the simulation. For **1a,b‐2a,b**, because of steric hindrance, only the half‐rotation is possible and the dihedral angles extend over a 180° range (either [0;180] or [−180;0]). Note that for **3a,b**, since the complete rotation of the fluorene moieties is possible, the dihedral angles range from −180 to +180°. Averaged calculated c) UV–vis absorption and d) ECD spectra of (*RR*)‐**1a** (green lines, left), (*RR*)‐**2a** (red lines, middle), and (*RR*)‐**3a** (blue lines, right) for 50 extracted snapshots of MD simulation (full width at half maximum (FWHM) = 0.3 eV). The results for (*SS*)‐**1b‐3b** are presented in the Supporting Information.

The assignment of the configurations of the Tröger's base analogs was performed by comparison of the experimental and theoretical ECD spectra, which were calculated employing a combination of molecular dynamics (MD) and time‐dependent density functional theory (TD‐DFT). See the Supporting Information for the computational details. Initially, MD simulations of (*RR*)‐**1a‐3a** and (*SS*)‐**1b‐3b** were performed, highlighting their structural flexibility on account of the rotational motion of the different lateral fluorophores of the Tröger's base analogs. These conformational changes are defined (**Figure** **4**a; Figures S94 and S95, Supporting Information) by a couple of dihedral angles α and β, corresponding to the angle between the benzene ring of the Tröger's base core and the ring of the fluorophore attached to it. Throughout the 20 ns MD simulation (Figure 4b; Figure S95, Supporting Information), anthracene‐appended Tröger's base analogs **1a,b** exhibit lower flexibilities engendering two main conformations (Table S17, Supporting Information), while pyrene‐appended **2a,b** and 9,9‐dimethylfluorene‐appended **3a,b** show (Tables S18 and S19, Supporting Information) four and 16 main conformations respectively. These main conformations of Tröger's base analogs (*RR*)‐**1a‐3a** and (*SS*)‐**1b‐3b** were optimized at the CAM‐B3LYP/def2tzvp level of theory and their optical and chiroptical properties were computed. The resulting absorption spectra are similar for each conformation and are blue‐shifted (Tables S17–S19, Supporting Information) compared to the experimental ones. The absorption maxima *λ*_abs_, found experimentally (Table 1), can be ascribed to HOMO−1/HOMO to LUMO/LUMO+1 transitions with a n/π → π* character (Figures S96–S98, Supporting Information). The ECD spectra computed for the **1a,b‐3a,b** conformations (Figure S99, Supporting Information) exhibit a low (**1a,b**) or a large (**2a,b‐3a,b**) fluctuation for the same Tröger's base derivative. At this point, the configurational assignment of an enantiomer to a given spectrum is not straightforward since the flexibility of these molecules modifies considerably the resulting ECD properties.

Therefore, in order to increase the accuracy of the computed ECD properties of these compounds,^[^ ^38^ ^]^ UV–vis spectra (Figure 4c; Figure S95c, Supporting Information) and ECD (Figure 4d; Figures S95d and S100, Supporting Information) spectra have been computed and averaged for Tröger's base analogs (*RR*)‐**1a‐3a** and (*SS*)‐**1b‐3b** over 50 snapshots extracted from MD simulations. The computed UV–vis spectra, for all compounds, are in good agreement with the experimental ones despite a redshift of the theoretical spectra. For (*RR*)‐**1a**, the global trend of the experimental spectrum is reproduced with alternating negative (310−500 nm), slightly positive (290−310 nm), and negative peaks (under 280 nm) in the ECD theoretical spectrum. For (*SS*)‐**1b**, the computed ECD spectrum is always positive in the 275−500 nm range (Figure S95, Supporting Information), which is similar to the experimental one. For pyrene‐containing Tröger's base analogs, the configurational assignments are not so easy as a clear alternation of the sign of the peaks is found theoretically while experimentally the spectrum is almost fully negative or positive depending on the enantiomers. Nonetheless, for (*RR*)‐**2a**, the theoretical ECD spectrum reflects two main negative peaks (320−400 and 210−275 nm) which are found experimentally. For (*SS*)‐**2b**, the computed ECD spectrum, however, is essentially negative and displays a positive peak between 275 and 310 nm. By considering carefully the experimental ECD spectrum of (*RR*)/(*SS*)‐**2b**, a brief alternation of the Cotton effect is identified at ca. 273 nm and may be overestimated by the theoretical calculations.^[^ ^39^ ^]^ Finally, for (*RR*)‐**3a** and (*SS*)‐**3b**, the experimental ECD spectrum is reproduced with alternated positive and negative signs for the dichroism value. The combination of MD simulations and TD‐DFT calculations appears to be a suitable way to assign configurations to the enantiomers of these flexible fluorophore‐appended Tröger's base analogs.

CPL Spectra (Figure 3, dashed lines) of the photoluminescent enantiopure methano‐ and ethano‐bridged Tröger's base analogs (*RR*)/(*SS*)‐**1a,b‐3a,b** were recorded upon excitation with UV–vis light. The six enantiomeric pairs of the point chiral nitrogen‐based compounds (*RR*)/(*SS*)‐**1a,b‐3a,b** afford mirror image CPL responses between ca. 380 and 500 nm for the anthracene and pyrene‐functionalized Tröger's base analogs (*RR*)/(*SS*)‐**1a,b‐2a,b**, and between 330 and 475 nm for the 9,9‐dimethylfluorene‐functionalized Tröger's base analogs (*RR*)/(*SS*)‐**3a,b**. These compounds exhibit dissymmetry factors, |*g*_lum_|,^[^ ^40^ ^]^ ranging from 2.5 × 10^−4^ to 1.2 × 10^−3^. Furthermore, the signs of the CPL signals are in line with those obtained by ECD spectroscopy for the lowest energy absorption transition for each compound. Although |*g*_lum_| values give valuable information on the CPL response of non‐racemic chiral fluorophores, the efficiency of CPL emitters cannot be analyzed exclusively in terms of this parameter, since, for example, the emission intensity is also relevant. As a result, another parameter, the CPL brightness^[^ ^41^, ^42^ ^]^ (*B*_CPL_), has been introduced recently to evaluate and compare the performance of CPL‐active compounds. The *B*_CPL_ of compounds **1a,b‐3a,b** ranges from 1.02 to 26.3 M^−1^ cm^−1^. The highest value is obtained for compound **3a**, because of its excellent fluorescence quantum yield and the higher |*g*_lum_| in comparison with the remaining derivatives. In general, the *B*_CPL_ values, especially those from **2a,b‐3a,b**, are similar to those exhibited by a variety of organic compounds, including helicenes, usually considered as standard CPL emitters.^[^ ^42^ ^]^

Interestingly, the comparison of the CPL responses of the compounds studied shows a better performance of the 9,9‐dimetyfluorenyl derivatives, in special the methano‐bridged Tröger's base analog **3a**. In general, the chiroptical response in terms of *g*_lum_ value depends on the magnetic and electric dipole transition moments, |**m**| and |**µ**|, respectively, and the angle between them (*g*_lum_ = 4(|**µ**|·|**m**|·cos *θ*)/(|**µ**|^2^ + |**m**|^2^) ≈ 4|**m**|·cos *θ*/|**µ**|). Thus, the |*g*_lum_| value increases if the magnetic dipole transition moment increases, the electric dipole transition moment decreases or the angle between them gets closer to 0° or 180°. The rotational freedom around the bond between the Tröger's base core and the fluorophore unit hampers a more precise study of the electronic transitions of the compounds by TD‐DFT because of the high variability of the calculated chiroptical properties upon changing the dihedral angle. From the structure of the compounds, however, we can get some insights that can qualitatively explain to some extent the observed results. It was recently demonstrated^[^ ^43^ ^]^ that in helical structures the magnetic dipole transition moment increases with the inner area, and |**m**| also benefits from the electron density being delocalized over the whole structure. Although Tröger's base derivatives are not helical, we can approximate them to being half a turn. In this context, the substitution pattern of the fluorophores, which influence the structures, could also influence the chiroptical response. The substitution of the 9,9‐dimethylfluorene in position 2 results in the whole fluorophore extending the “half‐turn” and increasing the area and, therefore, |**m**|. On the contrary, with the anthracene moiety, the extension of the structure is lower on account of its functionalization in the 9‐position. Inspection of the dihedral angles between the fluorophore and the Tröger core shows that compounds **3a,b** display angles closer to 0° and 180° than derivatives **1a,b‐2a,b** (Figure 4b; Figure S95b, Supporting Information), resulting in more planar structures. This flatter geometry implies a higher electronic delocalization, as can be seen in the calculated molecular orbitals (Figures S97 and S98, Supporting Information). In contrast, for the anthracene derivatives, the dihedral angles closer to 90° result in no significant delocalization being observed, unlike **3a,b**, in which the frontier orbitals are more delocalized over the Tröger core.

The CPL results demonstrate that the functionalization with fluorophores of methano‐ and configurationally stable ethano‐bridged Tröger's base scaffolds is a remarkable and versatile strategy to prepare organic CPL emitters in a straightforward way, with a reduced number of synthetic steps. Thus, the attachment of different fluorophores to this core confers upon them a chiral environment that induces chiroptical responses, in particular, CPL. This strategy could lead to the transformation of a variety of achiral fluorophores into chiral emitters in a synthetically accessible manner.

### 2.4 Acid/Base‐Driven Fluorescence and CPL Switching

**Figure 5:**
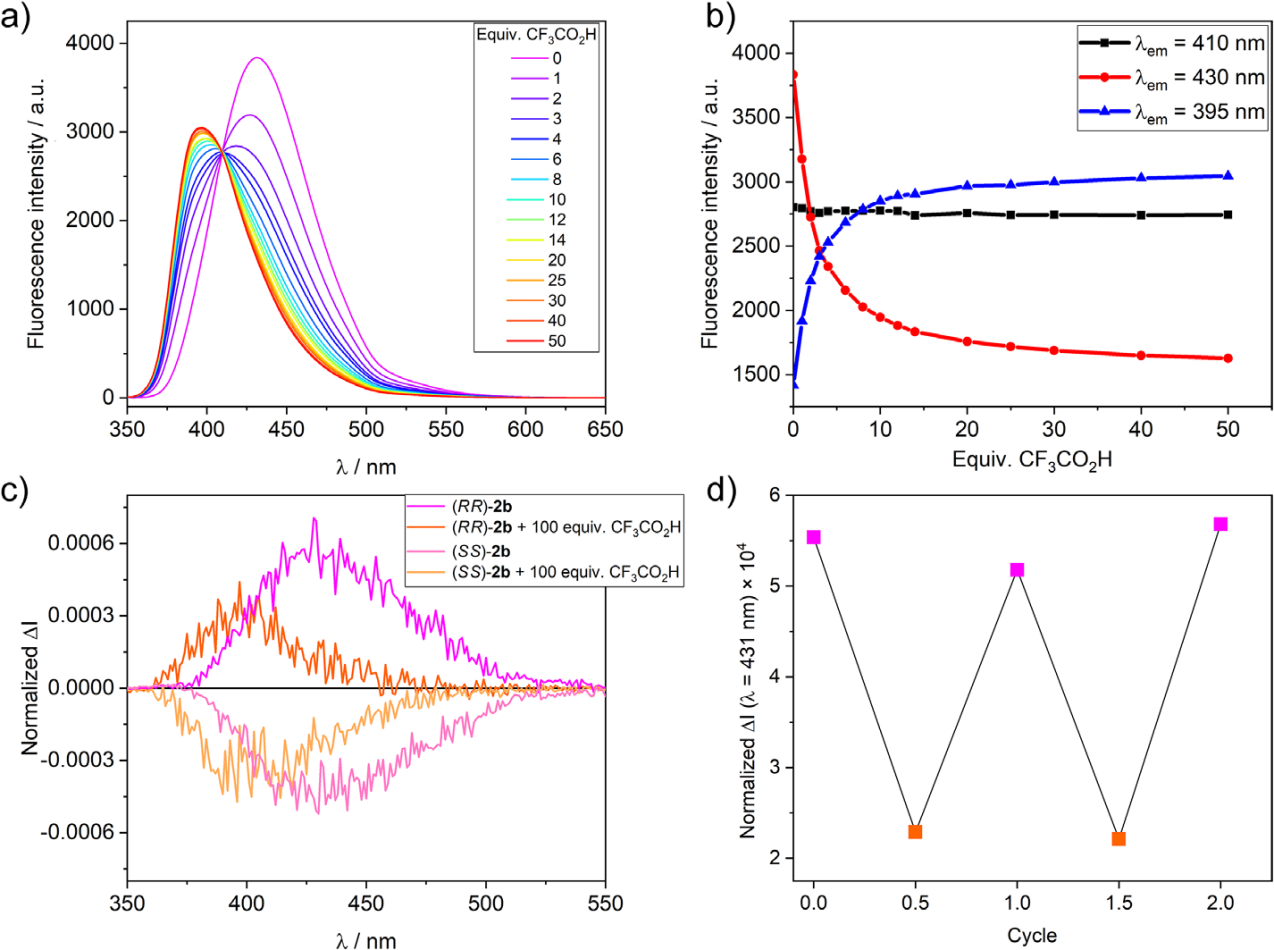
Switching of the fluorescence and CPL signals of ethano‐bridged Tröger's base analog (*RR*)/(*SS*)‐**2b** upon the addition of acid and base. a) Fluorescence emission spectra (λ_exc_ = 340 nm) of **2b** in CH_2_Cl_2_ after the addition of increasing quantities of a CF_3_CO_2_H solution (0 equiv. – 50 equiv.) and b) evolution of the fluorescence intensity at different wavelengths. c) CPL (λ_exc_ = 372 nm) spectra of (*RR*)/(*SS*)‐**2b** in CH_2_Cl_2_ in the presence (orange lines) and the absence (pink lines) of 100 equiv. of CF_3_CO_2_H. d) In situ switching of the CPL signal (λ = 431 nm) of (*RR*)‐**2b** after consecutive addition of CF_3_CO_2_H (cycles 0.5 and 1.5, orange squares) and Et_3_N (cycles 0, 1 and 2, pink squares).

The presence of aniline rings in the structure of compounds **1a,b‐3a,b** could enable the tuning of the optical properties, mainly fluorescence, triggered by protonation or deprotonation of the nitrogen atoms. This methodology is especially appealing in the case of configurationally stable derivatives **1b‐3b**, as it would produce acid/base‐controlled CPL switches.^[^ ^27^, ^44^ ^]^ Hence, we focused on investigating the switching of emission properties of the ethano‐bridged Tröger's base analogs. The addition of increasing amounts of trifluoroacetic acid to **1b‐3b** resulted in a hypsochromic shift of their emission bands, which can be attributed to the loss of the electron‐donor character of the aniline N atom (**Figure** **5**a; Figures S75–S80, Supporting Information).^[^ ^45^ ^]^ The changes in the fluorescence, however, are more pronounced in the case of compounds **2b** (Figure 5a) and **3b** (Figure S79, Supporting Information), with a shift in the λ_em_ from 431 to 397 nm (Δλ_em_ = −34 nm) and from 394 to 365 nm (Δλ_em_ = −29 nm), respectively. This hypsochromic shift of the emission band upon protonation is accompanied by a decrease of the fluorescence intensity, as a result of a decrease of the quantum yield (Φ_F_ = 45% for **2b** and Φ_F_ = 47% for **3b**) after CF_3_CO_2_H addition. In contrast, the Tröger's base analog **1b** undergoes a less marked shift of only 6 nm (from 427 to 421 nm) with a significant increase (Figure S75, Supporting Information) in the fluorescence intensity and fluorescence quantum yield (Φ_F_ = 67%). Clear variations in fluorescence intensity are advisable for the development of CPL switches. In this context, an analysis of the emission profile of **2b** revealed (Figure 5b) certain wavelengths at which a drastic change in the emission intensity is observed. In particular, at 430 nm there is a ca. 50% decrease in the fluorescence intensity upon addition of acid, while at 395 nm the intensity doubles. These changes can be attributed to a decrease in the emission intensity of the signal corresponding to the neutral species (λ_em_ = 431 nm) and the appearance of a new band corresponding to the protonated form (λ_em_ = 395 nm), with a clear isosbestic point at 410 nm. Fluorescence lifetime analyses exhibited (Figure S78, Supporting Information) a different behavior between neutral and cationic species. In contrast to the single fluorescence lifetime observed for **2b** in neutral conditions (τ = 3.05 ns), addition of trifluoroacetic acid results in the presence of three emissive species with τ_1_ = 0.7 ns; τ_2_ = 3.0 ns and τ_3_ = 14.5 ns. The contribution of the species with the shortest lifetime (τ_1_ = 0.7 ns) is almost negligible. The intensity profile of the species with the lifetime of 3.0 ns is in accordance with the emission of the neutral species. The emission with the longer lifetime, however, is shifted to shorter wavelengths, matching the new emission band attributed (Figure S78, Supporting Information) to the protonated species. Remarkably, this fluorescence shifting is reversible. Addition of Et_3_N restores the position of the emission band and its intensity to almost its original value (only a 3% intensity decrease was observed) (Figure S87, Supporting Information). A second cycle of CF_3_CO_2_H and Et_3_N additions was performed (Figure S87, Supporting Information), demonstrating the switching reproducibility. For **1b**, an increase in fluorescence intensity at ca. 422 nm is observed, although the variation is less intense (ca. 16%), together with a very small increase of the fluorescence lifetime (τ_1_ = 6.07 ns). The ethano‐bridged Tröger's base analog **3b** also shows a ca. 70% quenching of the fluorescence at 398 nm upon addition of trifluoroacetic acid despite the fact that no significant change of the lifetime was observed (τ_av_ = 1.26 ns).

From the fluorescence switching studies, the configurationally stable Tröger's base analog **2b** was identified as the most promising candidate for CPL switching studies ^[^ ^46^ ^]^ on account of its clear changes in the fluorescence profile. Hence, addition of an excess of CF_3_CO_2_H promotes a hypsochromic shift of the CPL emission band (Figure 5c), which appears between 360 and 480 nm. This CPL band matches the emission profile of the species with the longest fluorescence lifetime (τ_3_ = 14.5 ns) (Figure S78, Supporting Information), which is, therefore, responsible of the CPL emission. This wavelength shift is accompanied by a decrease of the |*g*_lum_| value to ca. 3 × 10^−4^. These changes are reversed upon the addition of Et_3_N (see Figure 5d; Figures S89–S90, Supporting Information), demonstrating that the CPL modulation is triggered by the addition of acid/base as stimuli. Two consecutive acid‐base cycles were performed with no significant differences between cycles. The changes in the CPL emission can be monitored at 431 nm, where the normalized ΔI varies from ca. 5.3 × 10^−4^ and ca. 2 × 10^−4^ with the addition of acid, while the addition of base restores the original value. Therefore, ethano‐bridged Tröger's base analogs, not only makes it possible to confer CPL emission to simple polycyclic aromatic hydrocarbon‐based achiral fluorophores by attaching them to the chiral core, but also enable the modulation of the CPL by acid/base addition on account of the configurationally stable nature of the N atoms in acid media.

## 3 Conclusion

In summary, we have presented a straightforward and efficient syntheses of CPL emitters based on simple achiral fluorophores attached to methano‐ or ethano‐bridged Tröger's base cores with configurationally stable nitrogen stereogenic centers. As a proof‐of‐concept, we have developed six analogs functionalized with pyrene, anthracene, or 9,9′‐dimethylfluorene moieties. On account of the high fluorescence quantum yields and the good emission dissymmetry factors (up to 1.2 × 10^−3^) of these fluorophore‐appended Tröger's base analogs, the values obtained for their CPL brightnesses (up to 26.3 M^−1^ cm^−1^) are similar to those exhibited by common small organic molecules, especially helicenes,^[^ ^42^ ^]^ frequently viewed as promising organic CPL emitters. The strength of this approach lies in the fact that it yields CPL emitters in only three synthetic steps, while its modular nature opens up the way to a large number of simple achiral luminophores that could potentially be attached to the Tröger's base to yield CPL‐active compounds. For instance, a potential possibility could be the functionalization of the Tröger's base core with multi‐resonance thermally activated delayed fluorescence emitters for the development of suitable materials to build CP‐OLEDs. In addition, the configurationally stable nitrogen stereogenic centers in the ethano‐bridged derivatives offer the possibility to modulate the chiroptical responses in the presence of acid or base. The switching of the CPL signal of the pyrene‐appended ethano‐bridged Tröger's base analog is demonstrated for two consecutive acid‐base addition cycles without significantly altering the chiroptical response. The straightforward syntheses of these fluorophore‐appended configurationally stable Tröger's base analogs provides us with the incentive to apply their remarkable CPL properties in sensing and organic electronic applications. We also believe that compounds based on the Tröger's base core might be of considerable interest in the field of spintronics.^[^ ^47^ ^]^

## Conflict of Interest

The authors declare no conflict of interest.

## Supporting information



Supporting Information

## Data Availability

The data that support the findings of this study are available from the corresponding author upon reasonable request.

## References

[1] J.‐M. Lehn , in Dynamic Stereochemistry, Vol. 15, (Eds.: J. E. Baldwin , R. H. Fleming , J. M. Lehn , W. Tochtermann ), Springer‐Verlag, Berlin 1970, pp. 311–377.

[2] a) E. Fröhlich , E. Wedekind , Chem. Ber. 1907, 40, 1009;

[3] a) N. J. Gross , N. Engl. J. Med. 1988, 319, 486; 2970009 10.1056/NEJM198808253190806

[4] a) U.‐H. Dolling , P. Davis , E. J. J. Grabowski , J. Am. Chem. Soc. 1984, 106, 446;

[5] a) J. F. Traverse , Y. Zhao , A. H. Hoveyda , M. L. Snapper , Org. Lett. 2005, 7, 3151; 16018608 10.1021/ol050814q

[6] a) A. Forni , I. Moretti , A. V. Prosyanik , G. Torre , J. Chem. Soc., Chem. Commun. 1981, 588;

[7] a) B. Dolenský , J. Elguero , V. Král , C. Pardo , M. Valík , Adv. Heterocycl. Chem. 2007, 93, 1;

[8] J. Tröger , J. Prakt. Chem. 1887, 36, 225.

[9] M. A. Spielman , J. Am. Chem. Soc. 1935, 57, 583.

[10] S. B. Larson , C. S. Wilcox , Acta Crystallogr., Sect. C: Cryst. Struct. Commun. 1986, 42, 224.

[11] V. Prelog , P. Wieland , Helv. Chim. Acta 1944, 27, 1127.

[12] a) O. Trapp , V. Schurig , J. Am. Chem. Soc. 2000, 122, 1424;

[13] A. Greenberg , N. Molinaro , M. Lang , J. Org. Chem. 1984, 49, 1127.

[14] a) Y. Hamada , S. Mukai , Tetrahedron Asymmetry 1996, 7, 2671;

[15] a) I. Neogi , S. Jhulki , A. Ghosh , T. J. Chow , J. N. Moorthy , ACS Appl. Mater. Interfaces. 2015, 7, 3298; 25585169 10.1021/am508004n

[16] a) E. B. Veale , D. O. Frimannsson , M. Lawler , T. Gunnlaugsson , Org. Lett. 2009, 11, 4040; 19681640 10.1021/ol9013602

[17] a) E. M. Boyle , S. Comby , J. K. Molloy , T. Gunnlaugsson , J. Org. Chem. 2013, 78, 8312; 23899209 10.1021/jo4008942

[18] a) M. Carta , R. Malpass‐Evans , M. Croad , Y. Rogan , J. C. Jansen , P. Bernardo , F. Bazzarelli , N. B. McKeown , Science 2013, 339, 303; 23329042 10.1126/science.1228032

[19] W. Gong , M. Kazem‐Rostami , F. A. Son , S. Su , K. M. Fahy , H. Xie , T. Islamoglu , Y. Liu , J. F. Stoddart , Y. Cui , O. K. Farha , J. Am. Chem. Soc. 2022, 144, 22574. 36454651 10.1021/jacs.2c08623

[20] a) J. P. Riehl , F. S. Richardson , Chem. Rev. 1986, 86, 1;

[21] For selected examples of recent CPL‐emissive systems, see:

[22] a) L. E. MacKenzie , R. Pal , Nat. Rev. Chem. 2021, 5, 109; 37117607 10.1038/s41570-020-00235-4

[23] a) D. Parker , J. D. Fradgley , K.‐L. Wong , Chem. Soc. Rev. 2021, 50, 8193; 34075982 10.1039/d1cs00310k

[24] a) D.‐W. Zhang , M. Li , C.‐F. Chen , Chem. Soc. Rev. 2020, 49, 1331; 31999286 10.1039/c9cs00680j

[25] a) W. Dai , Y. Wang , R. Li , Y. Fan , G. Qu , Y. Wu , Q. Song , J. Han , S. Xiao , ACS Nano. 2020, 14, 17063; 33231424 10.1021/acsnano.0c06463

[26] T. Mori , Circularly Polarized Luminescence of Isolated Small Organic Molecules, 1st ed., Springer Nature, Singapore 2020.

[27] For selected examples of CPL‐emissive molecules with point chiral asymmetric carbon atoms, see:

[28] C. Qian , Y. Chen , Q. Zhao , M. Cheng , C. Lin , J. Jiang , L. Wang , Beilstein J. Org. Chem. 2021, 17, 52. 33488831 10.3762/bjoc.17.6 PMC7801797

[29] Y. Yoshigoe , H. Shimada , T. Takaki , Y. Imai , S. Saito , Chem.‐Eur. J. 2024, 30, 202304059. 10.1002/chem.202400674 38441521

[30] J. Jensen , K. Wärnmark , Synthesis 2001, 12, 1873.

[31] Y. Ishida , H. Ito , D. Mori , K. Saigo , Tetrahedron Lett. 2005, 46, 109.

[32] N. Miyaura , A. Suzuki , Chem. Rev. 1995, 95, 2457.

[33] N. Kitamura , E. Sakuda , Y. Iwahashi , K. Tsuge , Y. Sasaki , S. Ishizaka , J. Photochem. Photobiol. A. 2009, 207, 102.

[34] A. Stockmann , J. Kurzawa , N. Fritz , N. Acar , S. Schneider , J. Daub , R. Engl , T. Clark , J. Phys. Chem. A. 2002, 106, 7958.

[35] M. Belletête , S. Beaupré , J. Bouchard , P. Blondin , M. Leclerc , G. Durocher , J. Phys. Chem. B 2000, 104, 9118.

[36] CSP‐HPLC has been frequently employed for the resolution of Tröger’s Base derivatives, see the following selected examples:

[37] For the racemic resolution of **5** by co‐crystallization, see:

[38] a) P. Pisani , P. Piro , S. Decherchi , G. Bottegoni , D. Sona , V. Murino , W. Rocchia , A. Cavalli , J. Chem. Theory Comput. 2014, 10, 2557; 26580776 10.1021/ct400947t

[39] a) M. Monti , M. Stener , M. Aschi , J. Comput. Chem. 2022, 43, 2023; 36134712 10.1002/jcc.27001 PMC9825941

[40] Note that the glum values are calculated as *g* _lum_ = 2(I^L^−I^R^)/(I^L^+I^R^), where I^L^ and I^R^ correspond to the intensities of left‐ and right‐handed circularly polarized light.

[41] *B* _CPL_ is defined as *B* _CPL_ = ɛ_λ_ × Φ_F_ × |*g* _lum_|/2, being ɛ_λ_, the molar extinction coefficient at the excitation wavelength λ; Φ_F_, the fluorescence quantum yield; and |*g* _lum_|, the emission dissymmetry factor. See ref. 42.

[42] L. Arrico , L. Di Bari , F. Zinna , Chem.‐Eur. J. 2020, 27, 2920. 32725832 10.1002/chem.202002791

[43] a) R. G. Uceda , C. M. Cruz , S. Míguez‐Lago , L. Álvarez de Cienfuegos , G. Longhi , D. A. Pelta , P. Novoa , A. J. Mota , J. M. Cuerva , D. Miguel , Angew. Chem., Int. Ed. 2024, 63, 202316696; 10.1002/anie.202316696 38051776

[44] For selected examples of CPL switching with discrete molecules, see:

[45] Although protonation results in many cases in a red‐shift of the photoluminescence band respect to that of the non‐protonated species on account of an increased electron‐withdrawing character, there are reported examples of fluorophores functionalized with aniline groups that undergo hypsochromic shifts upon protonation. See for example:

[46] Note that photodegradation was observed for compound **3b** in acidic medium after exhaustive irradiation during the CPL measurements.

[47] a) H. J. Eckvahl , N. A. Tcyrulnikov , A. Chiesa , J. M. Bradley , R. M. Youn , S. Carretta , M. D. Krzyaniak , M. R. Wasielewski , Science 2023, 382, 197; 37824648 10.1126/science.adj5328

