# Supplementary material for: Acid/Base‐Responsive Circularly Polarized Luminescence Emitters with Configurationally Stable Nitrogen Stereogenic Centers
Source: Adv Mater. 2025 May 15;37(29):2417326. doi: 10.1002/adma.202417326 (PMC12288784; doi:10.1002/adma.202417326)
Supplement: Supplementary file 1 — Supporting Information [file ADMA-37-2417326-s001.pdf]

ADVANCED MATERIALS

Supporting Information

for *Adv. Mater.*, DOI 10.1002/adma.202417326

Acid/Base-Responsive Circularly Polarized Luminescence Emitters with Configurationally Stable Nitrogen Stereogenic Centers

Pablo García-Cerezo, Marcos D. Codesal, Arthur H. G. David, Laura Le Bras, Seifallah Abid, Xuesong Li, Delia Miguel, Masoud Kazem-Rostami, Benoît Champagne, Araceli G. Campaña, J. Fraser Stoddart* and Victor Blanco**

Supporting Information

Acid/Base-Responsive Circularly Polarized Luminescence Emitters with Configurationally Stable Nitrogen Stereogenic Centers

Pablo García-Cerezo,[1,○] Marcos D. Codesal,[1,○] Arthur H. G. David,[2,3] Laura Le Bras,[4,*] Seifallah Abid,[2] Xuesong Li,[2,5] Delia Miguel,[6] Masoud Kazem-Rostami,[2] Benoît Champagne,[7] Araceli G. Campaña,[1] J. Fraser Stoddart,[2,8,9,10,11,12,*,†] Victor Blanco,[1,*]

[1] *Departamento de Química Orgánica, Facultad de Ciencias, Unidad de Excelencia de Química Aplicada a Biomedicina y Medioambiente (UEQ), Universidad de Granada (UGR), Avda. Fuente Nueva S/N, 18071 Granada, Spain*

[2] *Department of Chemistry, Northwestern University, 2145 Sheridan Road, Evanston, IL 60208, USA*

[3] *Laboratoire MOLTECH-Anjou (UMR CNRS 6200), Université Angers, 2 Bd Lavoisier, 49045 Angers Cedex, France*

[4] *Université Marie et Louis Pasteur, CNRS, Chrono-environnement (UMR 6249), F-25000, Besançon, France*

[5] *Department of Chemistry, University of Wyoming, Laramie, WY 82072, USA*

[6] *Nanoscopy-UGR Laboratory. Physical Chemistry Department, UEQ, Faculty of Pharmacy, University of Granada, C. U. Cartuja, 18071 Granada, Spain*

[7] *Laboratory of Theoretical Chemistry, Namur Institute of Structured Matter (NISM), University of Namur, rue de Bruxelles, 61, 5000 Namur, Belgium*

[8] *Department of Chemistry, The University of Hong Kong, Hong Kong SAR, 999077, China*

[9] *Stoddart Institute of Molecular Science, Department of Chemistry, Zhejiang University, Hangzhou 310027, China*

[10] *ZJU-Hangzhou Global Scientific and Technological Innovation Center Hangzhou 311215, China*

[11] *Center for Regenerative Medicine and Department of Medicine, Northwestern University, 303 East Superior Street, Chicago, IL 60611, USA*

[12] *School of Chemistry, University of New South Wales, Sydney, NSW 2052, Australia*

○ *P.G.-C. and M.D.C. contributed equally to this paper*

† *Sir Fraser Stoddart passed away on 30 December 2024.*

*E-mail: laura.le_bras@univ-fcomte.fr; stoddart@hku.hk; victorblancos@ugr.es

Table of Contents

1. Materials and Methods **S2**

2. Synthetic Protocols and Characterization Details **S4**

3. NMR Spectroscopy **S11**

4. Chiral HPLC Traces **S21**

5. Photophysical Properties **S32**

6. Computational Methods **S68**

7. References **S76**

1. Materials and Methods

All solvents and reagents were obtained commercially and used without further purification unless noted. Anhydrous DMF was obtained using a solvent drying system. Water was deionized and micro-filtered using Milli-Q water filtration station.

Thin-layer chromatography (TLC) was performed on silica gel 60 F_{254} (E Merck). Developed plates were visualized using UV light at wavelengths of 254 and 365 nm. Normal-phase column chromatography was carried out either on silica gel 60F (Merck 9385, 0.040 − 0.063 mm), neutral alumina (Brockmann I, Sigma-Aldrich) or CombiFlash® Automation Systems (Rf 200). Organic solvents were concentrated under reduced pressure on a Büchi rotary evaporator using a water bath.

Nuclear magnetic resonance (NMR) Spectra were recorded in $CDCl_3$ or CD_2Cl_2 on Bruker Avance III 500 MHz, Bruker Avance III HD NanoBay 400 MHz, or Bruker Avance Neo 500 MHz spectrometers. Chemical shifts are reported in ppm relative to the signals corresponding to the residual non-deuterated solvents ($CDCl_3$: δ_H = 7.26 and δ_C = 77.16 ppm; CD_2Cl_2: δ_H = 5.32). Data for ^{1}H NMR spectra are reported as follows: chemical shift (δ ppm), multiplicity (s = singlet, d = doublet, m = multiplet, br = broad, dd = doublet of doublets), integration, coupling constant (*J*, Hz) and assignment. Data for ^{13}C NMR spectra are reported in terms of chemical shift.

High resolution mass spectrometry (HRMS) was measured on an Agilent LCMS 6545 or a Waters XEVO G2-XS QTof mass spectrometer.

Absorption spectra were recorded in an Agilent Cary 60 spectrophotometer, fixing 1 nm as data interval and 0.1 s as average time, performing in all the cases a baseline correction, or in an Analytik Jena Specord 200 plus. Steady-state emission spectra were collected using a 10×10 mm cuvette (Hellma 105.201-QS) in a Jasco FP-8300 spectrofluorometer (Jasco, Tokyo, Japan), with 2.5 nm slit-width both in the excitation and emission and 1 s as integration time. Electronic circular dichroism (ECD) and circularly polarized luminescence (CPL) spectra were recorded in an Olis DSM172 spectrophotometer equipped with a xenon lamp of 150 W.

2. Synthetic Protocols and Characterization Details

Synthesis of compound **5**

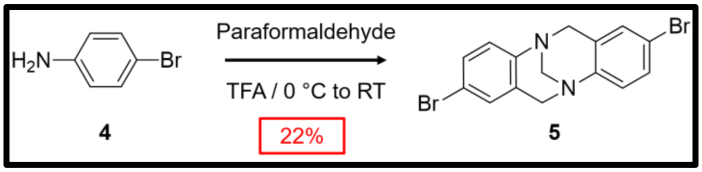

Compound **5** was synthesized according to Wärnmark's procedure:[S1] To trifluoroacetic acid (100 mL) under N_2, was slowly added 4-bromoaniline (6.88 g, 40.0 mmol). Once dissolved, the resulting solution was cooled to 0 °C with a water-ice bath and paraformaldehyde (1.80 g, 59.9 mmol) was added. The mixture was allowed to warm up to r.t. and further stirred for 5 d at r.t. The reaction mixture was then poured on ice (200 g), basified by addition of NH_3 (300 mL) and extracted with CH_2Cl_2 (2 × 300 mL). The combined organic layers were washed with H_2O (100 mL), dried over anhydrous Na_2SO_4, and the solvent was removed under reduced pressure. The crude material was further purified by column chromatography (SiO_2, CH_2Cl_2) to yield **5** (1.67 g, 22%) as a yellow solid. ^{1}H NMR (500 MHz, $CDCl_3$) δ = 7.27 (dd, J = 8.6, 2.3 Hz, 2H), 7.04 (d, J = 2.3 Hz, 2H), 7.00 (d, J = 8.6 Hz, 2H), 4.64 (d, J = 16.8 Hz, 2H), 4.25 (s, 2H), 4.09 (d, J = 16.8 Hz, 2H). ^{13}C NMR (126 MHz, $CDCl_3$) δ = 146.8, 130.8, 129.8, 129.7, 126.8, 117.0, 66.7, 58.4. HRMS (ESI): m/z = 378.9442 $[M + H]^+$ (calcd for $C_{15}H_{13}N_2Br_2$, m/z = 378.9440). Spectral data agree with those previously reported.[S1]

(*RR*)-**5** was obtained by racemic resolution by co-crystallization with *O,O'*-dibenzoyl-L-tartaric acid followed by extraction with $Na_2CO_{3(aq)}$ (5%) as previously reported.[S2] The crystallization step was reported on a multi-gram scale (11.6 g of *rac*-**5**) but was repeated in our case on a smaller scale (500 mg of *rac*-**5**) following the reported procedure: To *O,O'*-dibenzoyl-L-tartaric acid (2*R*,3*R*)-2,3-bis(benzoyloxy)succinic acid) (484 mg, 1.35 mmol) 1,2-dichloroethane (5 mL) was added and the mixture was refluxed to remove the residual water using a Dean-Stark apparatus. *Rac*-**5** (500 mg, 1.32 mmol) was added, and the solution was heated to reflux. The suspension was further stirred at 60 ºC for 5 d. The mixture was cooled to r.t. and left overnight. The precipitate was filtered under vacuum and washed with 1,2-dichloroethane (3 × 5 mL). The solid was transferred to a round-bottom flask, 1,2-dichloroethane (4 mL) were added and the mixture was stirred for 30 min and filtered again under vacuum, washing with 1,2-dichloroethane (2 × 5 mL). A white solid (715 mg) was obtained. The extraction step was carried out as follows: The solid was dissolved in a mixture of CH_2Cl_2 (20 mL) and $Na_2CO_{3(aq)}$ (5%) (20 mL) and the extraction was carried out in a separatory funnel. The phases were separated, and the aqueous layer was further extracted with CH_2Cl_2 (2 ×15 mL). The organic phase was dried with anhydrous Na_2SO_4 and the solvent removed under reduced

pressure to give (*RR*)-**5** (360 mg, 72%) as a white solid. ^{1}H NMR (400 MHz, $CDCl_3$) δ = 7.27 (dd, J = 8.7, 2.3 Hz, 2H), 7.04 (d, J = 2.3 Hz, 2H), 7.00 (d, J = 8.7 Hz, 2H), 4.63 (d, J = 16.8 Hz, 2H), 4.23 (s, 2H), 4.09 (d, J = 16.8 Hz, 2H). ^{13}C NMR (126 MHz, CD_2Cl_2) δ = 147.5, 130.7, 130.4, 130.0, 127.2, 116.7, 67.0, 58.7. HRMS (ESI): *m/z* = 378.9446 [*M* + H]$^+$ (calcd for $C_{15}H_{13}N_2Br_2$, *m/z* = 378.9440). $[\alpha]_D^{20}$ = −379 (*c*, 0.117, $CHCl_3$). The ee was determined by CSP-HPLC (Chiralpak® IC, see section 4.2, Figure S27): *ee* = 97.1%; *er* = 98.6:1.4.

Synthesis of compound **6**

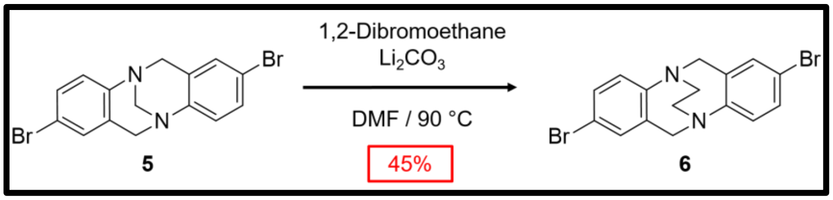

Compound **6** was synthesized according to Saigo's procedure:[S3] 1,2-Dibromoethane (2.35 g, 12.5 mmol), and Li_2CO_3 (1.80 g, 25.0 mmol) were added to a solution of **5** (1.90 g, 5.0 mmol) in anhydrous DMF (25 mL) under N_2. The resulting mixture was stirred for 12 h at 90 °C. The solvent was evaporated under vacuum and the resulting solid was dissolved in CH_2Cl_2 (100 mL) and washed with H_2O (5 × 50 mL). The organic layer was dried over anhydrous Na_2SO_4 and the solvent was removed under reduced pressure. The crude material was further purified by column chromatography (SiO_2, CH_2Cl_2) to afford **6** (890 mg, 45%) as an orange solid. ^{1}H NMR (500 MHz, $CDCl_3$) δ = 7.17 (dd, J = 8.4, 2.3 Hz, 2H), 7.04 (d, J = 2.3 Hz, 2H), 6.96 (d, J = 8.4 Hz, 2H), 4.53 (d, J = 17.4 Hz, 2H), 4.35 (d, J = 17.4 Hz, 2H), 3.55 (m, 4H). ^{13}C NMR (126 MHz, $CDCl_3$) δ = 149.1, 138.7, 131.4, 130.4, 129.7, 117.9, 58.6, 54.6. HRMS (ESI): *m/z* = 392.9598 [*M* + H]$^+$ (calcd for $C_{16}H_{15}N_2Br_2$, *m/z* = 392.9597).

Synthesis of compound **1a**

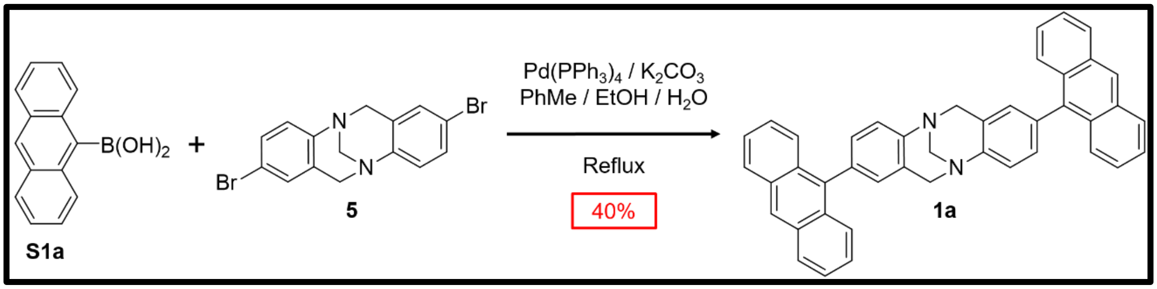

1a: In a 100 mL round bottom flask under N_2 atmosphere were introduced **5** (69 mg, 0.18 mmol), 9-anthraceneboronic acid (**S1a**, 322 mg, 1.45 mmol), $Pd(PPh_3)_4$ (105 mg, 0.091 mmol) and K_2CO_3 (302 mg, 2.18 mmol) successively. Then, a degassed solution of PhMe, H_2O and EtOH (10:1:1, 12 mL) was added

and the mixture was refluxed for 18 h. After evaporation of solvents, the crude was dissolved in CH_2Cl_2 (50 mL) and washed with H_2O (50 mL). The aqueous layer was extracted with CH_2Cl_2 (2 × 50 mL). The combined organic layers were dried over anhydrous Na_2SO_4 and the solvent was removed under reduced pressure. The crude material was further purified by column chromatography (SiO_2, CH_2Cl_2 then CH_2Cl_2/EtOAc 9:1) to give **1a** (42 mg, 40%) as a white solid. ^{1}H NMR (500 MHz, $CDCl_3$) δ = 8.65 (s, 2H), 8.21 (d, J = 8.4 Hz, 4H), 7.91 (d, J = 8.8 Hz, 2H), 7.83 (d, J = 8.8 Hz, 2H), 7.65 – 7.42 (m, 14H), 5.09 (d, J = 16.9 Hz, 2H), 4.77 (s, 2H), 4.60 (d, J = 16.9 Hz, 2H). ^{13}C NMR (126 MHz, $CDCl_3$) δ = 147.4, 136.7, 134.6, 131.5, 131.5, 130.6, 130.5, 130.4, 129.8, 128.6, 128.5, 127.9, 127.0, 126.8, 126.7, 125.5, 125.5, 125.3, 125.3, 125.2, 67.3, 58.8. HRMS (APPI): m/z = 575.2495 [M + H]$^+$ (calcd for $C_{43}H_{31}N_2$, m/z = 575.2482).

Synthesis of compound **1b**

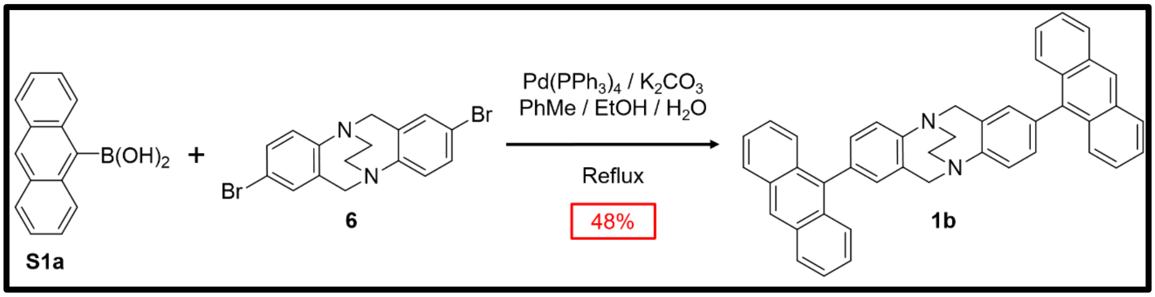

1b: In a 100 mL round bottom flask under N_2 atmosphere were introduced **6** (67 mg, 0.17 mmol), 9-anthraceneboronic acid (**S1a**, 302 mg, 1.36 mmol), $Pd(PPh_3)_4$ (98 mg, 85 µmol) and K_2CO_3 (282 mg, 2.04 mmol) successively. Then, a degassed solution of PhMe, H_2O and EtOH (10:1:1, 12 mL) was added and the mixture was refluxed for 24 h. After evaporation of solvents, the crude was dissolved in CH_2Cl_2 (50 mL) and washed with H_2O (50 mL). The aqueous layer was extracted with CH_2Cl_2 (2 × 50 mL). The combined organic layers were dried over anhydrous Na_2SO_4 and the solvent was evaporated under vacuum. The crude material was further purified by column chromatography (SiO_2, CH_2Cl_2 then CH_2Cl_2/EtOAc 9:1) to afford **1b** (48 mg, 48%) as a white solid. ^{1}H NMR (500 MHz, $CDCl_3$) δ = 8.48 (s, 2H), 8.03 (m, 4H), 7.73 (br, 2H), 7.49 – 7.29 (m, 9H), 7.25 – 7.03 (m, 7H), 4.93 – 4.57 (br m, 4H), 4.06 – 3.70 (br m, 4H). ^{13}C NMR (126 MHz, $CDCl_3$) δ = 136.8, 135.1, 132.1, 131.5, 131.4, 130.4, 130.3, 128.5, 128.5, 127.8, 127.0, 126.6, 126.5, 125.7, 125.5, 125.3, 125.1, 59.7, 54.9. HRMS (ESI): m/z = 589.2643 [M + H]$^+$ (calcd for $C_{44}H_{33}N_2$, m/z = 589.2638).

Synthesis of compound **2a**

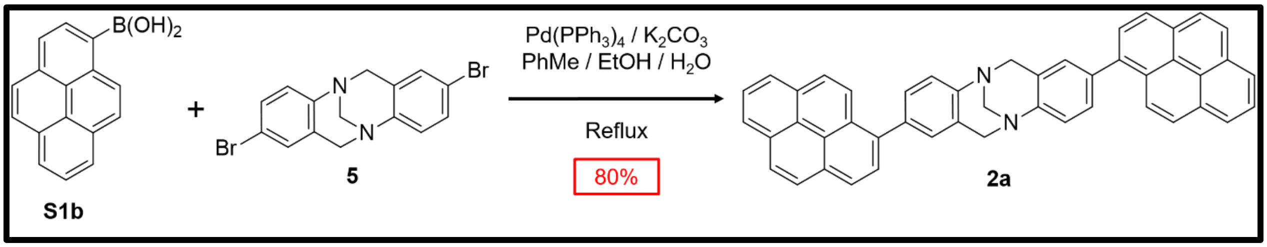

2a: In a 100 mL round bottom flask under N_2 atmosphere were introduced **5** (59 mg, 0.16 mmol), 1-pyrenylboronic acid (**S1b**, 305 mg, 1.24 mmol), $Pd(PPh_3)_4$ (90 mg, 78 μmol) and K_2CO_3 (257 mg, 1.86 mmol) successively. Then, a degassed solution of PhMe, H_2O and EtOH (10:1:1, 12 mL) was added and the mixture was refluxed for 20 h. After evaporation of solvents, the crude was dissolved in CH_2Cl_2 (50 mL) and washed with H_2O (50 mL). The aqueous layer was extracted with CH_2Cl_2 (2 × 50 mL). The combined organic layers were dried over anhydrous Na_2SO_4 and the solvent was evaporated under reduced pressure. The crude material was further purified by column chromatography (SiO_2, CH_2Cl_2 then CH_2Cl_2/EtOAc 9:1) to yield **2a** (77 mg, 80%) as a yellow solid. ^{1}H NMR (500 MHz, $CDCl_3$) δ = 8.23 – 8.15 (m, 8H), 8.09 (s, 4H), 8.05 – 7.99 (m, 4H), 7.94 (d, J = 7.8 Hz, 2H), 7.52 (dd, J = 8.3, 2.0 Hz, 2H), 7.42 (d, J = 8.3 Hz, 2H), 7.29 (d, J = 2.0 Hz, 2H), 4.97 (d, J = 16.9 Hz, 2H), 4.59 (s, 2H), 4.46 (d, J = 16.9 Hz, 2H). ^{13}C NMR (126 MHz, $CDCl_3$) δ = 146.8, 137.6, 137.2, 131.6, 131.1, 130.7, 130.1, 129.2, 128.6, 127.7, 127.7, 127.6, 127.5, 126.2, 125.3, 125.3, 125.2, 125.1, 125.1, 125.0, 124.8, 67.2, 58.8. HRMS (ESI): m/z = 623.2489 $[M + H]^+$ (calcd for $C_{47}H_{31}N_2$, m/z = 623.2482).

(*RR*)-**2a**: In a 100 mL round bottom flask under N_2 atmosphere were introduced (*RR*)-**5** (48 mg, 0.13 mmol), 1-pyrenylboronic acid (**S1b**, 250 mg, 1.01 mmol), $Pd(PPh_3)_4$ (23 mg, 20 μmol) and K_2CO_3 (193 mg, 1.39 mmol) successively. Then, a degassed solution of PhMe, H_2O and EtOH (10:1:1, 10 mL) was added and the mixture was refluxed for 18 h. After evaporation of solvents, the crude was dissolved in CH_2Cl_2 (50 mL) and washed with H_2O (50 mL). The aqueous layer was extracted with CH_2Cl_2 (2 × 50 mL). The combined organic layers were dried over anhydrous Na_2SO_4 and the solvent was evaporated under reduced pressure. The crude material was further purified by column chromatography (Al_2O_3, neutral, Brockmann I, CH_2Cl_2) to yield (*RR*)-**2a** (76 mg, 80%) as a pale yellow solid. The characterization data matched that of *rac*-**2a**. ^{1}H NMR (400 MHz, CD_2Cl_2) δ = 8.21 – 8.14 (m, 8H), 8.07 (s, 4H), 8.02 – 7.98 (m, 4H), 7.91 (d, J = 7.9 Hz, 2H), 7.47 (dd, J = 8.1, 2.2 Hz, 2H), 7.34 (d, J = 8.2 Hz, 2H), 7.26 (d, J = 2.2 Hz, 2H), 4.89 (d, J = 16.8 Hz, 2H), 4.50 (s, 2H), 4.41 (d, J = 16.8 Hz, 2H). ^{13}C NMR (101 MHz, CD_2Cl_2) δ = 148.2, 137.7, 137.1, 131.9, 131.4, 130.8, 130.1, 129.3, 128.8, 128.6, 128.0, 127.77, 127.75, 127.7, 126.4, 125.6, 125.5,

125.5, 125.3, 125.21, 125.16, 125.1, 67.5, 59.3. HRMS (ESI): *m/z* = 623.2491 [*M* + H]$^+$ (calcd for $C_{47}H_{31}N_2$, *m/z* = 623.2482). $[\alpha]_D^{20} = -662$ (*c*, 0.36, $CHCl_3$). The ee was determined by CSP-HPLC (Chiralpak® IC, see section 4.2, Figure S28): *ee* = 94.6%; *er* = 97.3:2.7.

Synthesis of compound **2b**

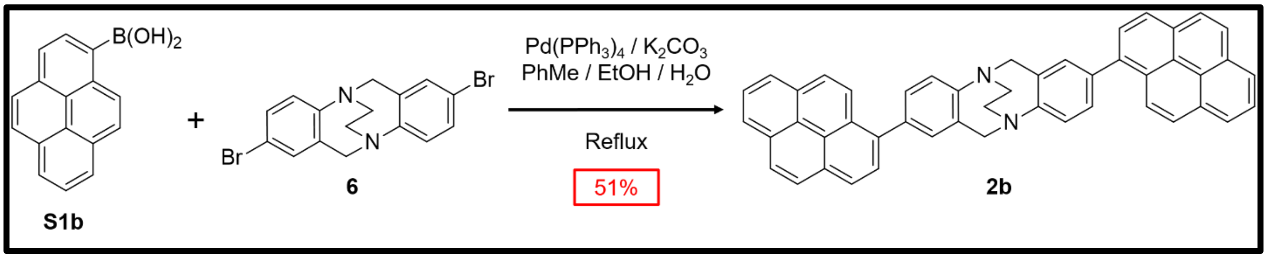

2b: In a 100 mL round bottom flask under N_2 atmosphere were introduced **6** (61 mg, 0.16 mmol), 1-pyrenylboronic acid (**S1b**, 305 mg, 1.24 mmol), $Pd(PPh_3)_4$ (90 mg, 78 µmol) and K_2CO_3 (257 mg, 1.86 mmol) successively. Then, a degassed solution of PhMe, H_2O and EtOH (10:1:1, 12 mL) was added and the mixture was refluxed for 20 h. After evaporation of solvents, the crude was dissolved in CH_2Cl_2 (50 mL) and washed with H_2O (50 mL). The aqueous layer was extracted with CH_2Cl_2 (2 × 50 mL). The combined organic layers were dried over anhydrous Na_2SO_4 and the solvent was evaporated under reduced pressure. The crude material was further purified by column chromatography (SiO_2, CH_2Cl_2 then CH_2Cl_2/EtOAc 9:1) to give **2b** (50 mg, 51%) as a yellow solid. ^{1}H NMR (500 MHz, $CDCl_3$) δ = 8.20 – 8.11 (m, 8H), 8.07 (s, 4H), 8.03 – 7.96 (m, 4H), 7.93 (d, *J* = 7.8 Hz, 2H), 7.41 (dd, *J* = 8.0, 2.1 Hz, 2H), 7.36 (d, *J* = 8.0 Hz, 2H), 7.29 (d, *J* = 2.1 Hz, 2H), 4.84 (d, *J* = 17.3 Hz, 2H), 4.71 (d, *J* = 17.3 Hz, 2H), 3.92 – 3.70 (m, 4H). ^{13}C NMR (126 MHz, $CDCl_3$) δ = 149.5, 141.0, 137.8, 137.4, 136.8, 131.6, 131.1, 131.1, 130.6, 129.8, 128.6, 128.1, 127.7, 127.5, 127.5, 126.1, 125.4, 125.2, 125.1, 125.0, 124.9, 124.7, 59.6, 55.0. HRMS (ESI): *m/z* = 637.2646 [*M* + H]$^+$ (calcd for $C_{48}H_{33}N_2$, *m/z* = 637.2638).

Synthesis of compound **3a**

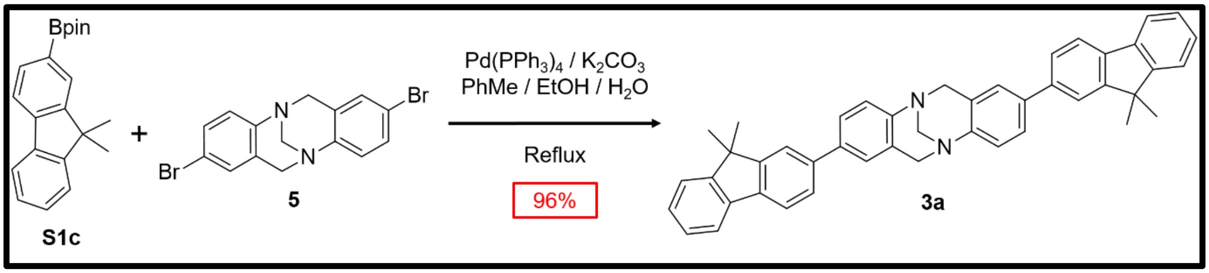

3a: In a 100 mL two necked round bottom flask under N_2 atmosphere were introduced **5** (59 mg, 0.16 mmol), 2-(9,9-dimethyl-9H-fluoren-2-yl)-4,4,5,5-tetramethyl-1,3,2-dioxaborolane (**S1c**, 400 mg, 1.24 mmol), $Pd(PPh_3)_4$ (90 mg, 78 µmol) and K_2CO_3 (257 mg, 1.86 mmol) successively. Then, a degassed solution of PhMe, H_2O and EtOH (10:1:1, 12 mL) was added and the mixture was refluxed for 24 h. After evaporation of solvents, the crude was dissolved in CH_2Cl_2 (20 mL) and washed with H_2O (20 mL) and brine (20 mL). The organic layer was collected, dried over anhydrous Na_2SO_4 and the solvent was evaporated under reduced pressure. The crude material was further purified by column chromatography (SiO_2, CH_2Cl_2/EtOAc 9:1) to afford **3a** (90 mg, 96%) as a white solid. ^{1}H NMR (500 MHz, $CDCl_3$) δ = 7.72 (m, 4H), 7.53 – 7.42 (m, 8H), 7.32 (m, 6H), 7.24 (d, J = 2.1 Hz, 2H), 4.87 (d, J = 16.6 Hz, 2H), 4.48 (s, 2H), 4.36 (d, J = 16.6 Hz, 2H), 1.50 (s, 6H), 1.49 (s, 6H). ^{13}C NMR (126 MHz, $CDCl_3$) δ =154.4, 153.9, 139.8, 138.9, 138.5, 127.4, 127.2, 126.7, 126.0, 125.8, 125.5, 122.7, 121.3, 121.3, 120.4, 120.2, 67.2, 58.9, 47.0, 27.3. HRMS (APPI): m/z = 607.3119 $[M + H]^+$ (calcd for $C_{45}H_{39}N_2$, m/z = 607.3108).

Synthesis of compound **3b**

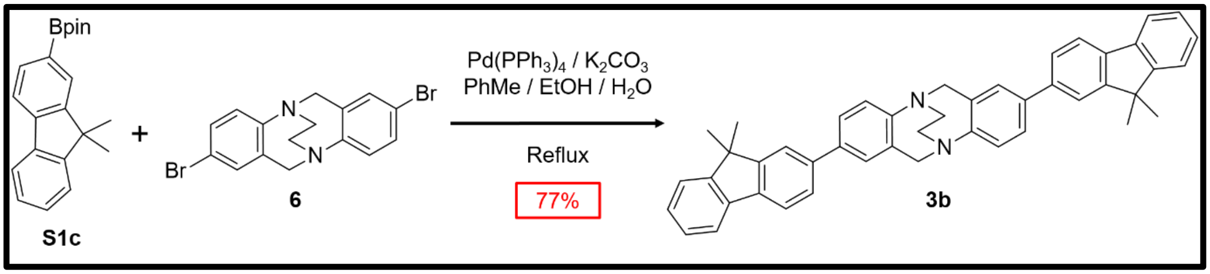

3b: In a 100 mL two necked round bottom flask under N_2 atmosphere were introduced **6** (59 mg, 0.16 mmol), 2-(9,9-dimethyl-9H-fluoren-2-yl)-4,4,5,5-tetramethyl-1,3,2-dioxaborolane (**S1c**, 400 mg, 1.24 mmol), $Pd(PPh_3)_4$ (90 mg, 78 µmol) and K_2CO_3 (257 mg, 1.86 mmol) successively. Then, a degassed solution of PhMe, H_2O and EtOH (10:1:1, 12 mL) was added and the mixture was refluxed for 24 h. After evaporation of solvents, the crude was dissolved in CH_2Cl_2 (20 mL) and washed with H_2O (20 mL) and brine (20 mL). The organic layer was collected, dried over anhydrous Na_2SO_4 and the solvent was removed under vacuum. The crude material was further purified by column chromatography (SiO_2, CH_2Cl_2/EtOAc 9:1) to yield **3b** (72 mg, 77%) as a white solid. ^{1}H NMR (500 MHz, $CDCl_3$) δ =7.71 (d, J = 7.9 Hz, 4H), 7.52 (d, J = 1.6 Hz, 2H), 7.44 – 7.24 (m, 14H), 4.75 (d, J = 17.2 Hz, 2H), 4.64 (d, J = 17.2 Hz, 2H), 3.72 (m, 4H), 1.49 (s, 6H), 1.48 (s, 6H). ^{13}C NMR (126 MHz, $CDCl_3$) δ =154.3, 153.9, 139.7, 139.0, 138.4,

128.5, 127.6, 127.3, 127.1, 126.2, 126.0, 122.7, 121.2, 120.3, 120.1, 59.5, 47.0, 27.4, 27.3. HRMS (ESI): *m/z* = 621.3277 [*M* + H]$^+$ (calcd for $C_{46}H_{41}N_2$, *m/z* = 621.3264).

3. NMR Spectroscopy

Compound **5**

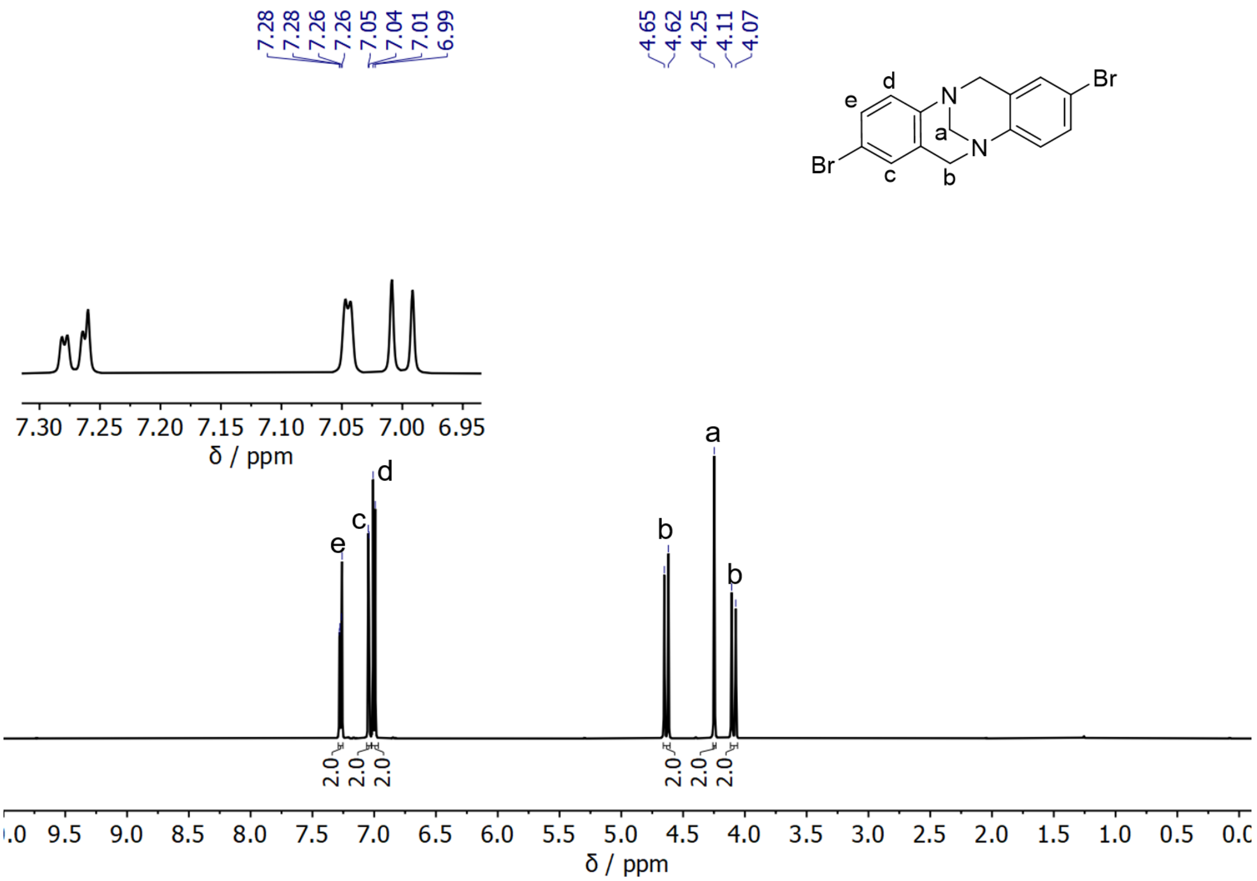

Figure S1. ^{1}H NMR (500 MHz, $CDCl_3$) spectrum of **5**.

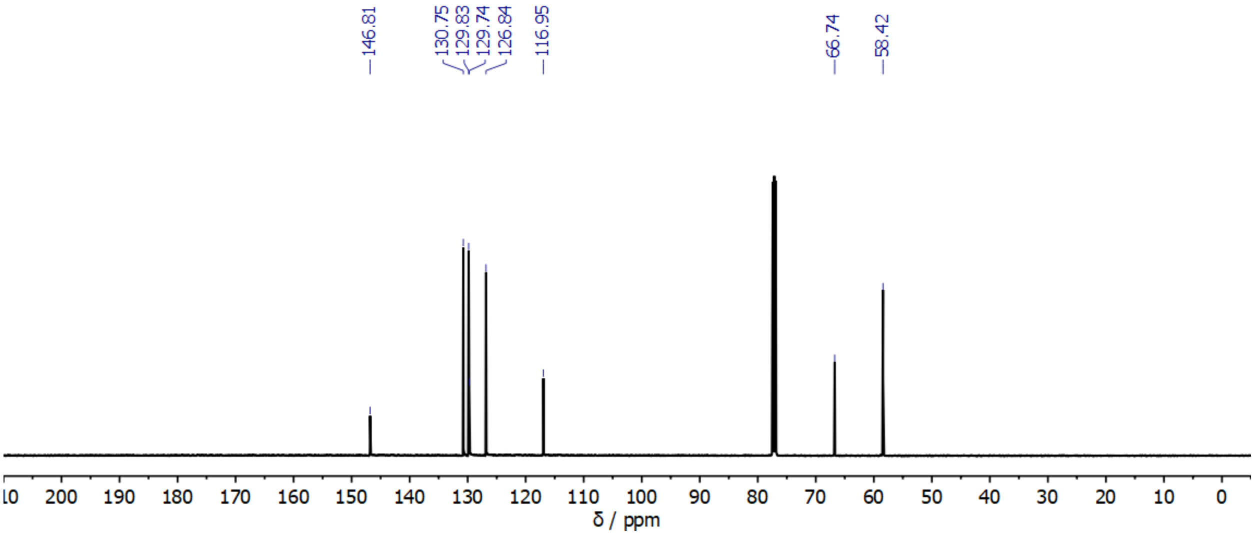

Figure S2. ^{13}C NMR (126 MHz, $CDCl_3$) spectrum of **5**.

Compound (*RR*)-**5**

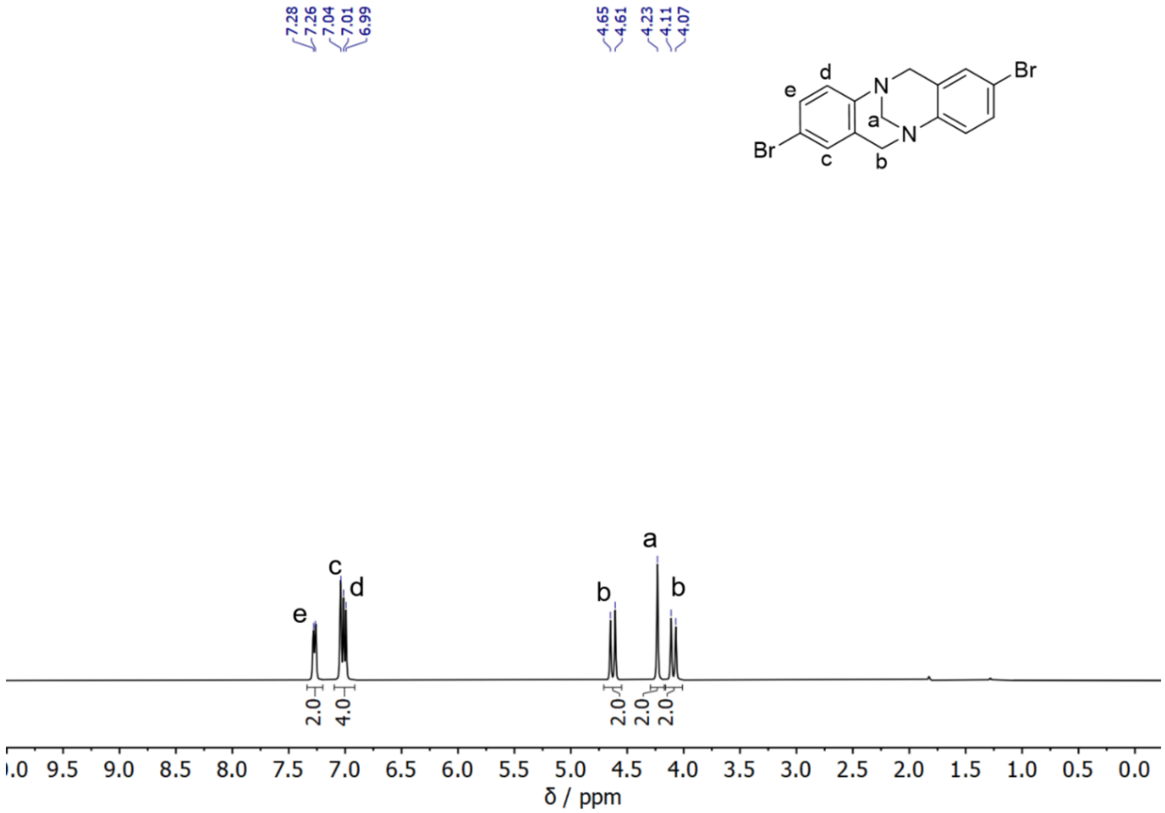

Figure S3. ^{1}H NMR (400 MHz, $CDCl_3$) spectrum of (*RR*)-**5**.

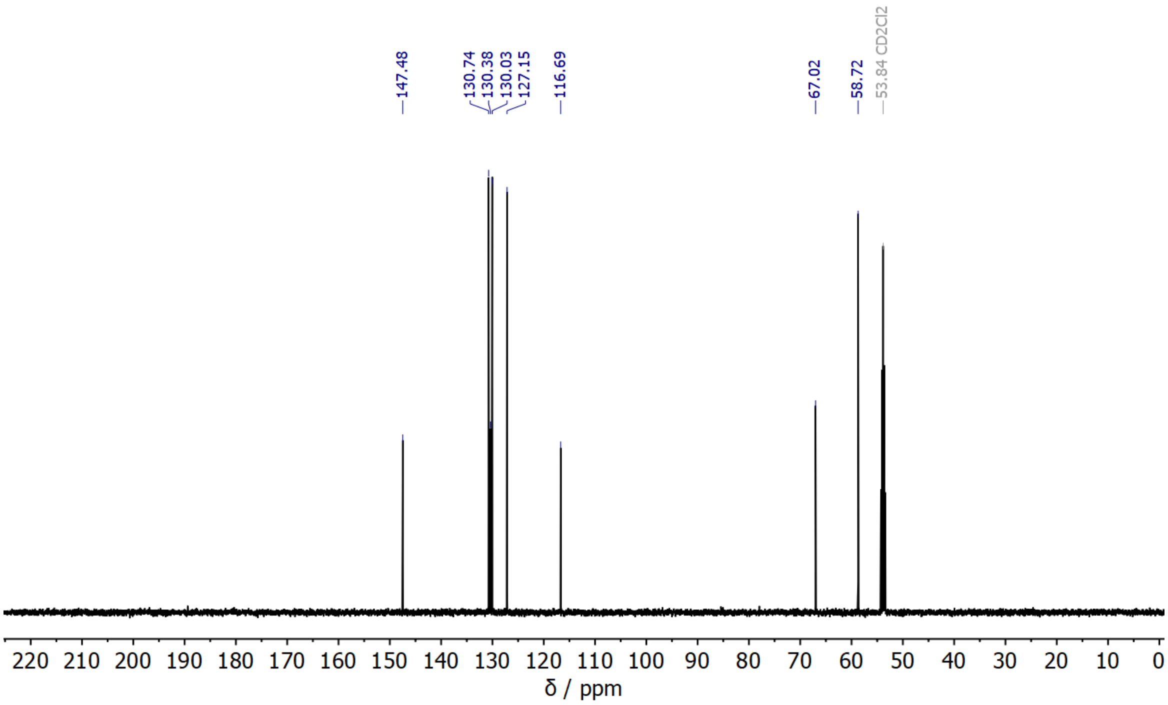

Figure S4. ^{13}C NMR (126 MHz, CD_2Cl_2) spectrum of (*RR*)-**5**.

Compound **6**

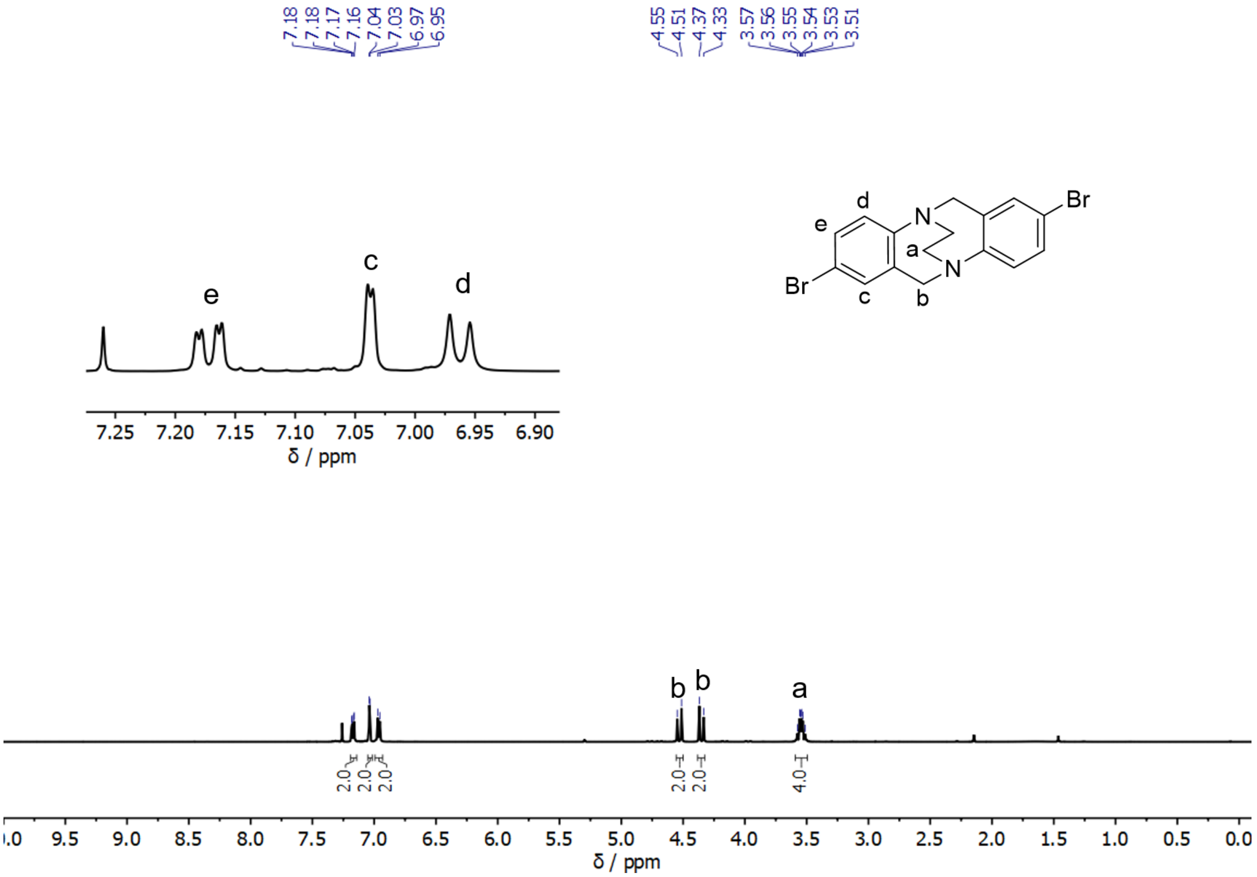

Figure S5. ^{1}H NMR (500 MHz, $CDCl_3$) spectrum of **6**.

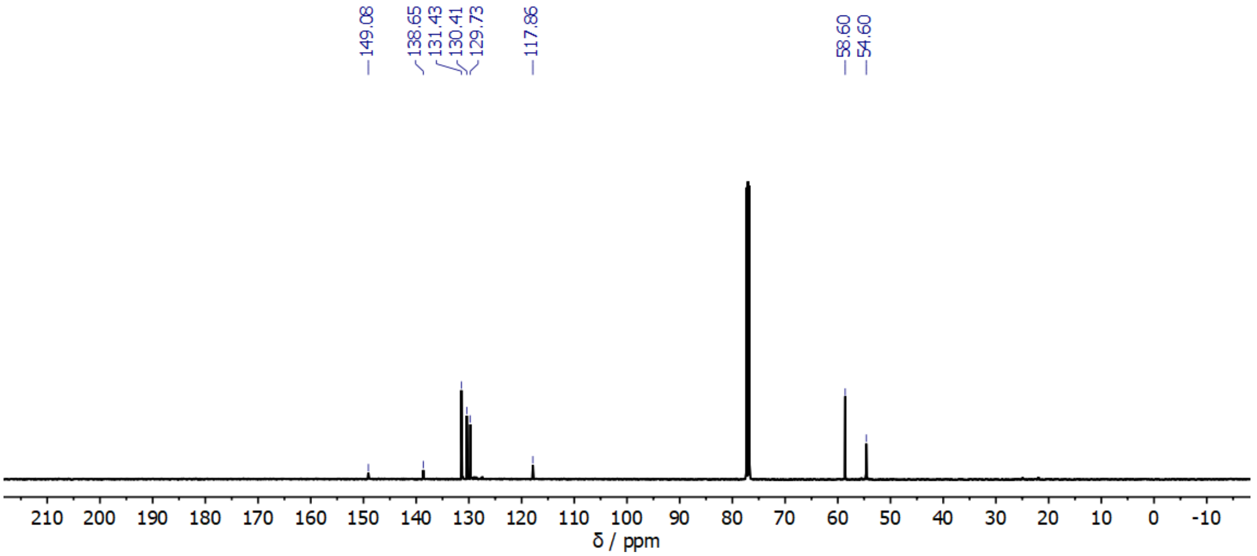

Figure S6. ^{13}C NMR (126 MHz, $CDCl_3$) spectrum of **6**.

Compound **1a**

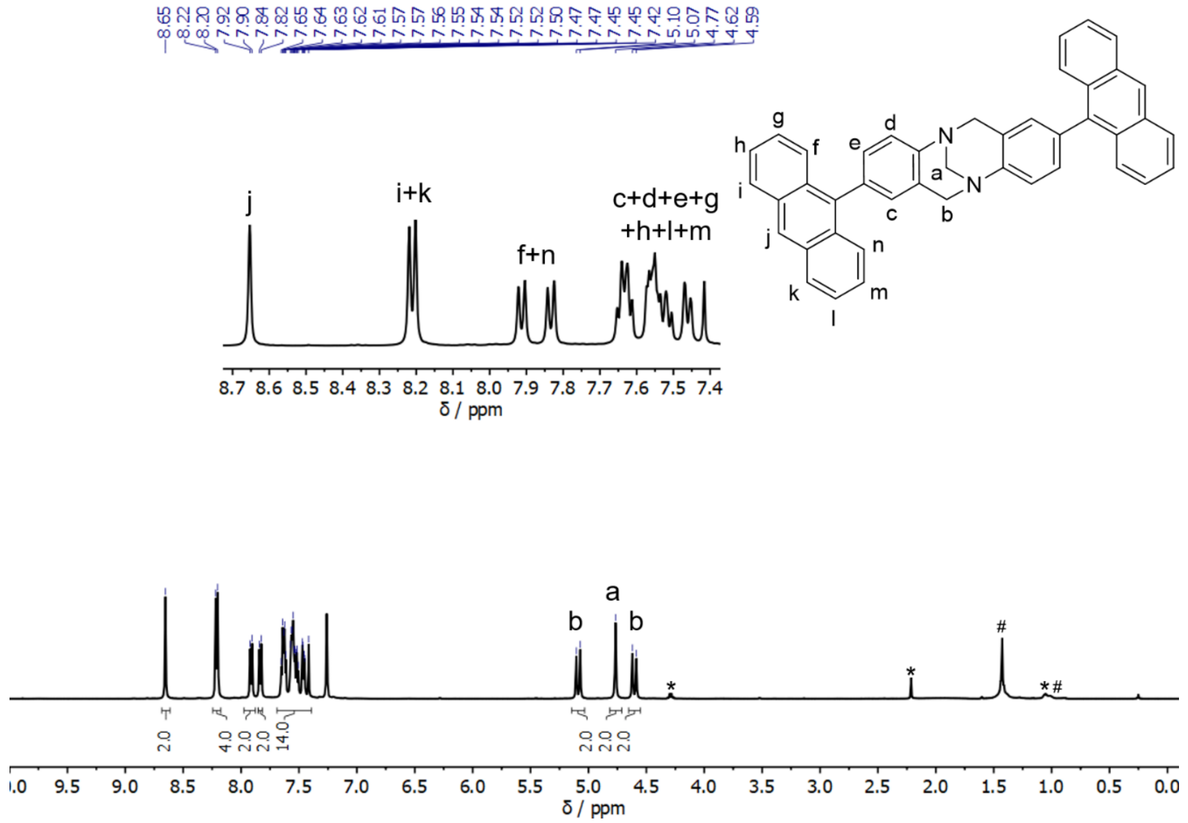

Figure S7. ^{1}H NMR (500 MHz, $CDCl_3$) spectrum of **1a**. * and # indicate residual EtOAc solvent and grease, respectively.

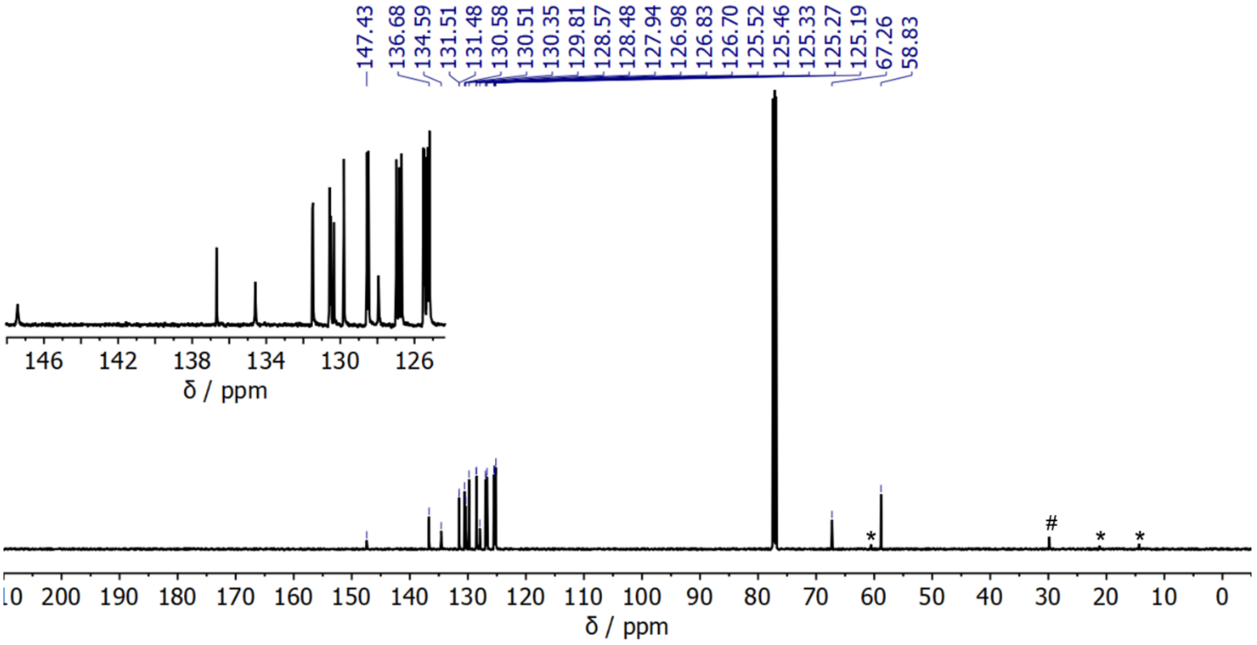

Figure S8. ^{13}C NMR (126 MHz, $CDCl_3$) spectrum of **1a**. * and # indicate residual EtOAc solvent and grease, respectively.

Compound **1b**

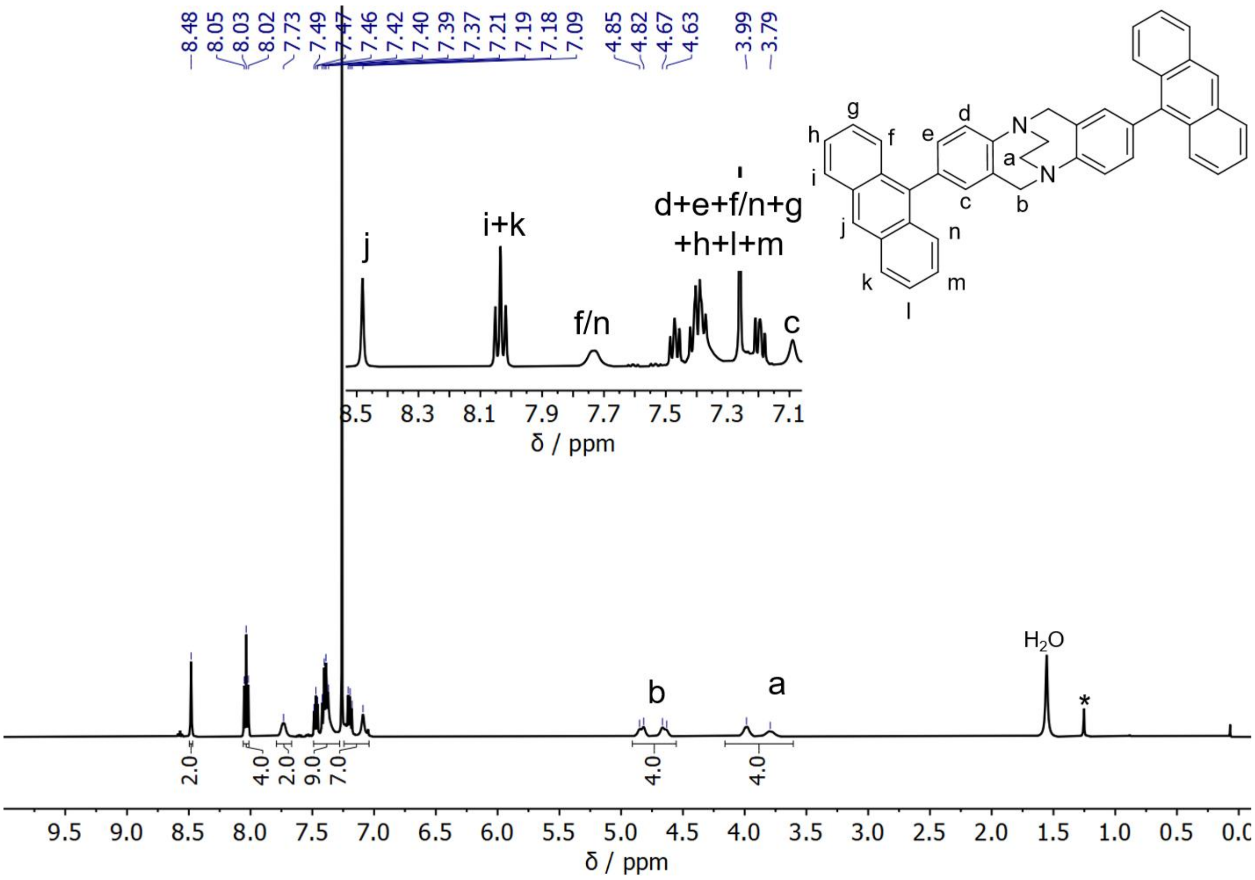

Figure S9. ^{1}H NMR (500 MHz, $CDCl_3$) spectrum of **1b**. * indicates residual grease.

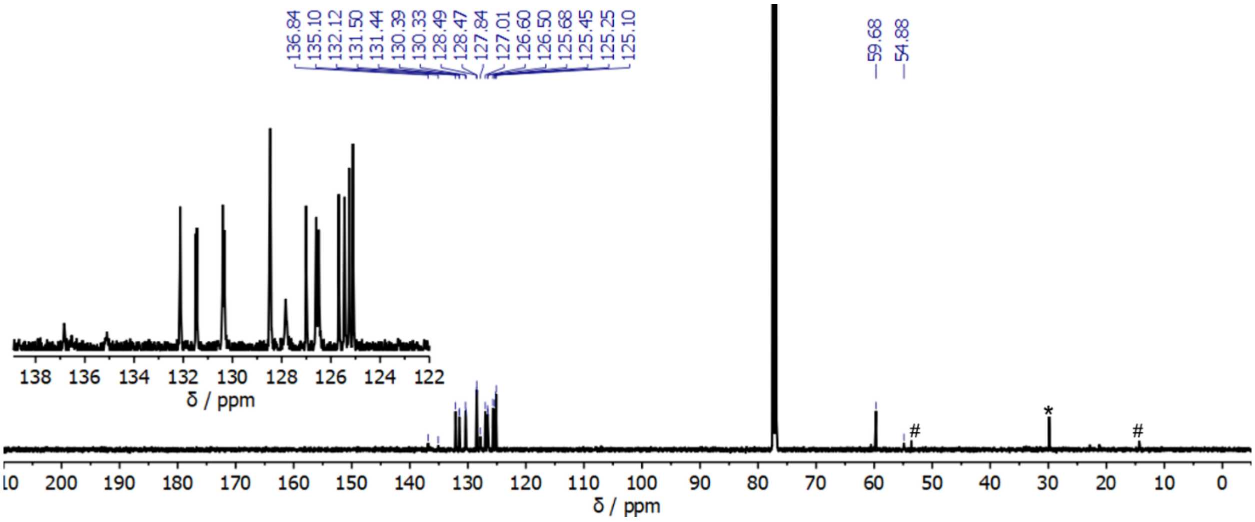

Figure S10. ^{13}C NMR (126 MHz, $CDCl_3$) spectrum of **1b**. * and # indicate residual grease and CH_2Cl_2 solvent, respectively.

Compound **2a**

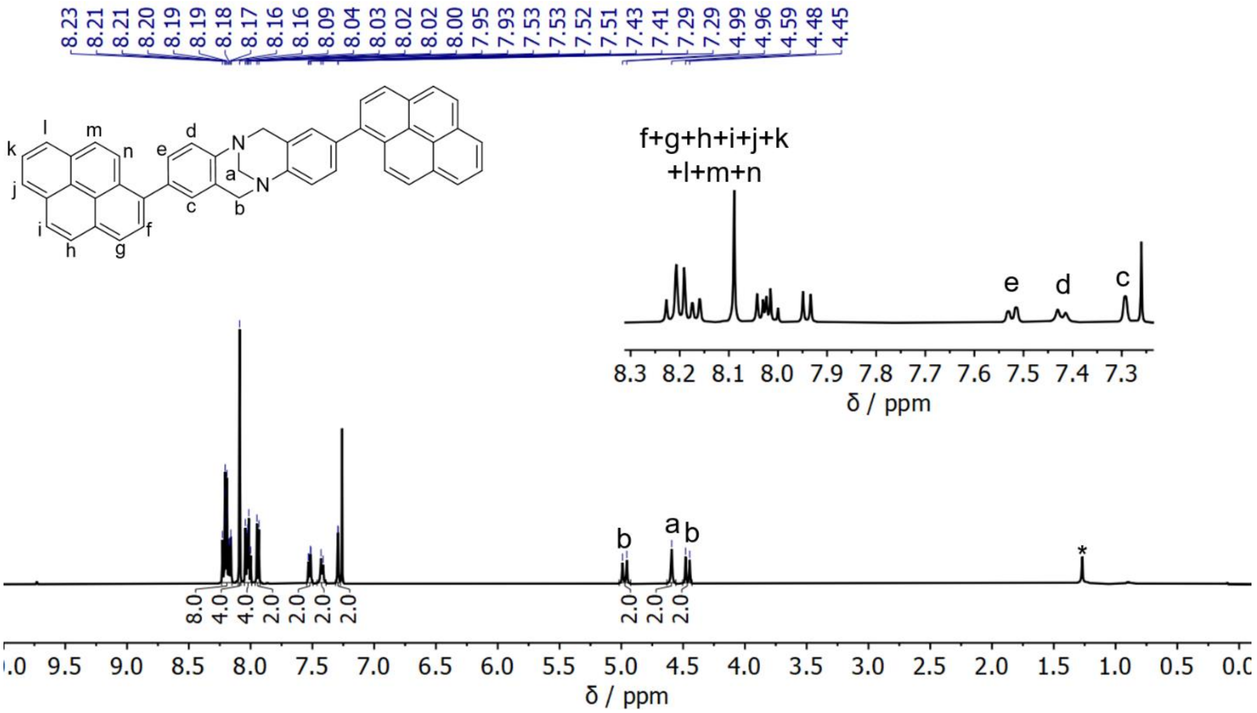

Figure S11. ^{1}H NMR (500 MHz, $CDCl_3$) spectrum of **2a**. * indicates residual grease.

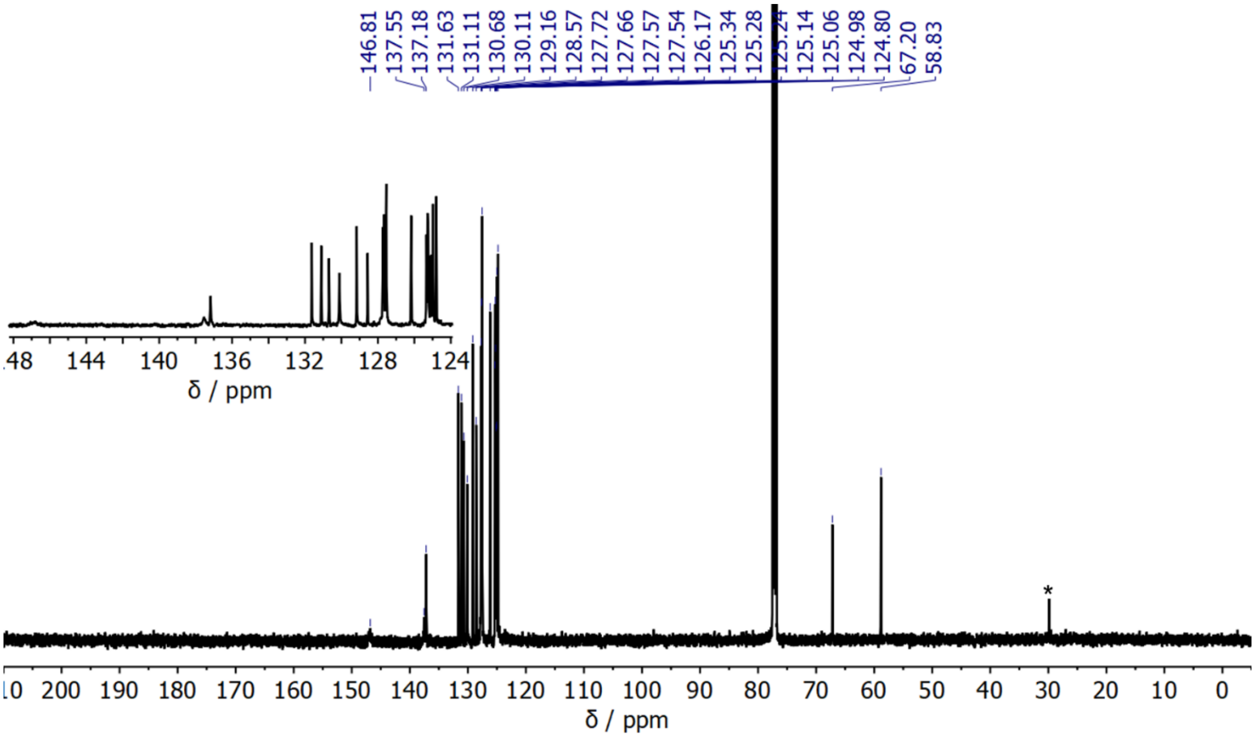

Figure S12. ^{13}C NMR (126 MHz, $CDCl_3$) spectrum of **2a**. * indicates residual grease.

Compound (*RR*)-**2a**

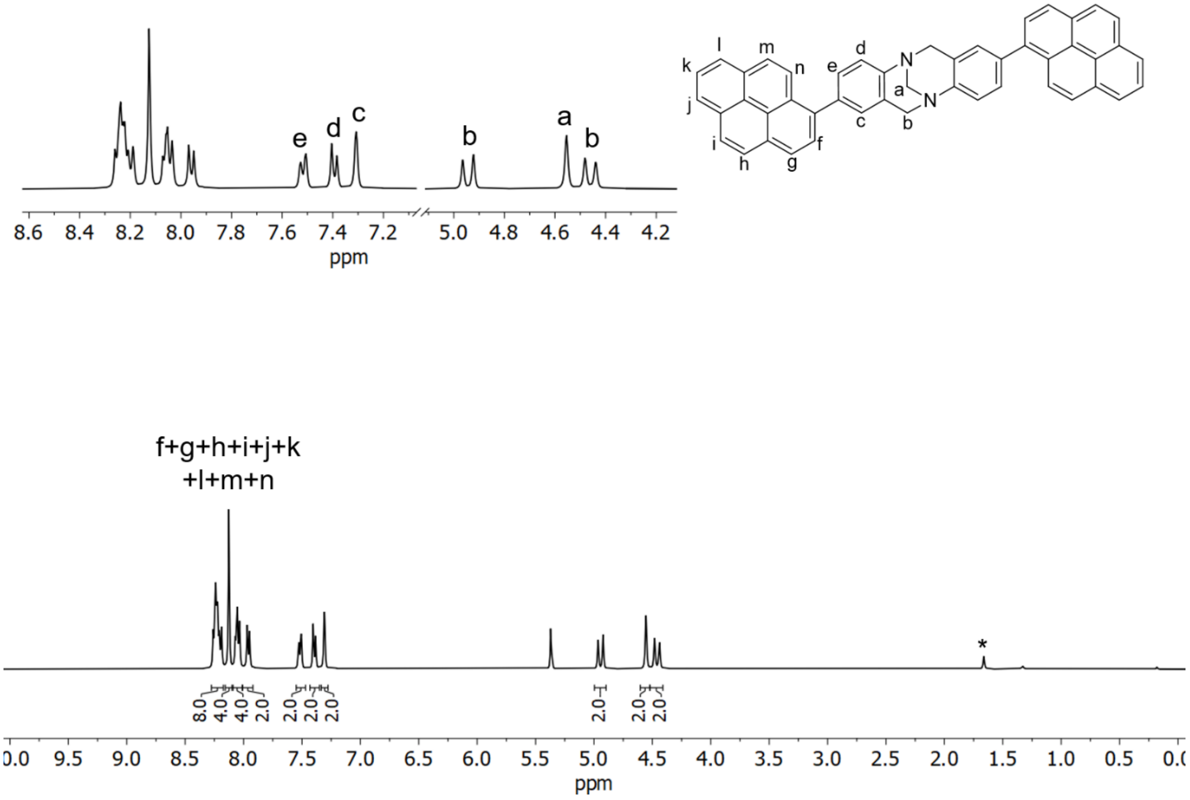

Figure S13. ^{1}H NMR (400 MHz, CD_2Cl_2) spectrum of **2a**. * indicates residual water. Spectral data agree with those reported for the racemic mixture.

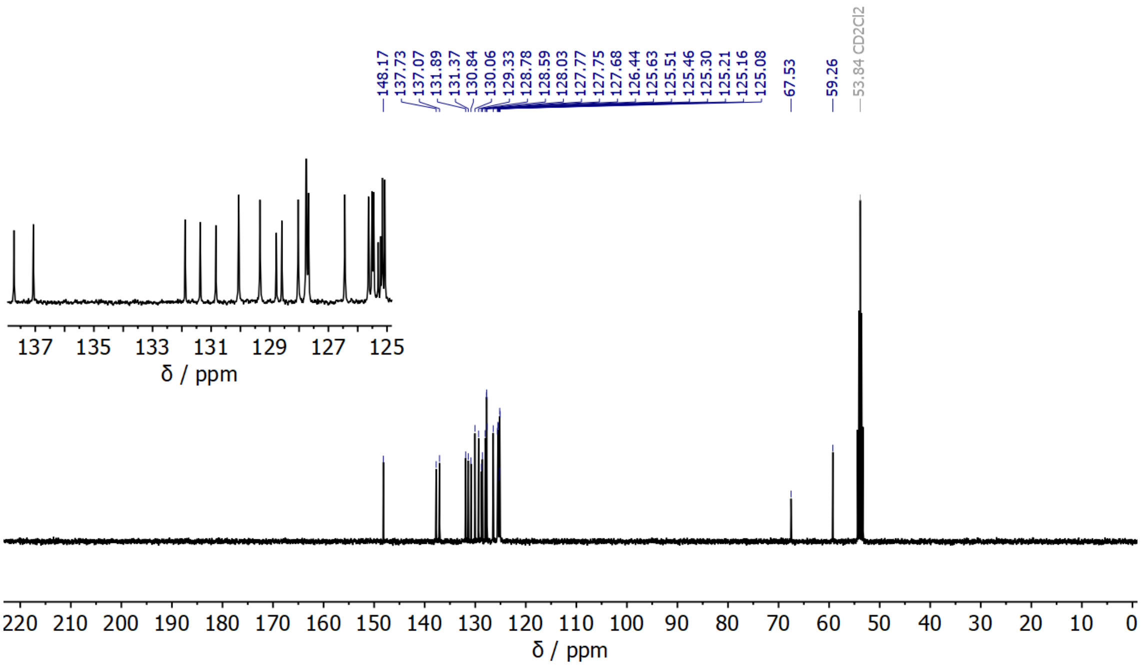

Figure S14. ^{13}C NMR (126 MHz, CD_2Cl_2) spectrum of **2a**.

Compound **2b**

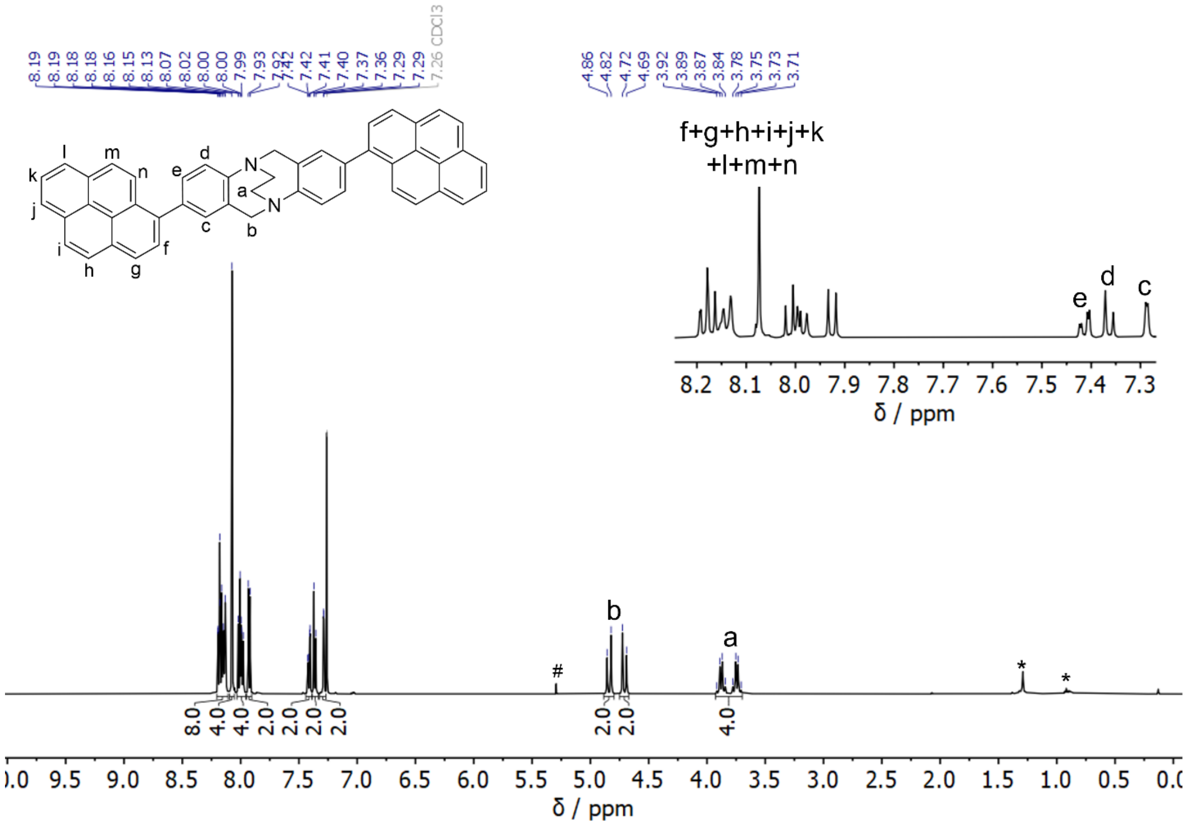

Figure S15. ^{1}H NMR (500 MHz, $CDCl_3$) spectrum of **2b**. * and # indicate residual grease and CH_2Cl_2, respectively.

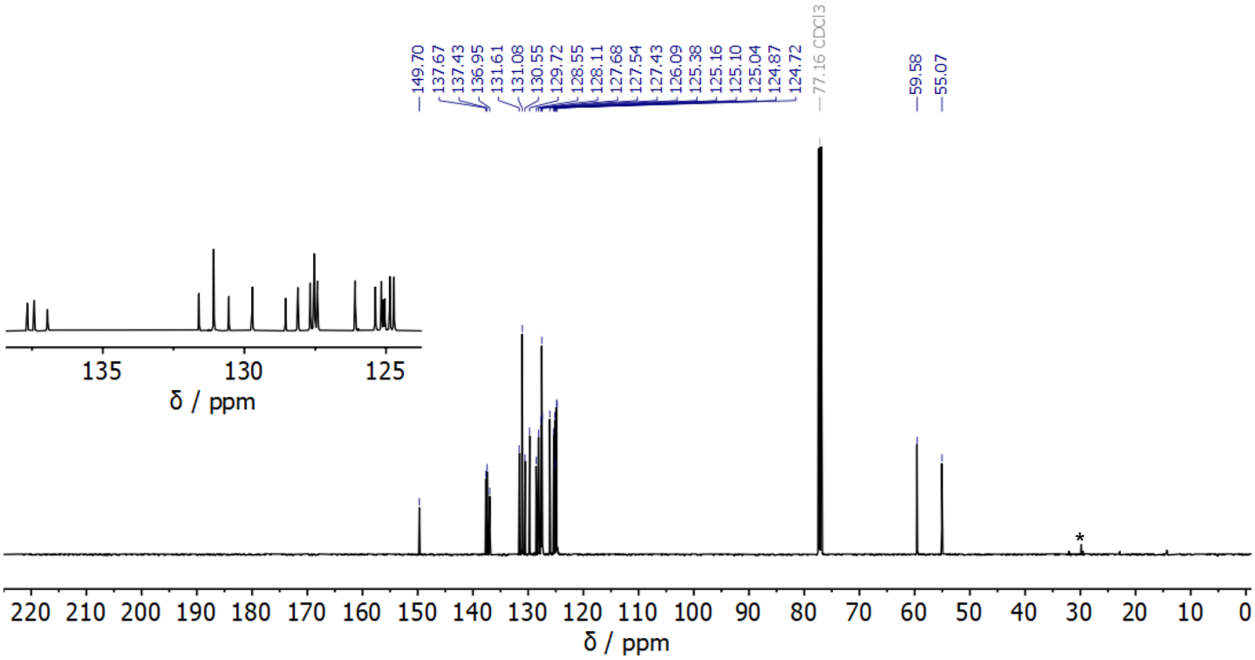

Figure S16. ^{13}C NMR (126 MHz, $CDCl_3$) spectrum of **2b**. * indicates residual grease.

Compound **3a**

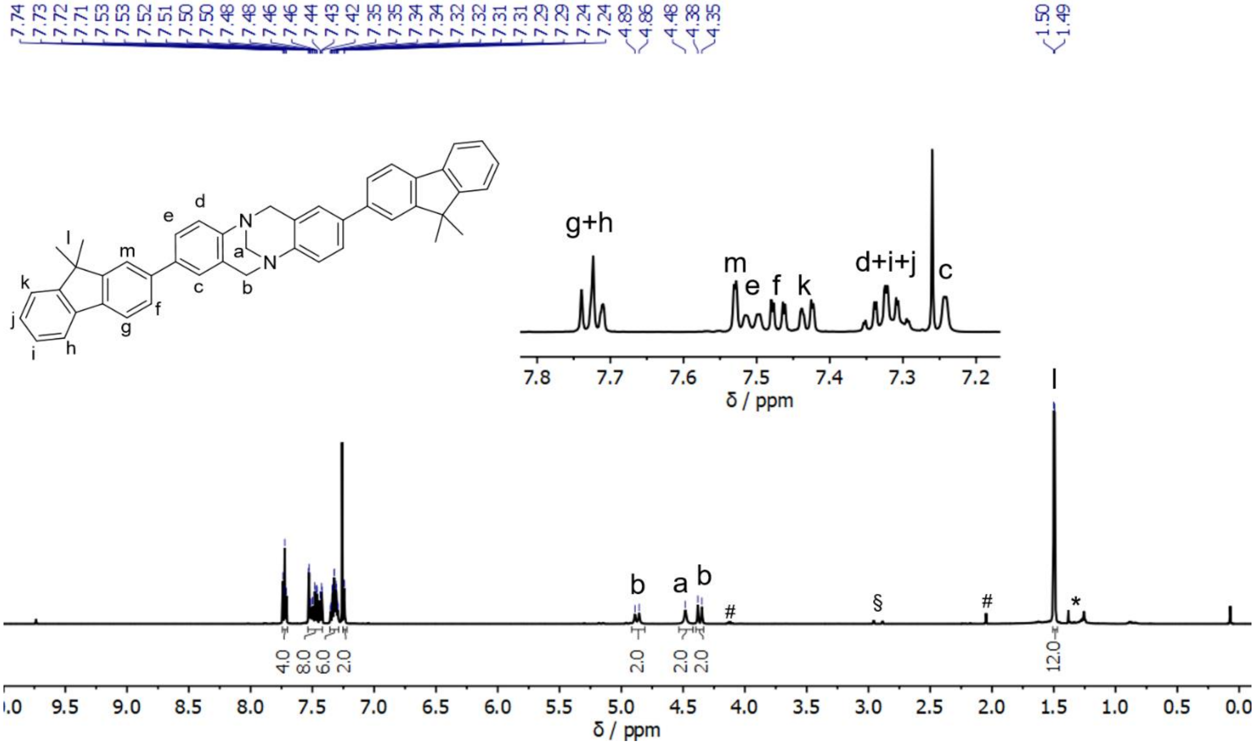

Figure S17. ^{1}H NMR (500 MHz, $CDCl_3$) spectrum of **3a**. *, # and § indicate residual grease, EtOAc and DMF solvent, respectively.

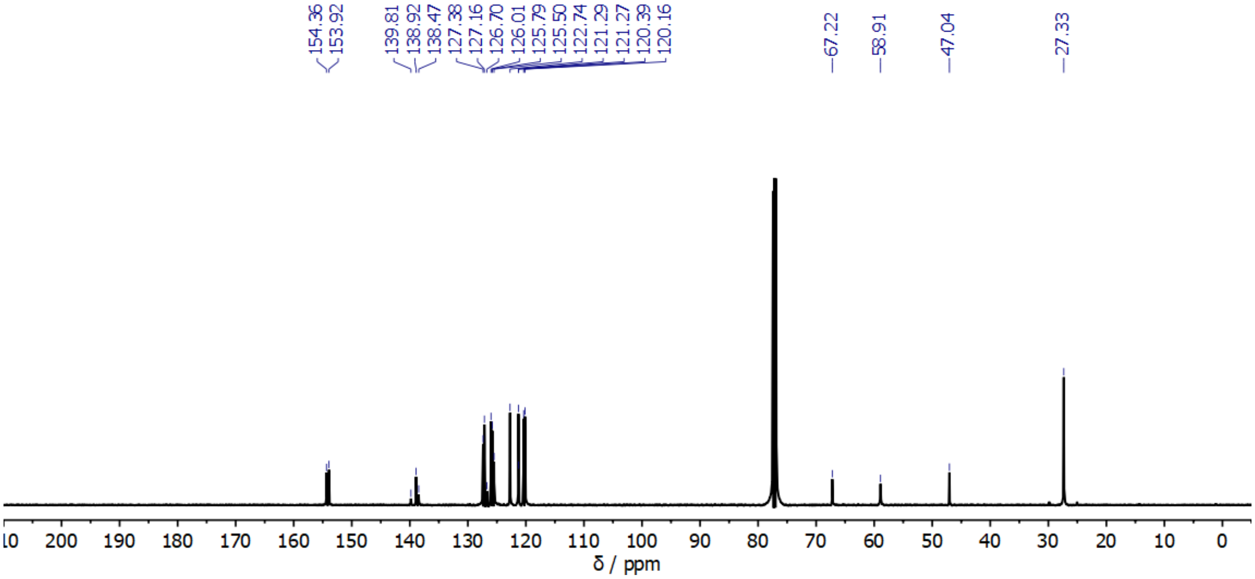

Figure S18. ^{13}C NMR (126 MHz, $CDCl_3$) spectrum of **3a**.

Compound **3b**

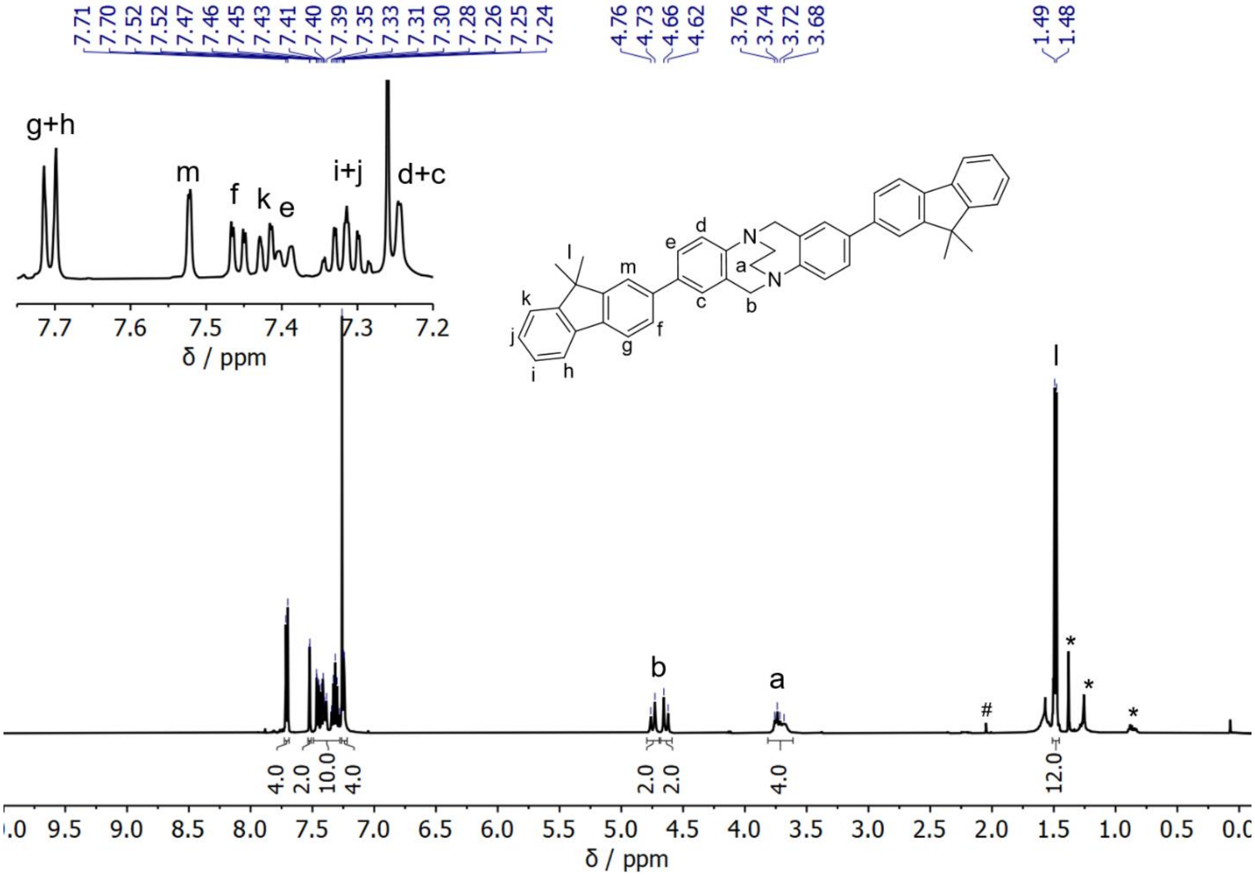

Figure S19. ^{1}H NMR (500 MHz, $CDCl_3$) spectrum of **3b**. * and # indicate residual grease, EtOAc solvent, respectively.

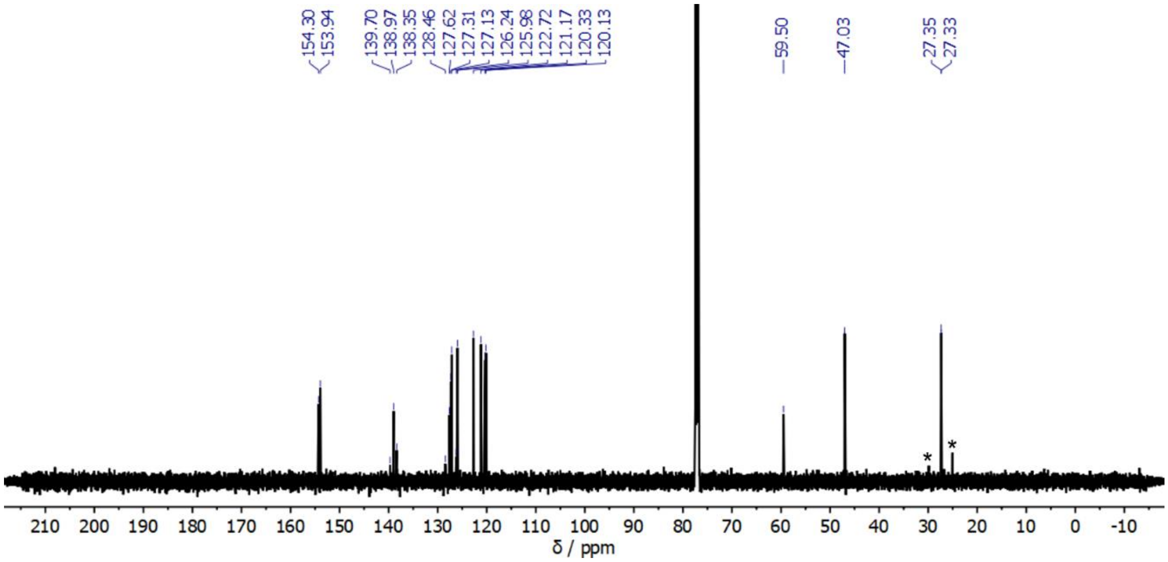

Figure S20. ^{13}C NMR (126 MHz, $CDCl_3$) spectrum of **3b**. * and # indicates residual grease.

4. Chiral HPLC Traces

4.1. Racemic resolution by CSP-HPLC

HPLC analyses were performed on an Agilent 1260 series equipped with the following modules: quaternary pump (G7111B 1260 Quat Pump), automatic sample injector (G2258A 1260 DL ALS), column thermostat (G1316A 1260 TCC), DAD detector (G7115A 1260 DADWR) and an automatic sample collector (G1364C 1260 FC-AS). Two chiral HPLC columns were employed for the separation of the compounds. CHIRALPAK® IC analytical column (4.6 × 250 mm) is packed with cellulose tris-(3,5-dichlorophenylcarbamate) immobilized on silica gel (5µm). CHIRALPAK® IA semipreparative column (10 × 250 mm) is packed with amylose tris-(3,5-dimethylphenylcarbamate) immobilized on silica gel (5 µm). In all cases the column temperature was set at 25°C and the flow was constant during operation. HPLC grade solvents were used for the separation of enantiomers. The injected solutions were prepared in CH_2Cl_2/hexane (8:2) at a concentration of 1 mg/mL.

Table S1. Summary of the retention time (t_r) of the two enantiomers of the six chiral compounds

Compound	t_r peak 1 / min	t_r peak 2 / min
**1a**	5.004	6.875
**1b**	9.919	11.674
**2a**	4.157	13.348
**2b**	3.950	12.633
**3a**	4.309	9.156
**3b**	3.921	8.714

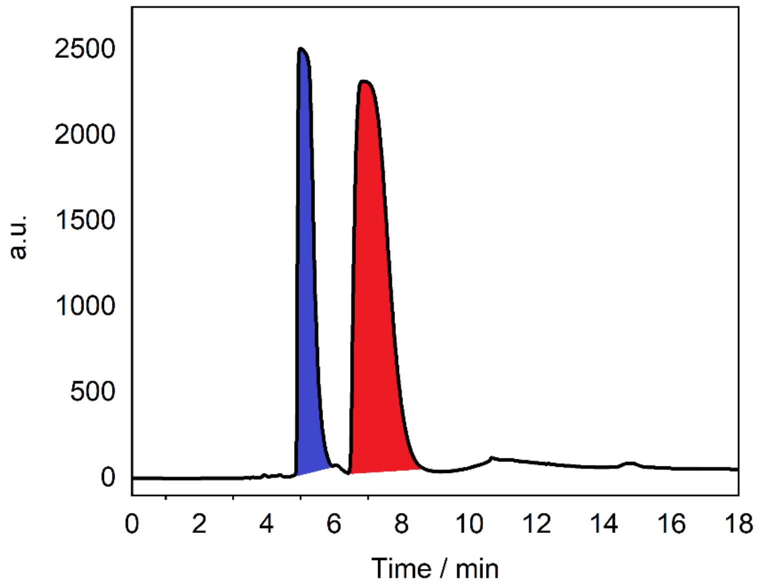

Figure S21. HPLC chromatogram (detection: λ = 254 nm) for the separation of the two enantiomers (blue and red peaks) of compound **1a** displaying a good separation

Table S2. Method used for the separation of compound **1a**

Column used	Flow / mL min^{-1}	Injection Volume / µL	
CHIRALPAK® IA	3.5	100	
Method			
Time / min	CH_2Cl_2	Hexane	2-Propanol
0	80	20	0
2	80	20	0
7	85	10	5
11	95	0	5
15	90	10	0
18	80	20	0

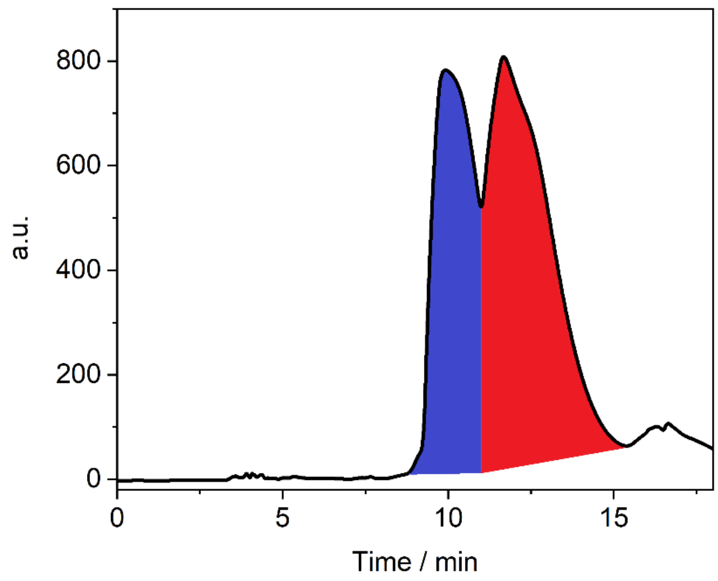

Figure S22. HPLC chromatogram (detection: λ = 254 nm) for the separation of the two enantiomers (blue and red peaks) of compound **1b** displaying a moderate separation. Only the very beginning of the first peak and the very end of the second peak were collected for the chiroptical investigation on this compound.

Table S3. Method used for the separation of compound **1b**

<table>
<tr><td colspan="4">Column used</td><td colspan="4">Flow / mL min^{-1}</td><td colspan="4">Injection Volume / µL</td></tr>
<tr><td colspan="4">CHIRALPAK® IA</td><td colspan="4">3.5</td><td colspan="4">50</td></tr>
<tr><td colspan="12">Method</td></tr>
<tr><td colspan="3">Time / min</td><td colspan="3">CH_2Cl_2</td><td colspan="3">Hexane</td><td colspan="3">2-Propanol</td></tr>
<tr><td colspan="3">0</td><td colspan="3">80</td><td colspan="3">20</td><td colspan="3">0</td></tr>
<tr><td colspan="3">4</td><td colspan="3">80</td><td colspan="3">20</td><td colspan="3">0</td></tr>
<tr><td colspan="3">8</td><td colspan="3">85</td><td colspan="3">15</td><td colspan="3">0</td></tr>
<tr><td colspan="3">12</td><td colspan="3">85</td><td colspan="3">10</td><td colspan="3">5</td></tr>
<tr><td colspan="3">15</td><td colspan="3">90</td><td colspan="3">10</td><td colspan="3">0</td></tr>
<tr><td colspan="3">19</td><td colspan="3">80</td><td colspan="3">20</td><td colspan="3">0</td></tr>
</table>

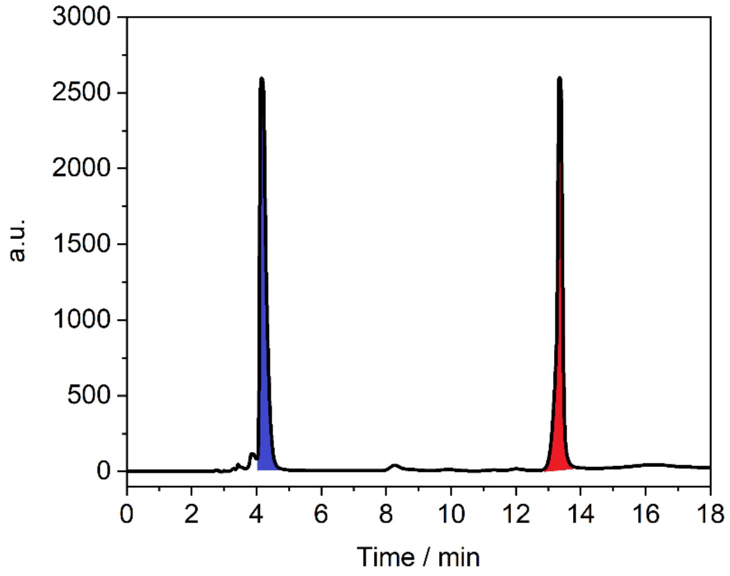

Figure S23. HPLC chromatogram (detection: λ = 350 nm) for the separation of the two enantiomers (blue and red peaks) of compound **2a** displaying an excellent separation

Table S4. Method used for the separation of compound **2a**

Column used	Flow / mL min^{-1}	Injection Volume / µL	
CHIRALPAK® IC	1	50	
Method			
Time / min	CH_2Cl_2	Hexane	2-Propanol
0	80	20	0
2	80	20	0
7	80	15	5
11	85	10	5
15	90	10	0
18	90	10	0

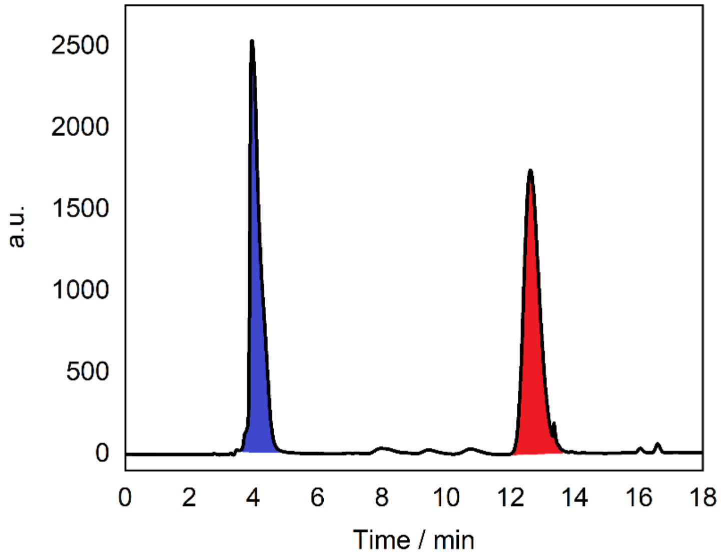

Figure S24. HPLC chromatogram (detection: λ = 350 nm) for the separation of the two enantiomers (blue and red peaks) of compound **2b** displaying an excellent separation

Table S5. Method used for the separation of compound **2b**

Column used	Flow / mL min^{-1}	Injection Volume / µL	
CHIRALPAK® IC	1	50	
Method			
Time / min	CH_2Cl_2	Hexane	2-Propanol
0	80	20	0
2	80	20	0
7	80	15	5
11	85	10	5
15	90	10	0
18	90	10	0

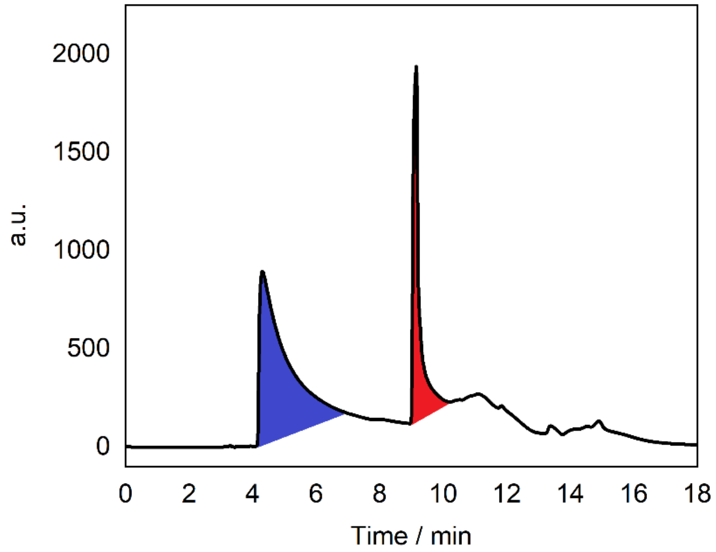

Figure S25. HPLC chromatogram (detection: λ = 300 nm) for the separation of the two enantiomers (blue and red peaks) of compound **3a** displaying a good separation

Table S6. Method used for the separation of compound **3a**

Column used	Flow / mL min^{-1}	Injection Volume / µL	
CHIRALPAK® IC	1	50	
Method			
Time / min	CH_2Cl_2	Hexane	2-Propanol
0	80	20	0
3	80	20	0
7	80	15	5
11	85	10	5
15	90	10	0
18	90	10	0

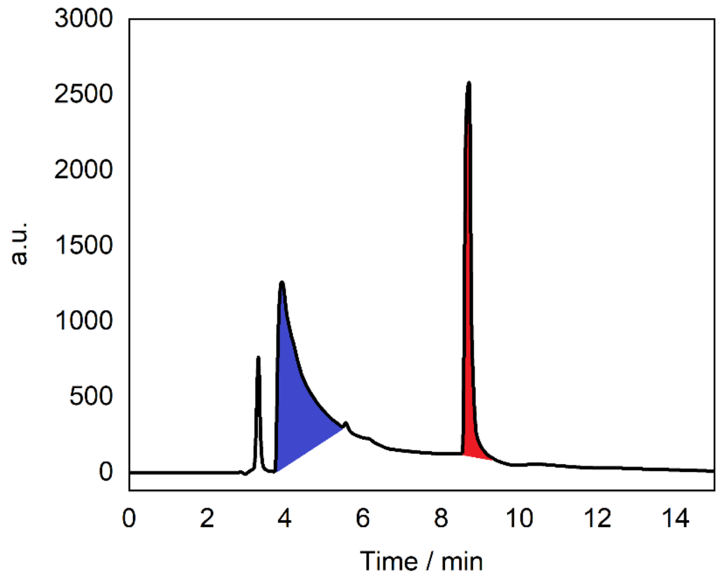

Figure S26. HPLC chromatogram (detection: λ = 300 nm) for the separation of the two enantiomers (blue and red peaks) of compound **3b** displaying an excellent separation

Table S7. Method used for the separation of compound **3b**

Column used	Flow / mL min^{-1}	Injection Volume / µL	
CHIRALPAK® IC	1	50	
Method			
Time / min	CH_2Cl_2	Hexane	2-Propanol
0	80	20	0
3	80	20	0
4	80	15	5
7	85	10	5
12	85	10	5
15	80	20	0

4.2. Determination of enantiomeric excess (*ee*) of (*RR*)-5 and (*RR*)-2a

The ee of (*RR*)-**5** obtained by racemic resolution by co-crystallization with *O,O'*-dibenzoyl-L-tartaric acid followed by extraction with $Na_2CO_{3(aq)}$ (5%) and (*RR*)-**2a** obtained from (*RR*)-**5** were determined by Chiral Stationary Phase HPLC (CSP-HPLC) analyses performed on the Agilent 1260 series used in section 4.1. using a CHIRALPAK® IC analytical column (4.6 × 250 mm). The column temperature was set at 20 °C and the flow was constant during operation. HPLC grade solvents were used in the analysis. The injected solutions were prepared in CH_2Cl_2/hexane (1:1) ((*RR*)-**5**) or CH_2Cl_2 ((*RR*)-**2a**). *Rac*-**5** and *rac*-**2a** were also analyzed as references using the same method as (*RR*)-**5** or (*RR*)-**2a**, respectively. All samples were prepared at a concentration of *ca.* 1 mg/mL

(*RR*)-**5**

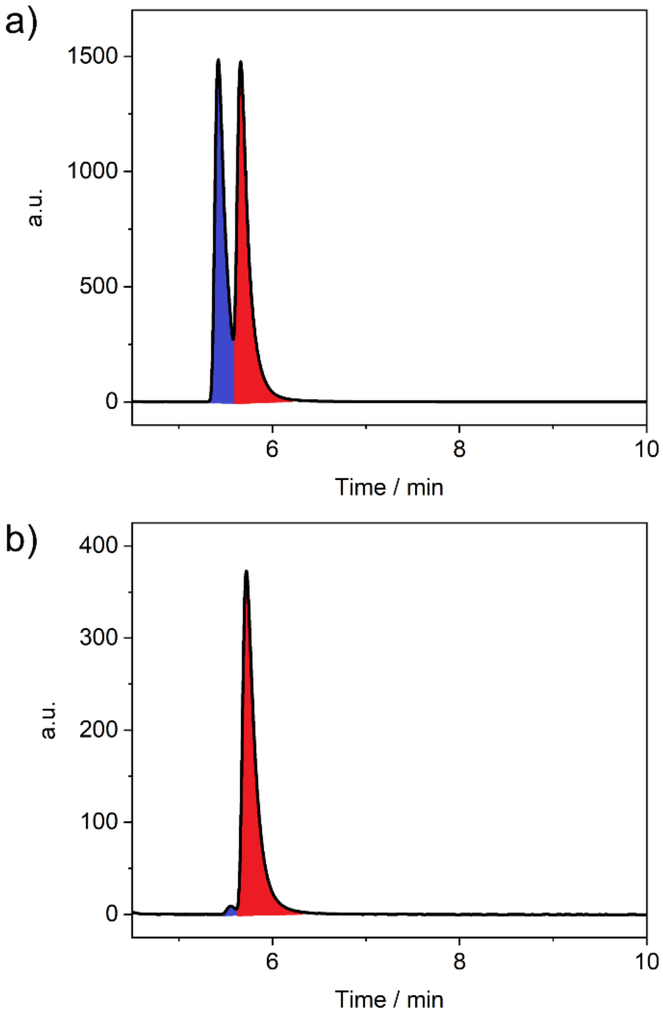

Figure S27. CSP-HPLC (Chiralpak® IC) chromatograms (detection: λ = 254 nm) of: a) *rac*-**5**; b) (*RR*)-**5**.

Table S8. Method used for the analysis of compounds *rac*-**5** and (*RR*)-**5**

Column used	Flow / mL min^{-1}	Injection Volume / µL
CHIRALPAK® IC	0.8	35
Method		
Time / min	CH_2Cl_2	Hexane
0	50	50
6	50	50
10	100	0
12	100	0

Table S9. Data for the analysis of reference *rac*-**5** [a]

Peak	time/min	Area %
1	5.421	46.4
2	5.660	53.6

[a] Detection: λ = 254 nm

Table S10. Data for the quantification of the enantiomeric excess of (*RR*)-**5**[a]

Peak	time/min	Area %
1	5.555	1.44
2	5.721	98.56

[a] Detection: λ = 254 nm

(*RR*)-**2a**

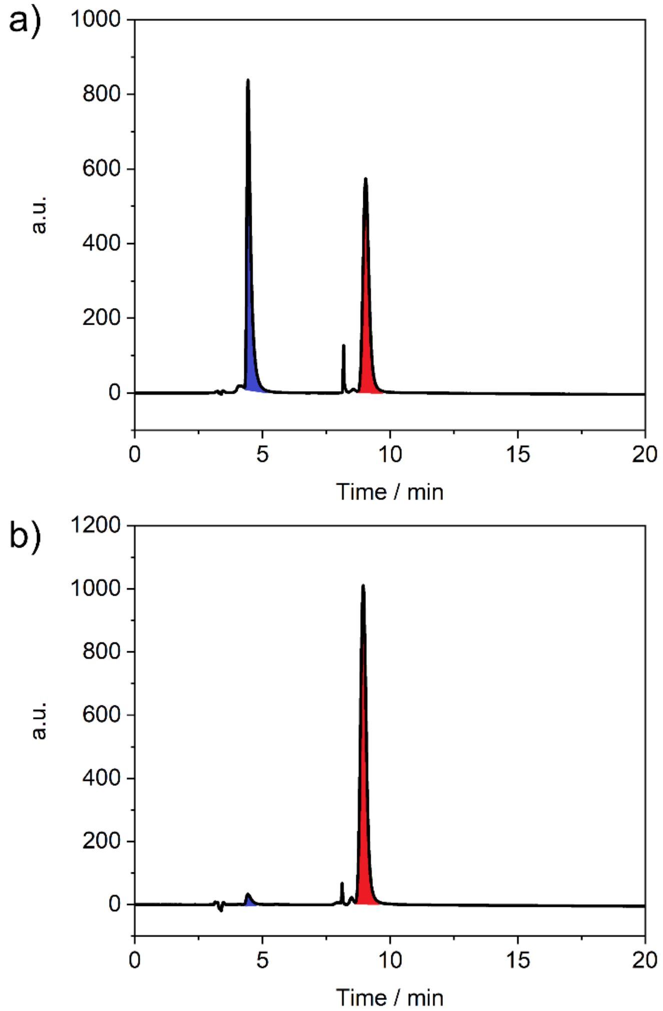

Figure S28. CSP-HPLC (Chiralpak® IC) chromatograms (detection: λ = 325 nm) of: a) *rac*-**2a**; b) (*RR*)-**2a**.

Table S11. Method used for the analysis of compounds *rac*-**2a** and (*RR*)**-2a**

Column used		Flow / mL min^{-1}		Injection Volume / µL
CHIRALPAK® IC		1.0		50
Method				
Time / min	CH_2Cl_2		Hexane	2-Propanol
0	80		20	0
2	80		20	0
7	80		15	5
11	85		10	5
15	90		10	0
18	90		10	0

Table S12. Data for the analysis of reference *rac*-**2a** [a]

Peak	time/min	Area %
1	4.435	49.5
2	9.046	50.5

[a] Detection: λ = 325 nm

Table S13. Data for the quantification of the enantiomeric excess of (*RR*)-**2a**[a]

Peak	time/min	Area %
1	4.430	2.7
2	8.949	97.3

[a] Detection: λ = 325 nm

5. Photophysical Properties

The spectra were recorded at *ca*. 6 × 10^{-5} M (for **1a-b**), 2 × 10^{-5} M (for **2a-b**, **3a-b**, (*RR*)-**2a** obtained by the diastereoisomeric resolution of (*RR*)-**5** followed by Suzuki coupling, and **2b** in the presence of 100 equiv. of CF_3CO_2H) and 3 × 10^{-5} M (for (*RR*)-**5**) in HPLC grade CH_2Cl_2 at 25 ºC. For ECD measurements, a fixed slit-width of 2 mm and 0.1 s of integration time were selected. For CPL measurements, a fixed slit-width of 2 mm (for **1a-b**) or 1.5 mm (for **2a-b**, **3a-b** and **2b** in the presence of 100 equiv. of CF_3CO_2H), and 0.5 s of integration time were selected. CPL and ECD spectra of compounds **1a,b-3a,b** and **2b** in the presence of 100 equiv. of CF_3CO_2H correspond to an average spectrum calculated after 250 and 50 scans, respectively. ECD spectra of compounds (*RR*)-**5a** and (*RR*)-**2a**, obtained by the diastereoisomeric resolution of (*RR*)-**5a** followed by Suzuki coupling, correspond to an average spectrum calculated after 75 scans.

Time-resolved fluorescence decay traces were collected via the time-correlated single photon counting (TCSPC) method using a FluoTime 200 fluorometer (PicoQuant, GmbH). The excitation source employed to the lifetime analysis of compounds **1a-b** and **2a-b** was a 375 nm laser and a 320 nm LED for compounds **3a-b**, using in all the cases a pulse repetition rate of 20 MHz. To reconstruct the whole emission spectra, for the time-resolved emission spectroscopy (TRES) of compound **2b** a 320 nm LED was employed, and 41 fluorescence decay traces were collected in the 350-550 nm emission range ($\Delta\lambda_{em}$ = 5 nm).

The fluorescence decay traces were fitted to a mono- to three-exponential function using iterative deconvolution methods built-in the FluoFit software (PicoQuant). For each sample, the decay traces were fitted globally with the decay times linked as shared parameters, whereas the pre-exponential factors were local adjustable parameters. The quality of fittings was assessed by the value of the reduced chi-squared, χ^2, parameter and random distributions of the weighted residuals and the autocorrelation functions. The species-associated emission spectra of each species *i* at any given emission wavelength ($SAEMS_i(\lambda_{em})$) is given by the fluorescence intensity emitted by the species *i* ($A_{i,\lambda em} \times \tau_i$), normalized by the total intensity and corrected for the different detection sensitivity using the total intensity of the steady-state spectrum ($I_{ss,\lambda em}$), as it is shown in Eq. (1):

$$SAEMS_i(\lambda_{em}) = \frac{A_{i,\lambda em} \times \tau_i}{\sum_i A_{i,\lambda em} \times \tau_i} \cdot I_{ss,\lambda em} \qquad \text{(Eq. 1)}$$

The approximate contribution of each species can be assessed as the area under the SAEMS. This estimation assumes equal excitation rate for all the species, as the initial amount of each form in the excited state (after the pulse excitation) is unknown.

For the calculation of the molar extinction coefficient, solutions of each compound in HPLC grade CH_2Cl_2 were prepared using volumetric flasks and the absorbance spectra were recorded in the 250-500 nm range.

The molar extinction coefficient at each wavelength was obtained from the slope of the least-squares fitting of the absorbance (A) vs. concentration data.

Quantum yields were determined by measuring both absorbance and fluorescence of compounds **1a,b-3a,b** in CH_2Cl_2, and **1b-3b** in CH_2Cl_2 in the presence of 50 equivalents of CF_3CO_2H, using quinine sulfate in 0.1 M H_2SO_4 as standard ($\Phi_r = 0.54$).[S4] For the relative determination of the fluorescence quantum yield Φ in a series of solvents, Eq. 2 was used.[S5,S6]

$$\Phi_x = \Phi_r \times \frac{F_x}{F_r} \times \frac{1-10^{-Ar(\lambda ex)}}{1-10^{-Ax(\lambda ex)}} \times \frac{n_x^2}{n_r^2} \qquad \text{(Eq. 2)}$$

The subscripts *x* and *r* refer respectively to the sample and a reference (standard) fluorophore with known quantum yield Φ_r in a specific solvent; *F* stands for the spectrally corrected, integrated fluorescence spectra; $A(\lambda_{ex})$ denotes the absorbance at the used excitation wavelength λ_{ex}; and *n* represents the refractive index of the solvent (in principle at the average emission wavelength). To minimize inner filter effects, the absorbance at the excitation wavelength λ_{ex} was kept under 0.1. The measurements were performed using 10×10 mm cuvettes.

The titrations of compounds **1b-3b** were performed by stepwise addition of increasing quantities (from 1 to 50 equivalents) of a CF_3CO_2H solution (25 mM) to solutions of compounds **1b** (7 μM), **2b** (2.5 μM) and **3b** (2 μM) in CH_2Cl_2. Emission spectra were recorded after excitation at 340 nm for compounds **1b-2b** and 320 nm for compound **3b**.

The *in situ* CPL switching was carried out by repeatedly adding CF_3CO_2H and Et_3N to a solution of (*RR*)-**2b** in CH_2Cl_2. After the first measurement, i.e., when the solution was neutral (cycle 0), 100 equiv. of CF_3CO_2H (cycle 0.5) were added, followed 110 equiv. of Et_3N (cycle 1), 140 equiv. of CF_3CO_2H (cycle 1.5), and 180 equiv. of Et_3N (cycle 2). For each cycle, fluorescence and CPL spectra were recorded on an Olis DSM172 spectrophotometer. For these measurements, a fixed slit-width of 1.5 mm, and 0.1 s (fluorescence) or 0.5 s (CPL) of integration time were selected. The CPL spectra correspond to an average spectrum calculated after 200 scans.

Table S14. Summary of the optical properties of the six chiral compounds in CH_2Cl_2

Compound	λ_{abs} / nm	ε / M^{-1} cm^{-1}	λ_{em} / nm	Φ_F	τ_1 / ns	τ_2 / ns	$\tau_{average}$ / ns
**1a**	368	1.7×10^4	424	0.477±0.039	4.589±0.016	5.726±0.068	4.77
**1b**	368	1.8×10^4	427	0.543±0.035	5.443±0.014	N/A	N/A
**2a**	347	6.6×10^4	415	0.660±0.033	4.505±0.014	2.183±0.089	4.27
**2b**	347	5.7×10^4	431	0.613±0.032	3.05±0.01	N/A	N/A
**3a**	319	5.8×10^4	382	0.762±0.039	1.2847±0.0045	N/A	N/A
**3b**	319	5.0×10^4	394	0.757±0.031	1.911±0.006	N/A	N/A

Table S15. Summary of the chiroptical properties of the six chiral compounds in CH_2Cl_2

Compound	$\|g_{abs}\|^a$	$\|g_{lum}\|$	B_{CPL}^b / L mol^{-1} cm^{-1}
**1a**	4×10^{-4}	2.5×10^{-4}	1.02
**1b**	7×10^{-4}	4.6×10^{-4}	2.24
**2a**	3×10^{-4}	5.8×10^{-4}	12.6
**2b**	3×10^{-4}	5.3×10^{-4}	9.21
**3a**	9×10^{-4}	1.2×10^{-3}	26.3
**3b**	6×10^{-4}	6.5×10^{-4}	12.3

[a]The $|g_{abs}|$ values given in this Table correspond to the dimensionless dissymmetry factor values at the maximum of the lowest energy transitions.
[b]Calculated at λ_{exc} = 368 nm (for **1a-b**); 347 nm (for **2a-b**); 319 nm (for **3a-b**)

Table S16. Summary of the photophysical properties of **2b** in CH_2Cl_2 in the presence of an excess of CF_3CO_2H

λ_{abs} / nm	ε / M^{-1} cm^{-1}	λ_{em} / nm	Φ_F	τ_1 / ns	τ_2 / ns	τ_3 / ns	$\|g_{abs}\|^a$	$\|g_{lum}\|$	B_{CPL}^b / L mol^{-1} cm^{-1}
343	5.6×10^4	397	0.447±0.008	0.7	3.0	14.5	4×10^{-4}	3×10^{-4}	3.72

[a]The $|g_{abs}|$ values given in this Table correspond to the dimensionless dissymmetry factor values of the lowest energy transitions.
[b]Calculated at λ_{exc} = 343 nm

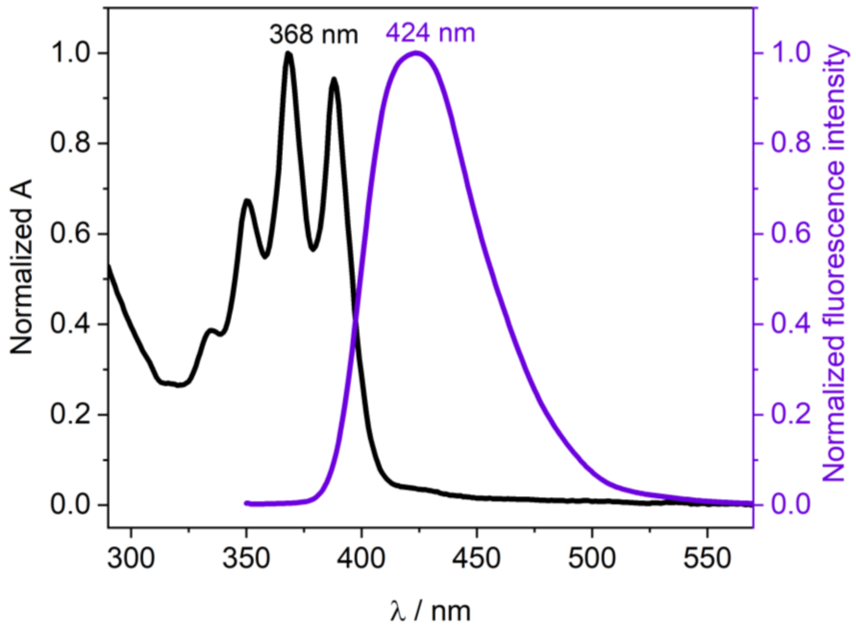

Figure S29. Normalized absorption (black line) and emission (purple line; λ_{exc} = 340 nm) spectra of **1a** in CH_2Cl_2

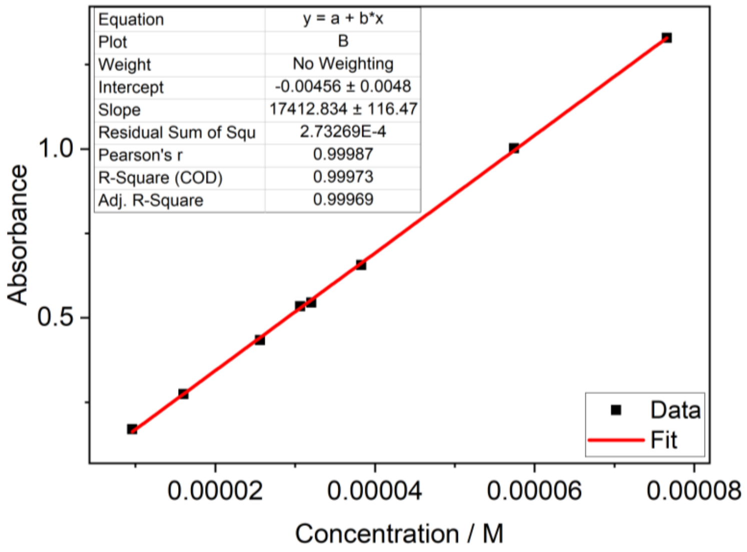

Figure S30. Plot of the absorbance at the absorption λ_{max} of **1a** versus concentration. The molar extinction coefficient (ε) is 1.7×10^4 L mol^{-1} cm^{-1} at 368 nm in CH_2Cl_2

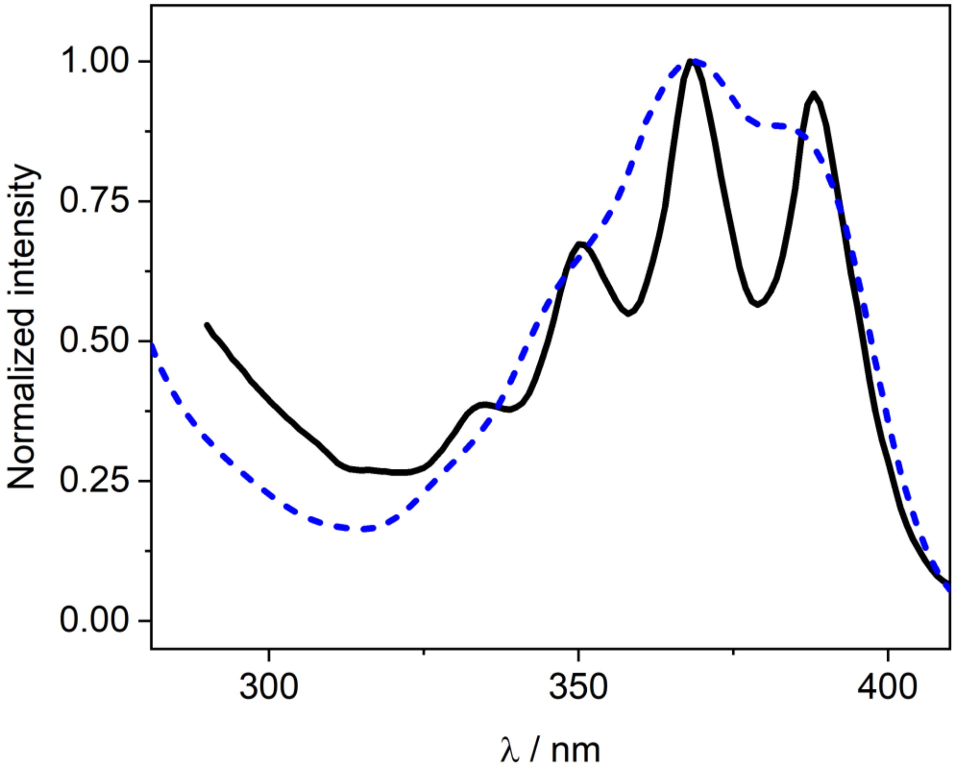

Figure S31. Comparison of the normalized absorption (black line) and excitation (blue dashed line; λ_{em} = 425 nm) spectra of **1a** in CH_2Cl_2

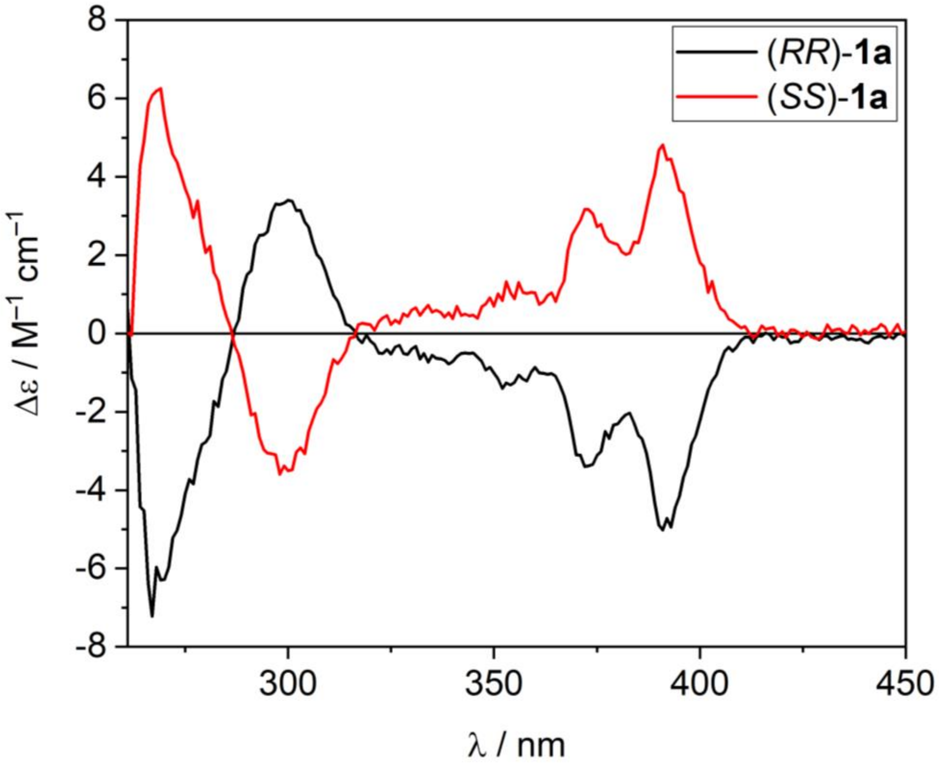

Figure S32. ECD spectra of (*RR*)/(*SS*)-**1a** in CH_2Cl_2

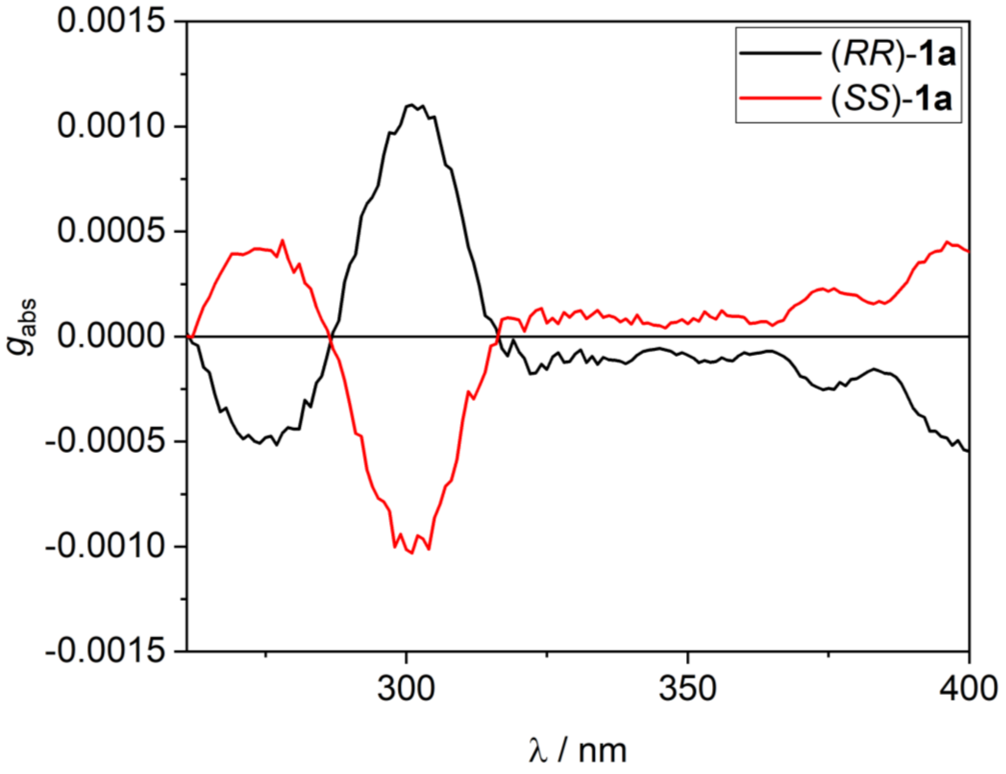

Figure S33. g_{abs} spectra of (*RR*)/(*SS*)-**1a** in CH_2Cl_2 (lowest energy transition $|g_{abs}| = 4 \times 10^{-4}$)

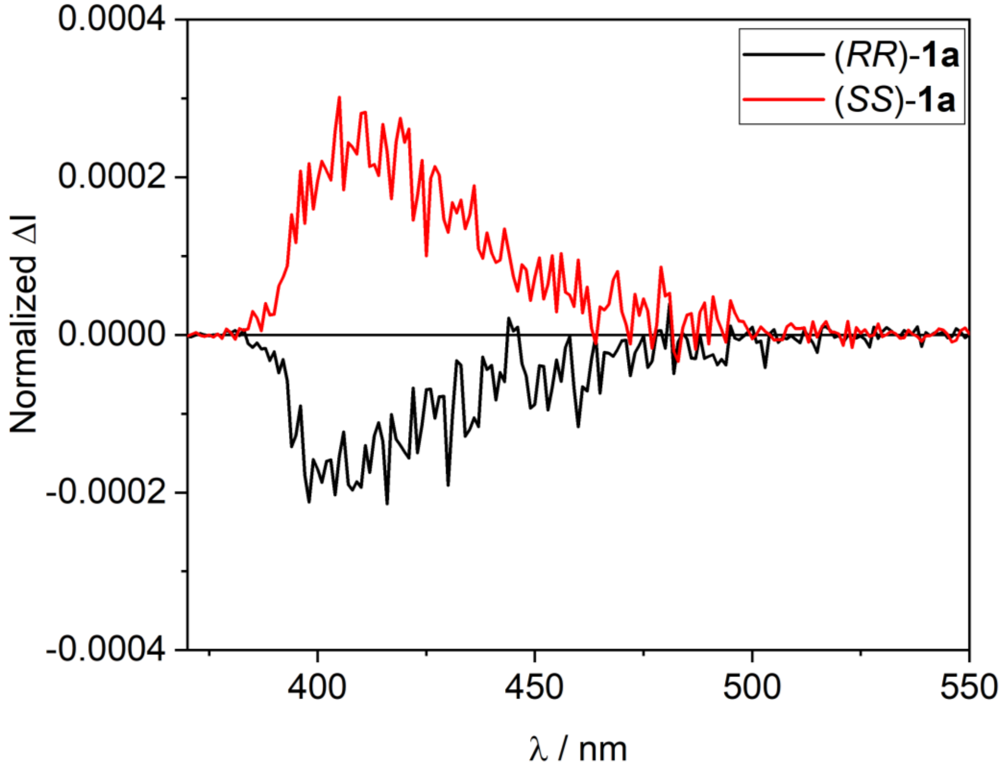

Figure S34. CPL (λ_{exc} = 403 nm) spectra of (*RR*)/(*SS*)-**1a** in CH_2Cl_2

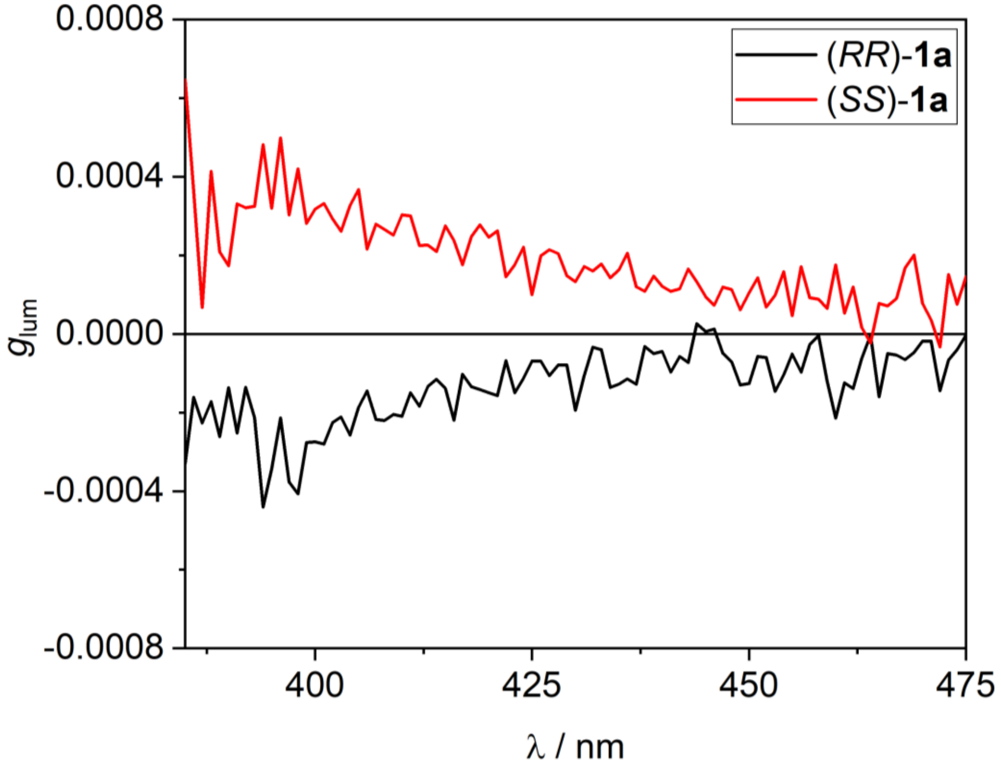

Figure S35. g_{lum} (λ_{exc} = 403 nm) spectra of (*RR*)/(*SS*)-**1a** in CH_2Cl_2 ($|g_{lum}|$ = 2.5 × 10^{-4})

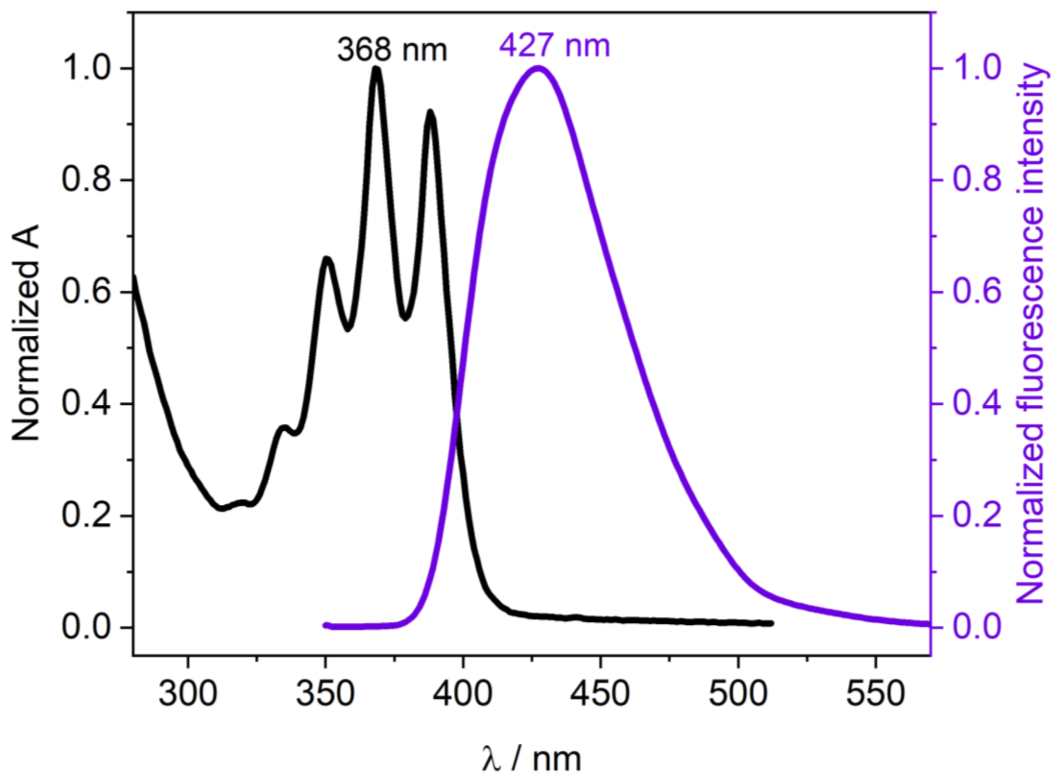

Figure S36. Normalized absorption (black line) and emission (purple line; λ_{exc} = 340 nm) spectra of **1b** in CH_2Cl_2

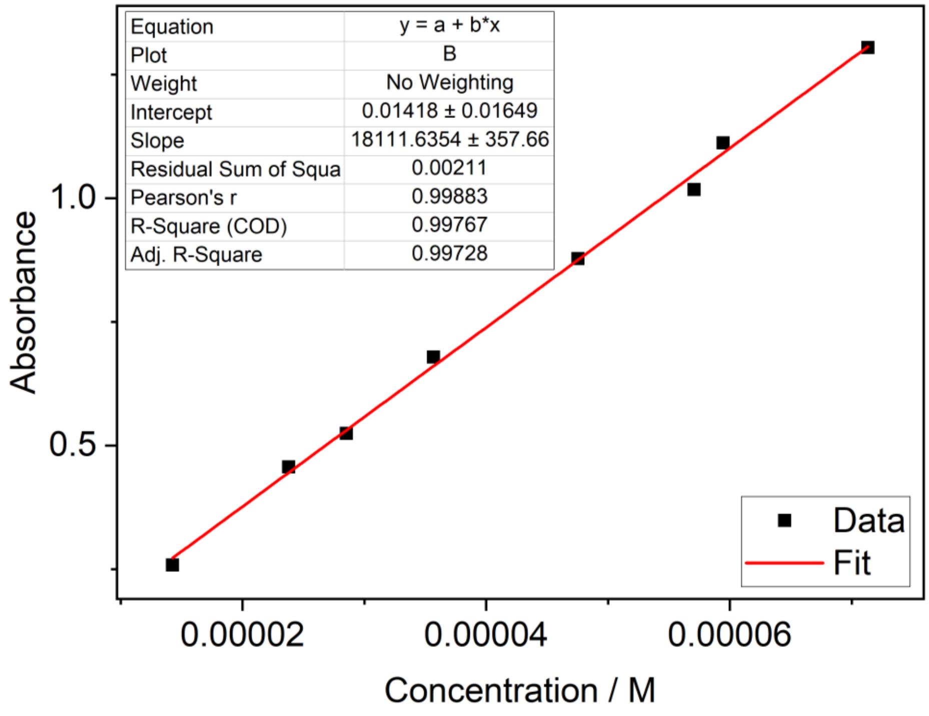

Figure S37. Plot of the absorbance at the absorption λ_{max} of **1b** versus concentration. The molar extinction coefficient (ε) is 1.8×10^4 L mol^{-1} cm^{-1} at 368 nm in CH_2Cl_2

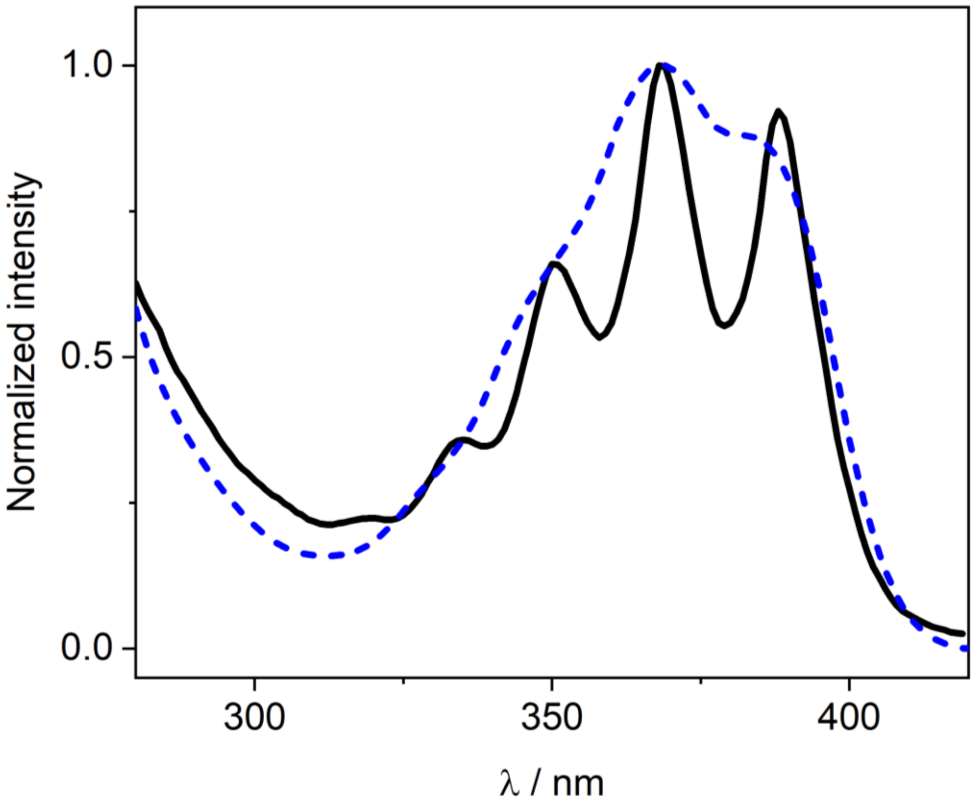

Figure S38. Comparison of the normalized absorption (black line) and excitation (blue dashed line; λ_{em} = 430 nm) spectra of **1b** in CH_2Cl_2

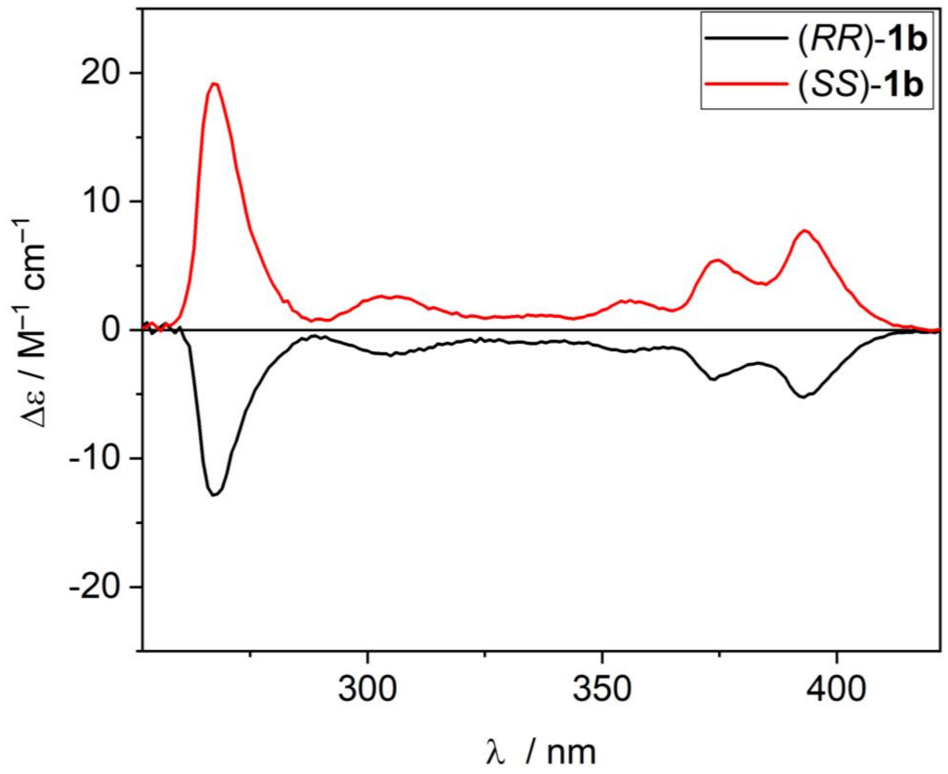

Figure S39. ECD spectra of (*RR*)/(*SS*)-**1b** in CH_2Cl_2

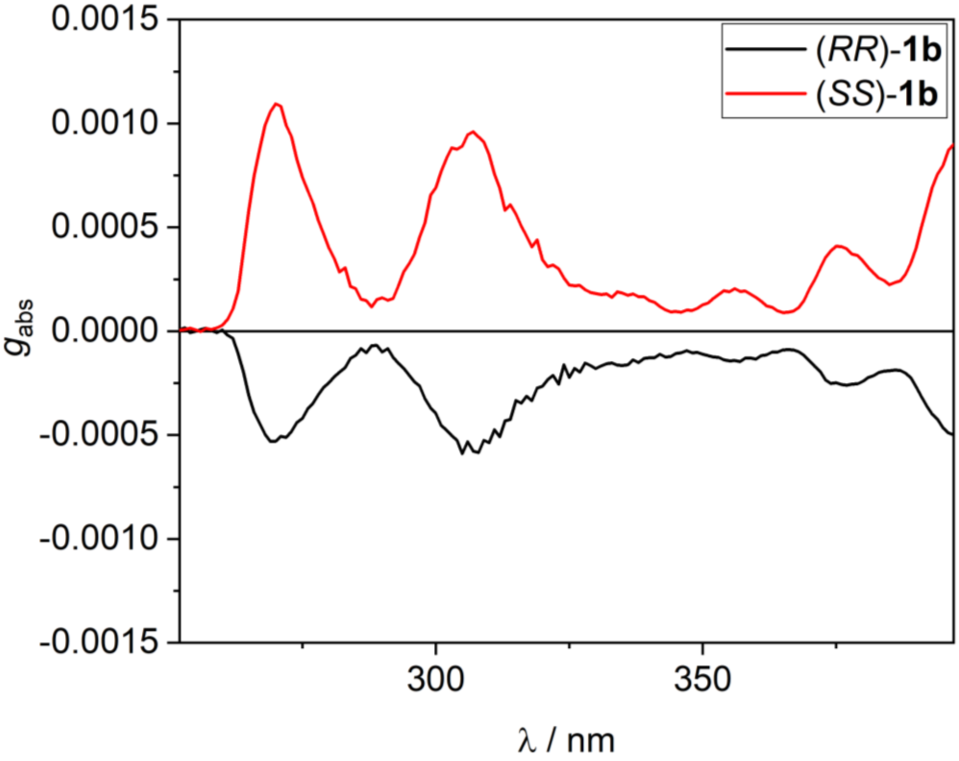

Figure S40. g_{abs} spectra of (*RR*)/(*SS*)-**1b** in CH_2Cl_2 (lowest energy transition $|g_{abs}| = 7 \times 10^{-4}$)

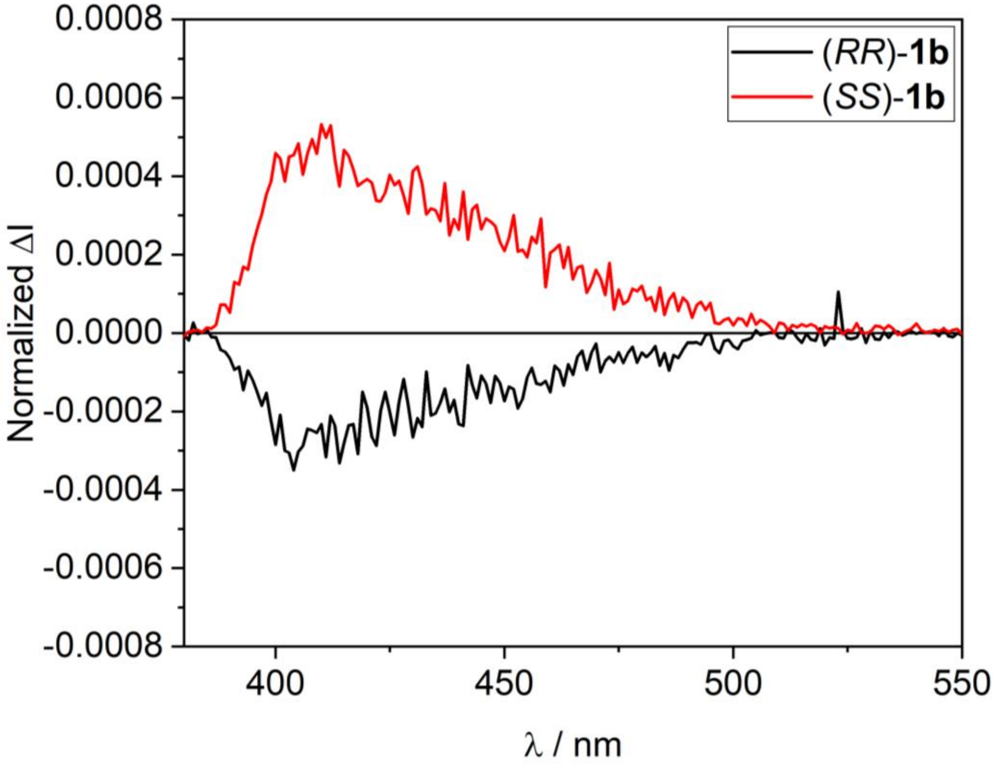

Figure S41. CPL (λ_{exc} = 403 nm) spectra of (*RR*)/(*SS*)-**1b** in CH_2Cl_2

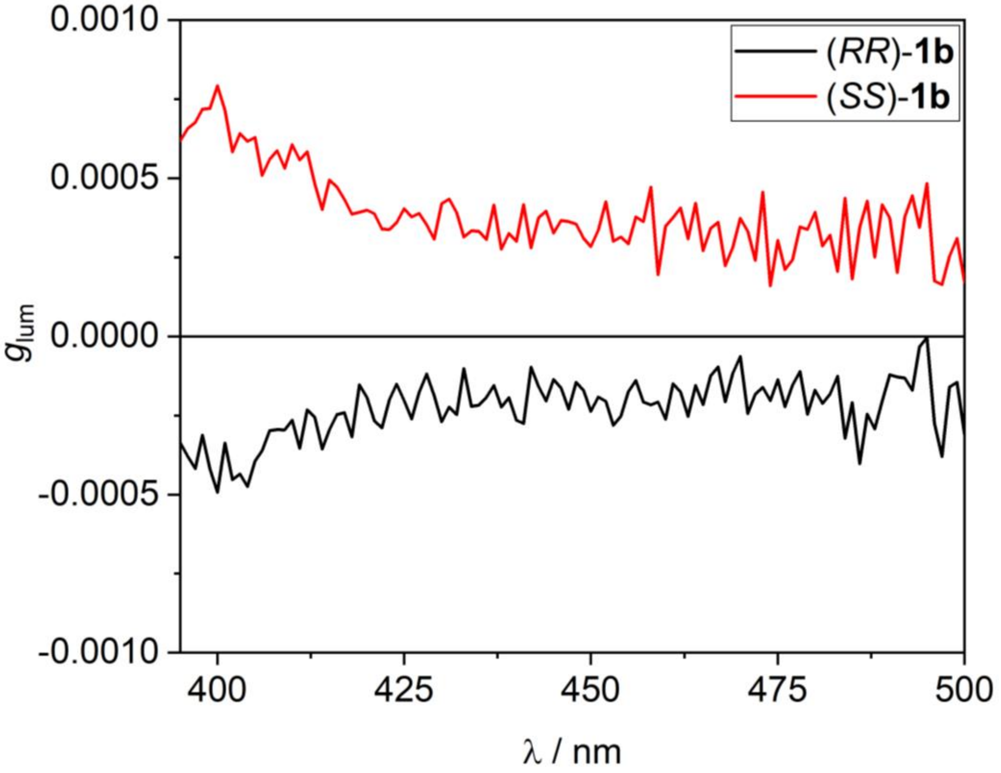

Figure S42. g_{lum} (λ_{exc} = 403 nm) spectra of (*RR*)/(*SS*)-**1b** in CH_2Cl_2 ($|g_{lum}|$ = 4.6 × 10^{-4})

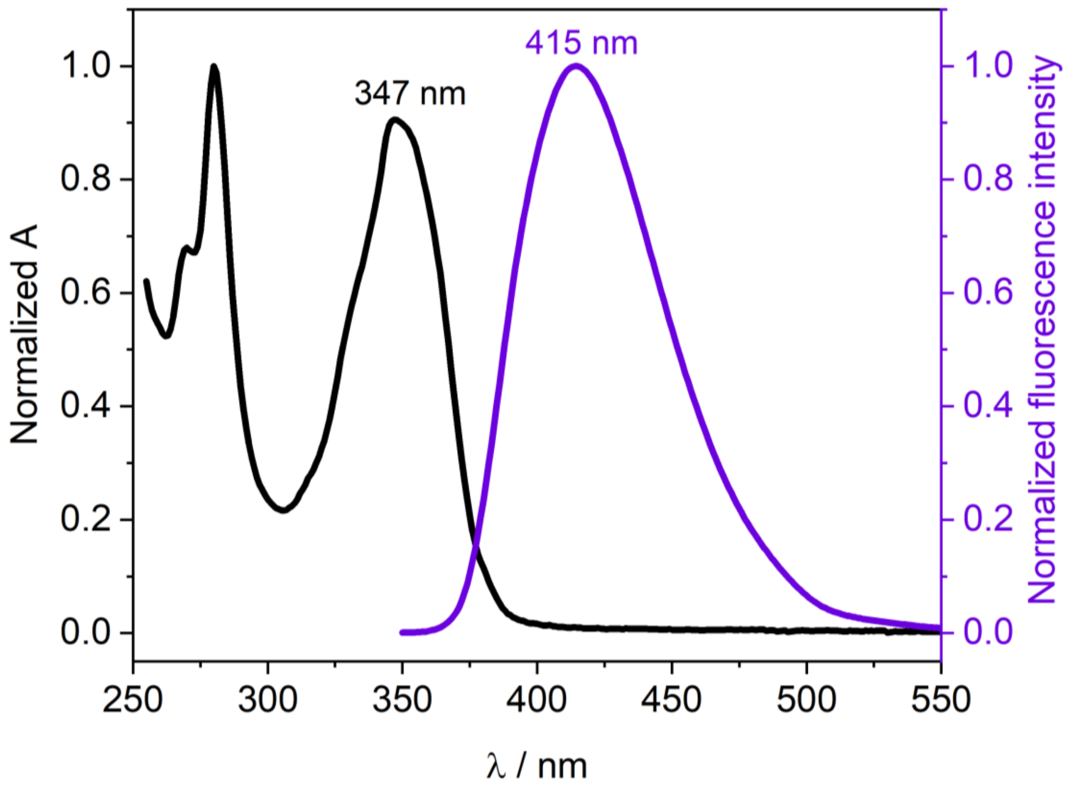

Figure S43. Normalized absorption (black line) and emission (purple line; λ_{exc} = 340 nm) spectra of **2a** in CH_2Cl_2

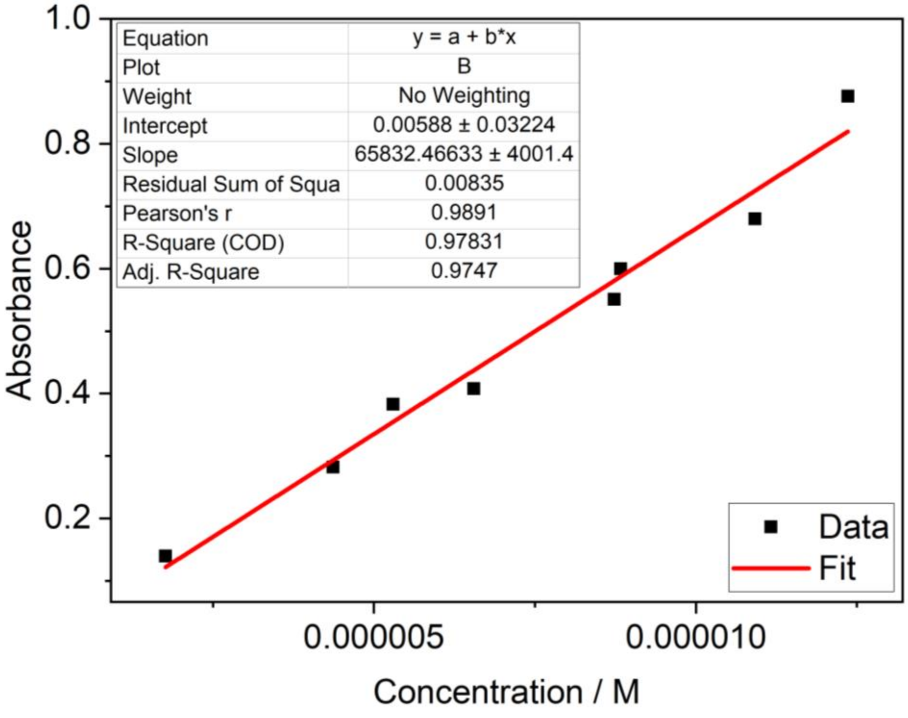

Figure S44. Plot of the absorbance at the absorption λ_{max} of **2a** versus concentration. The molar extinction coefficient (ε) is 6.6 × 10^4 L mol^{-1} cm^{-1} at 347 nm in CH_2Cl_2

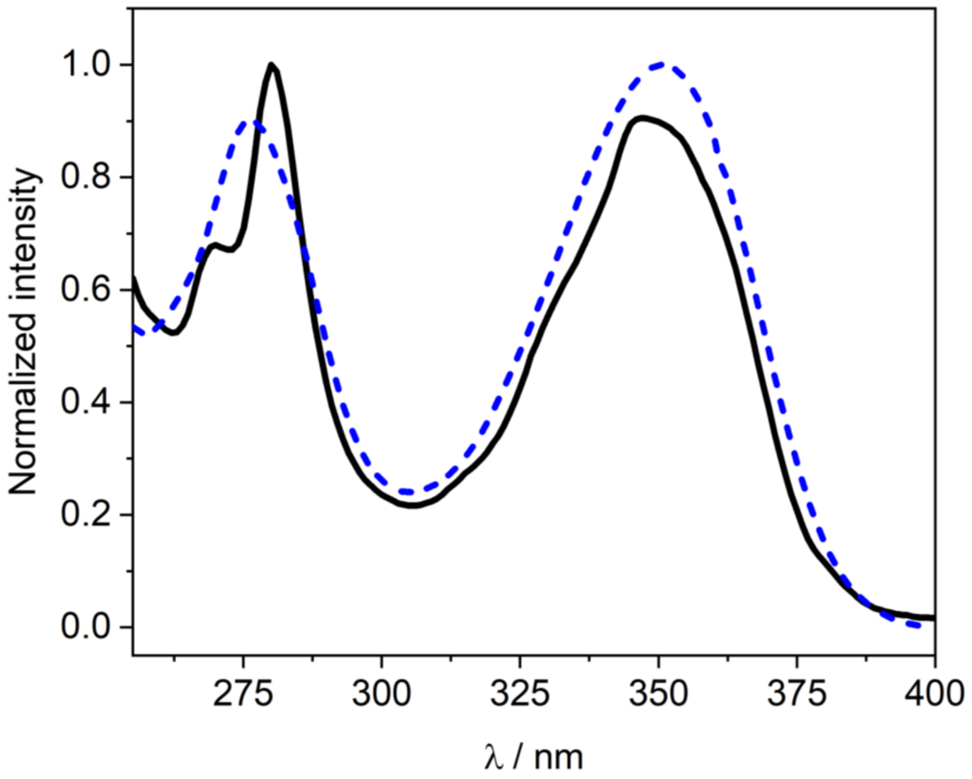

Figure S45. Comparison of the normalized absorption (black line) and excitation (blue dashed line; λ_{em} = 415 nm) spectra of **2a** in CH_2Cl_2

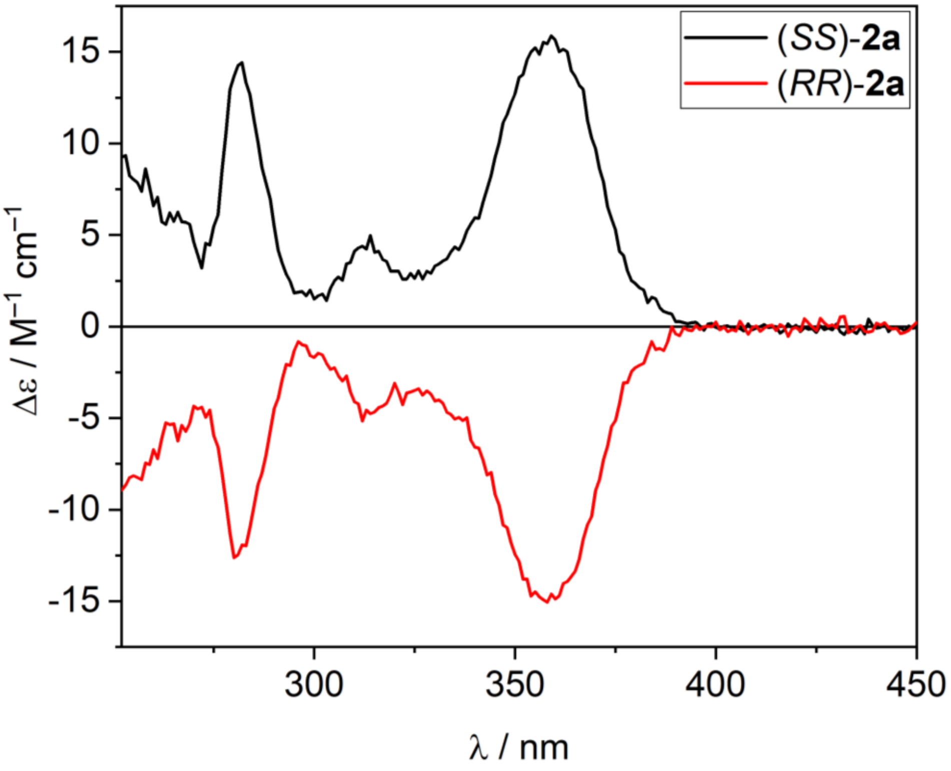

Figure S46. ECD spectra of (*RR*)/(*SS*)-**2a** in CH_2Cl_2

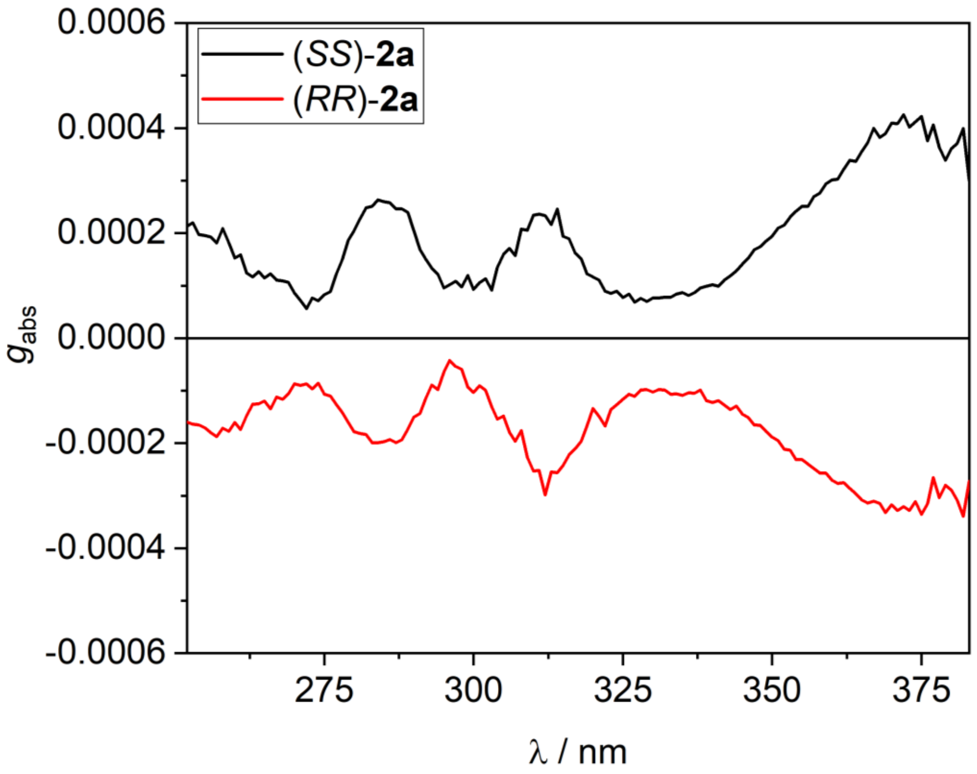

Figure S47. g_{abs} spectra of (*RR*)/(*SS*)-**2a** in CH_2Cl_2 (lowest energy transition $|g_{abs}| = 3 \times 10^{-4}$)

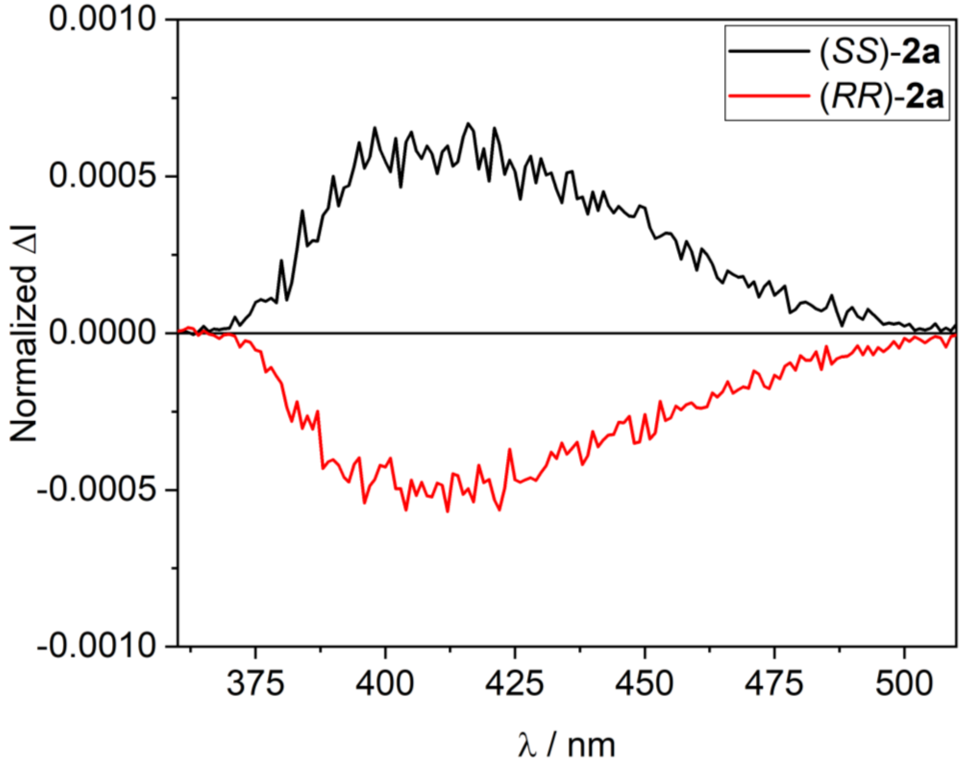

Figure S48. CPL (λ_{exc} = 372 nm) spectra of (*RR*)/(*SS*)-**2a** in CH_2Cl_2

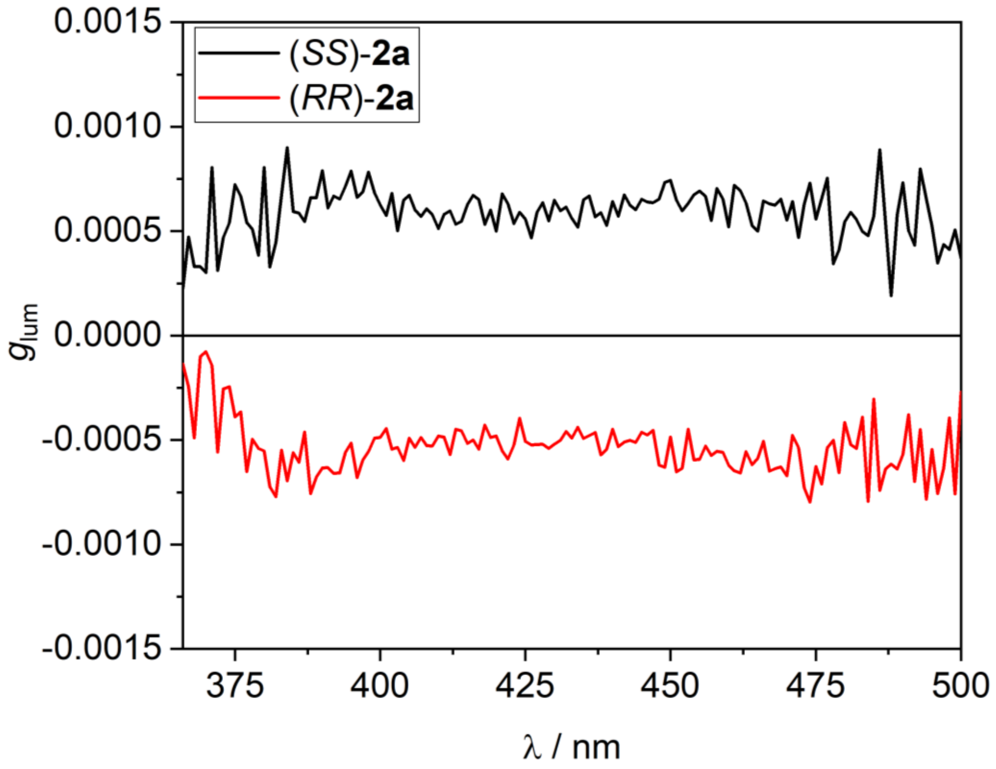

Figure S49. g_{lum} (λ_{exc} = 372 nm) spectra of (*RR*)/(*SS*)-**2a** in CH_2Cl_2 ($|g_{lum}| = 5.8 \times 10^{-4}$)

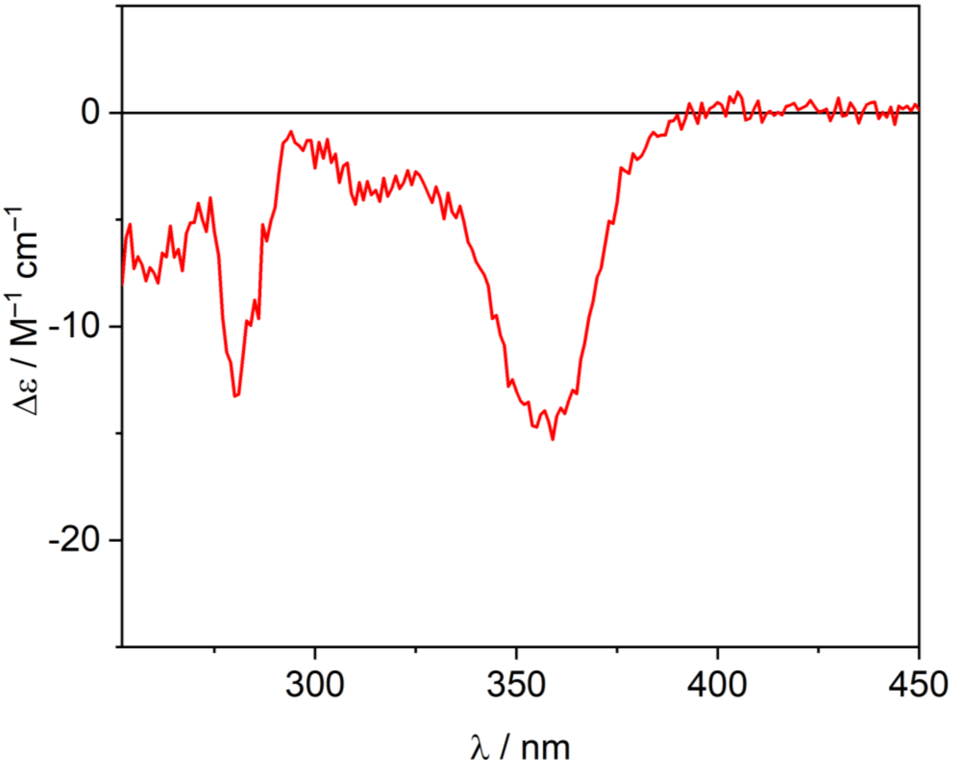

Figure S50. ECD spectrum (CH_2Cl_2) of (*RR*)-**2a** obtained via the diastereoisomeric resolution of (*RR*)-**5** by co-crystallization followed by a Suzuki reaction.

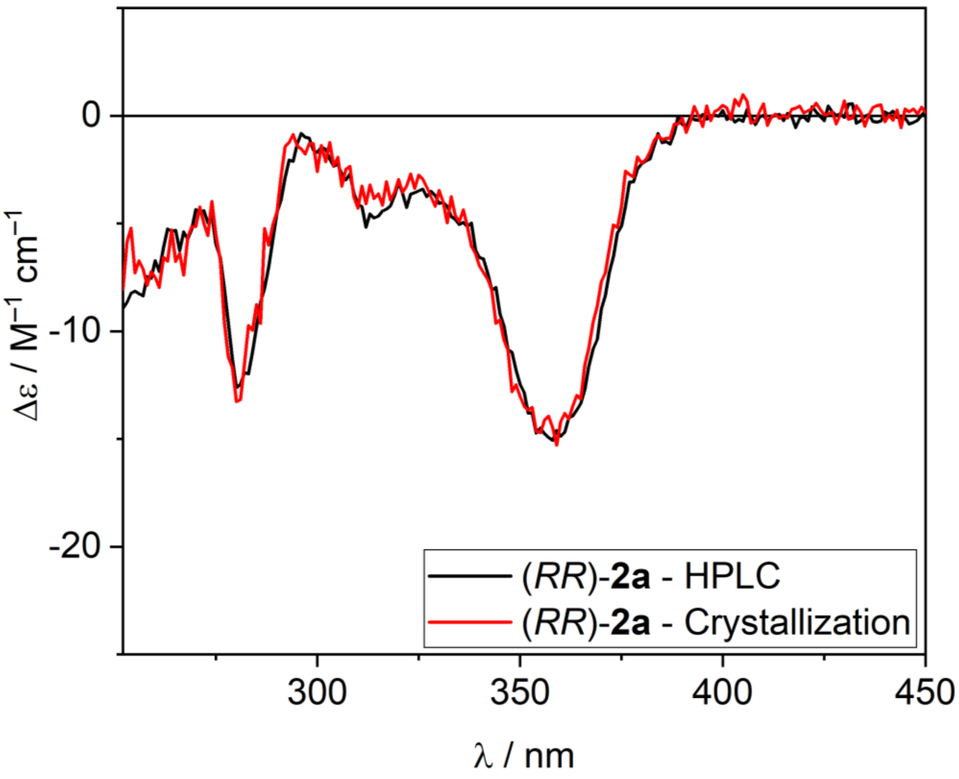

Figure S51. Comparison between ECD spectra (CH_2Cl_2) of (*RR*)-**2a** obtained via the diastereoisomeric resolution of (*RR*)-**5** by co-crystallization followed by a Suzuki reaction (red line) or by the chiral stationary phase HPLC method (black line). These ECD spectra are very similar which demonstrate the enantiopurity of (*RR*)-**2a** obtained via the diastereoisomeric resolution of (*RR*)-**5**.

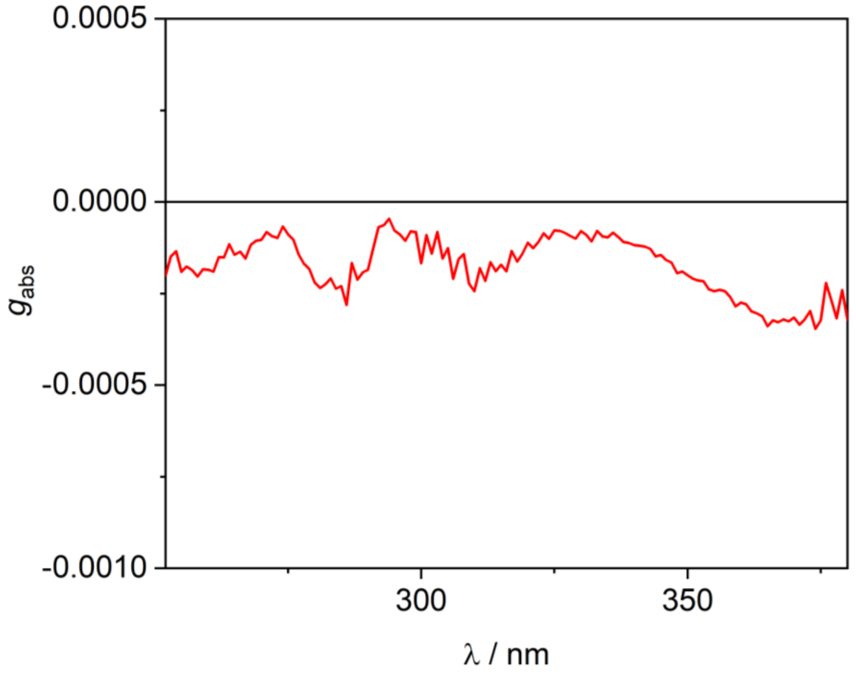

Figure S52. g_{abs} spectrum (CH_2Cl_2) of (*RR*)-**2a** obtained via the diastereoisomeric resolution of (*RR*)-**5** by co-crystallization followed by a Suzuki reaction.

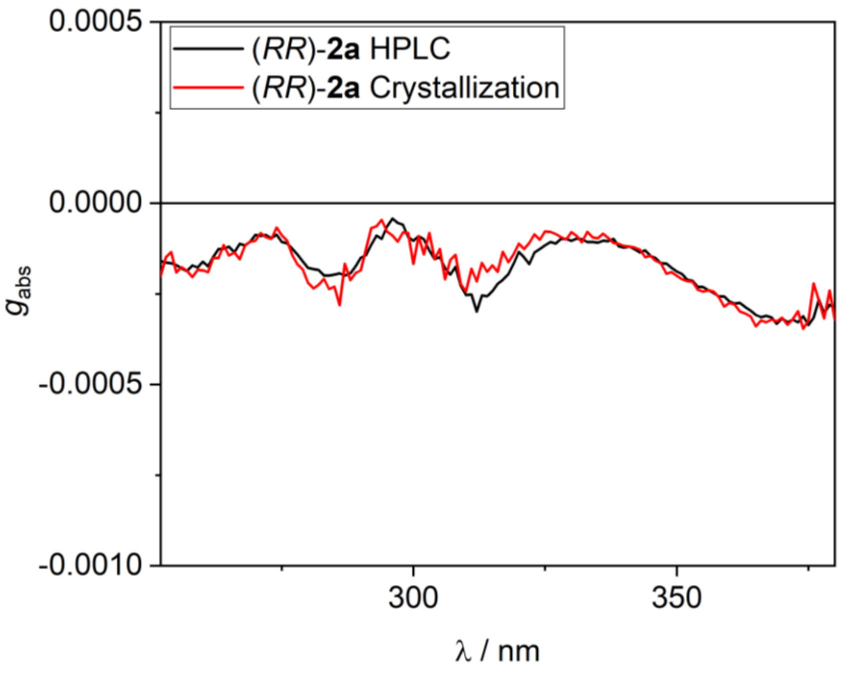

Figure S53. Comparison between g_{abs} spectra (CH_2Cl_2) of (*RR*)-**2a** obtained via the diastereoisomeric resolution of (*RR*)-**5** by co-crystallization followed by a Suzuki reaction (red line) or by the chiral stationary phase HPLC method (black line). These g_{abs} spectra are very similar which demonstrate the enantiopurity of (*RR*)-**2a** obtained via the diastereoisomeric resolution of (*RR*)-**5**.

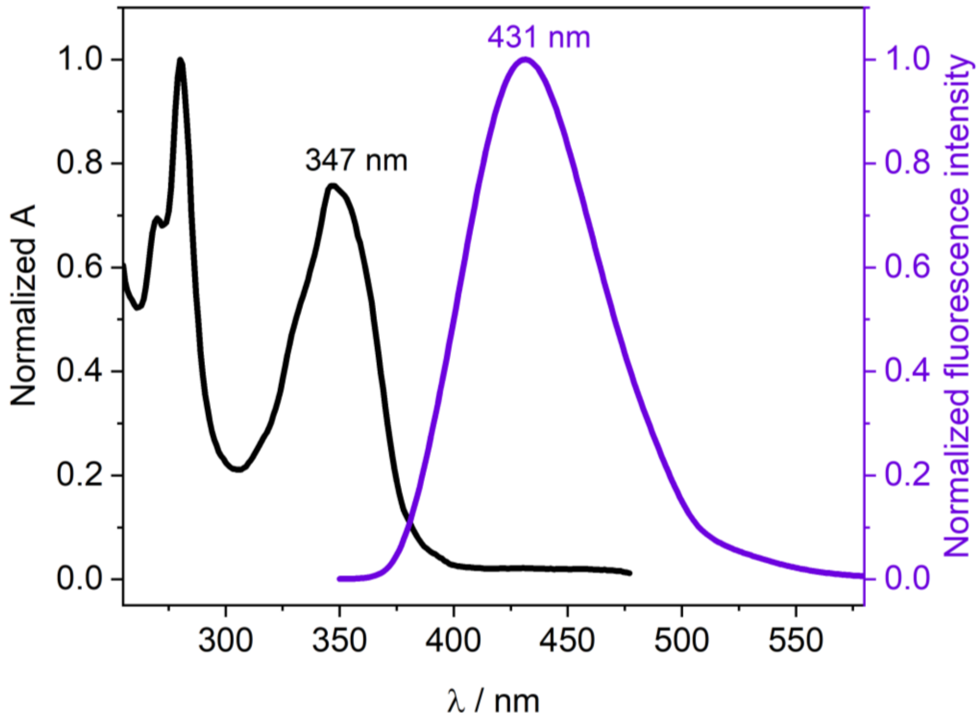

Figure S54. Normalized absorption (black line) and emission (purple line; λ_{exc} = 340 nm) spectra of **2b** in CH_2Cl_2

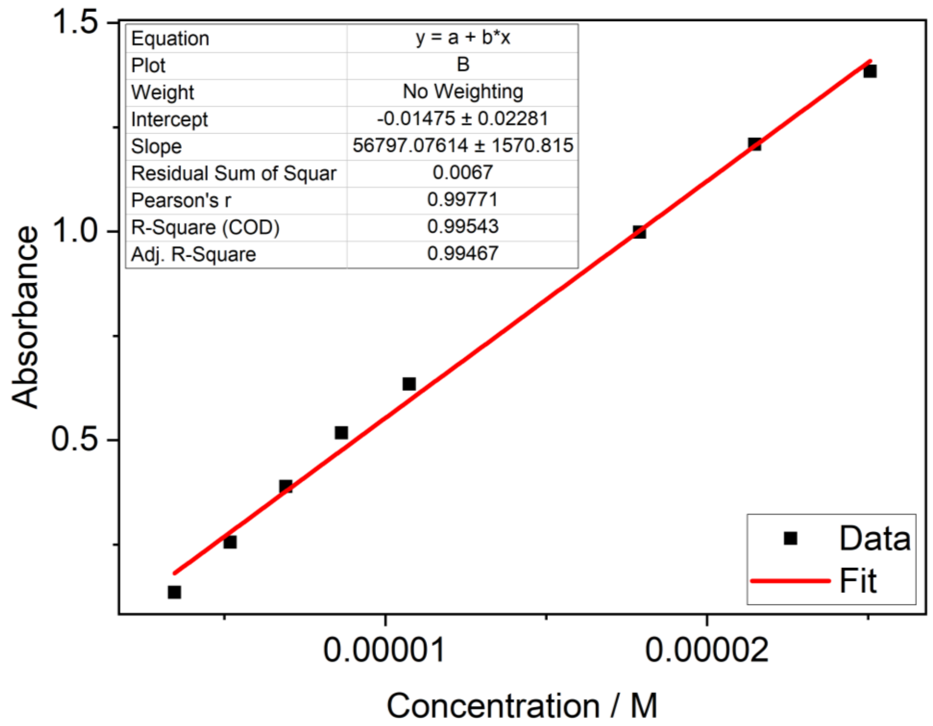

Figure S55. Plot of the absorbance at the absorption λ_{max} of **2b** versus concentration. The molar extinction coefficient (ε) is 5.7×10^4 L mol^{-1} cm^{-1} at 347 nm in CH_2Cl_2

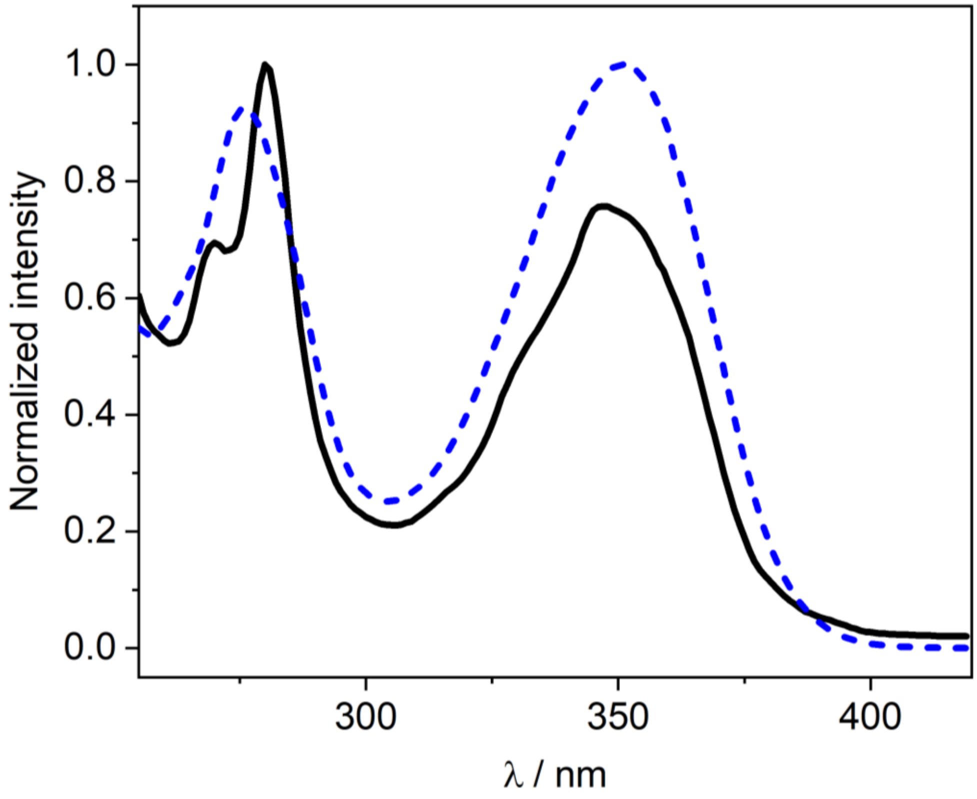

Figure S56. Comparison of the normalized absorption (black line) and excitation (blue dashed line; λ_{em} = 430 nm) spectra of **2b** in CH_2Cl_2

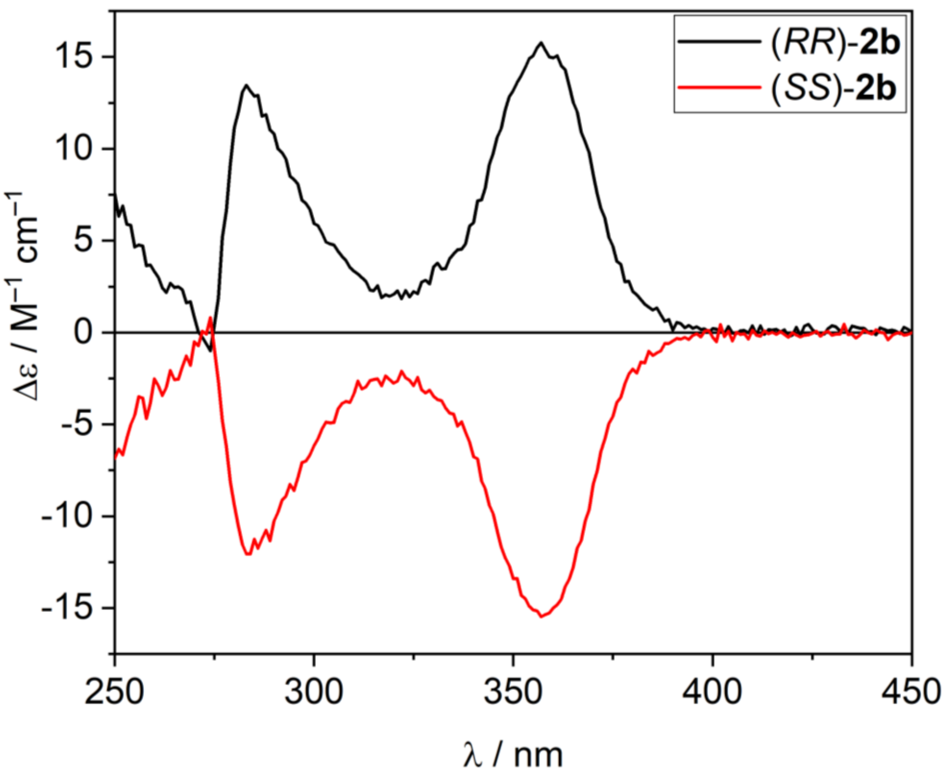

Figure S57. ECD spectra of (*RR*)/(*SS*)-**2b** in CH_2Cl_2

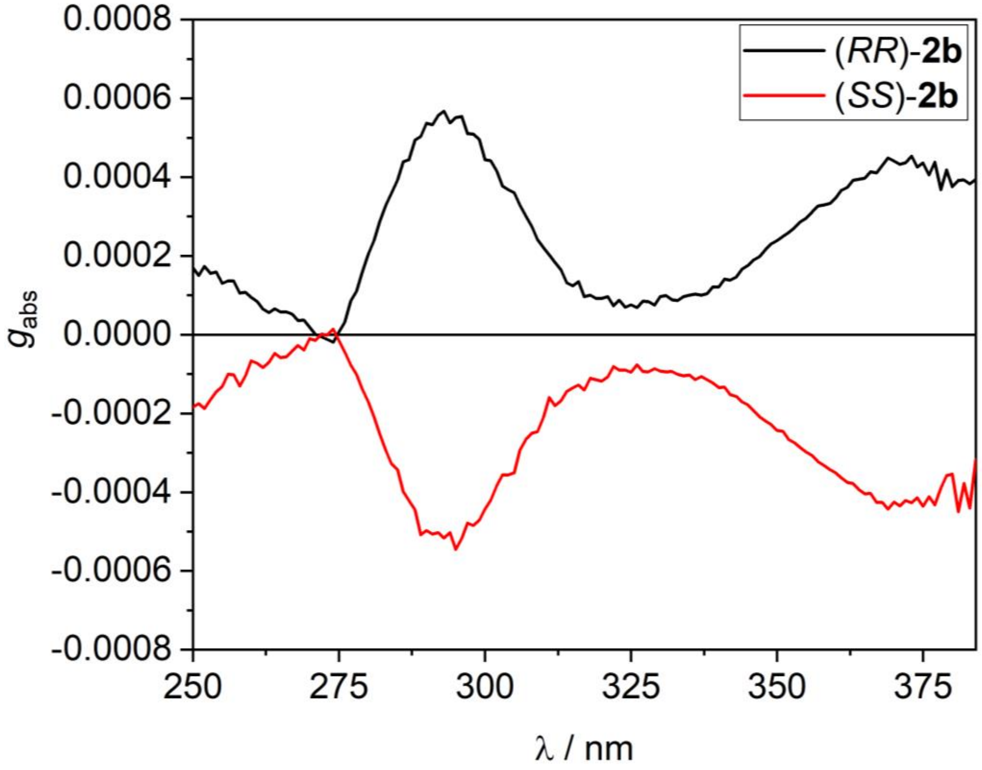

Figure S58. g_{abs} spectra of (*RR*)/(*SS*)-**2b** in CH_2Cl_2 (lowest energy transition $|g_{abs}| = 3 \times 10^{-4}$)

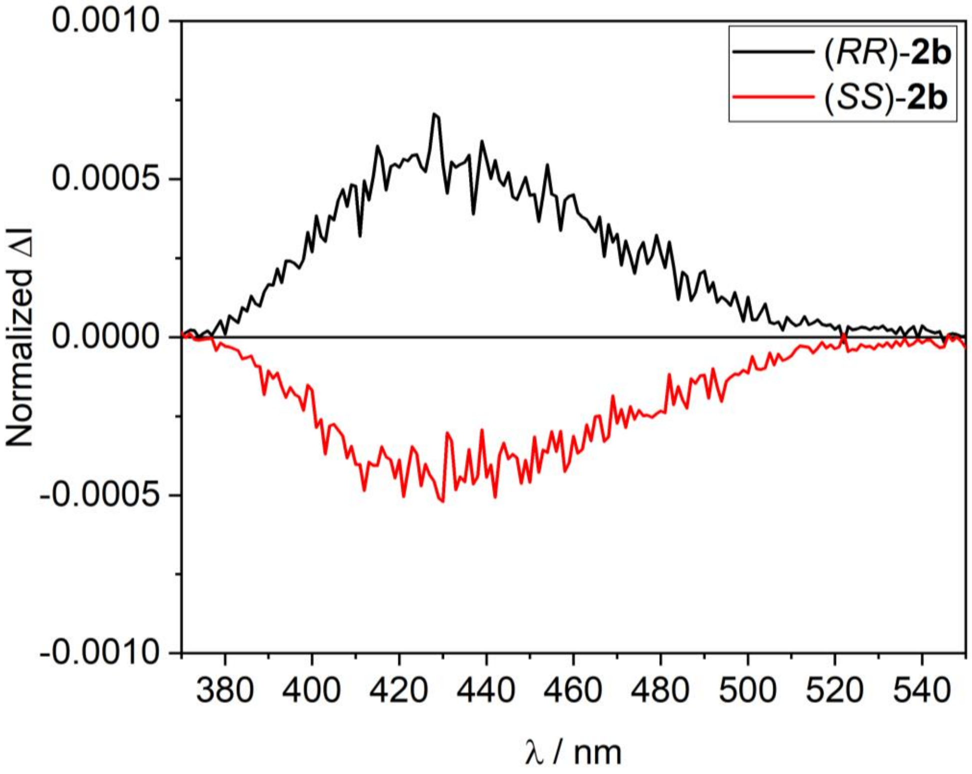

Figure S59. CPL (λ_{exc} = 372 nm) spectra of (*RR*)/(*SS*)-**2b** in CH_2Cl_2

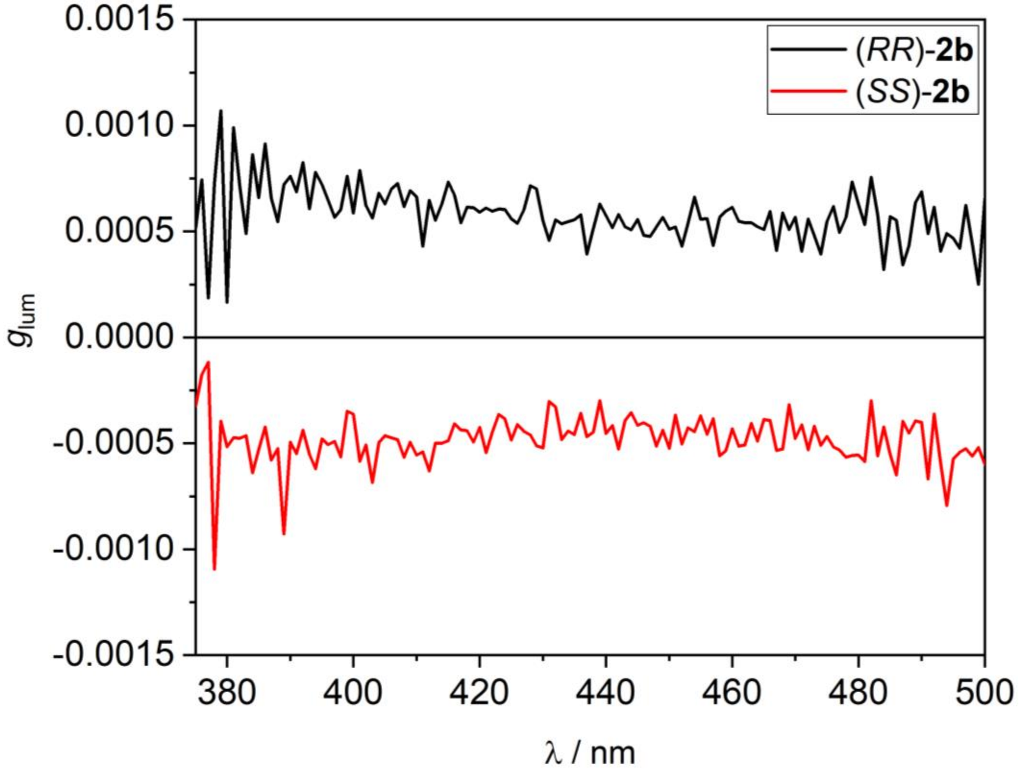

Figure S60. g_{lum} (λ_{exc} = 372 nm) spectra of (*RR*)/(*SS*)-**2b** in CH_2Cl_2 ($|g_{lum}| = 5.3 \times 10^{-4}$)

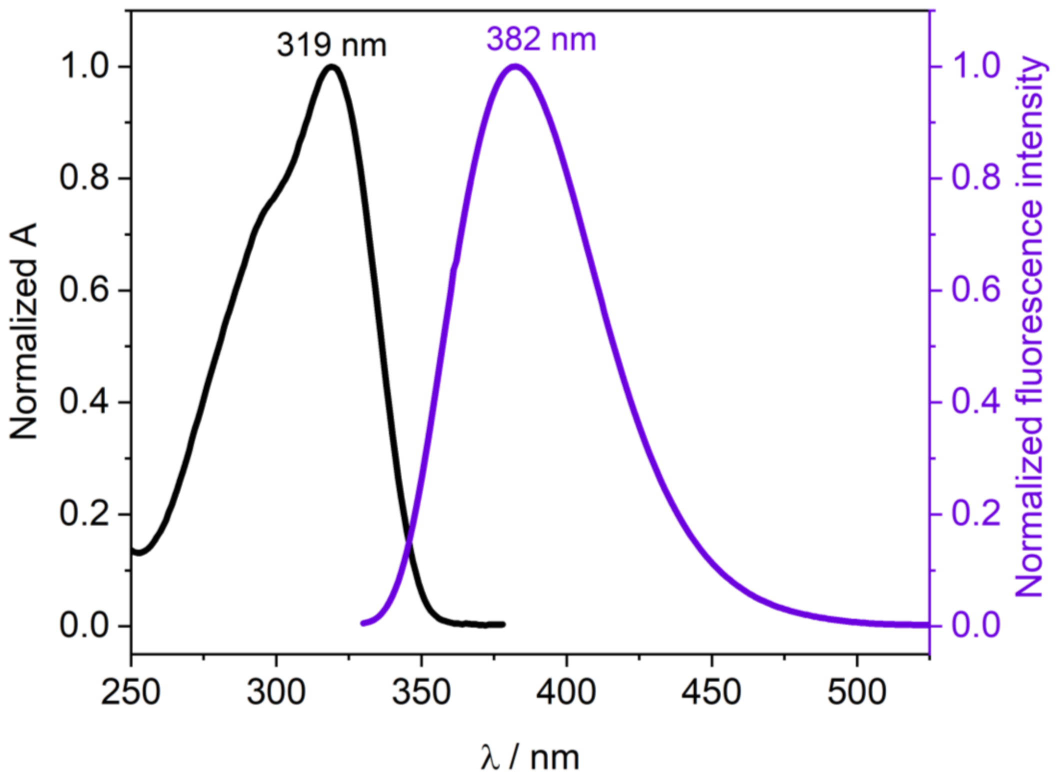

Figure S61. Normalized absorption (black line) and emission (purple line; λ_{exc} = 320 nm) spectra of **3a** in CH_2Cl_2

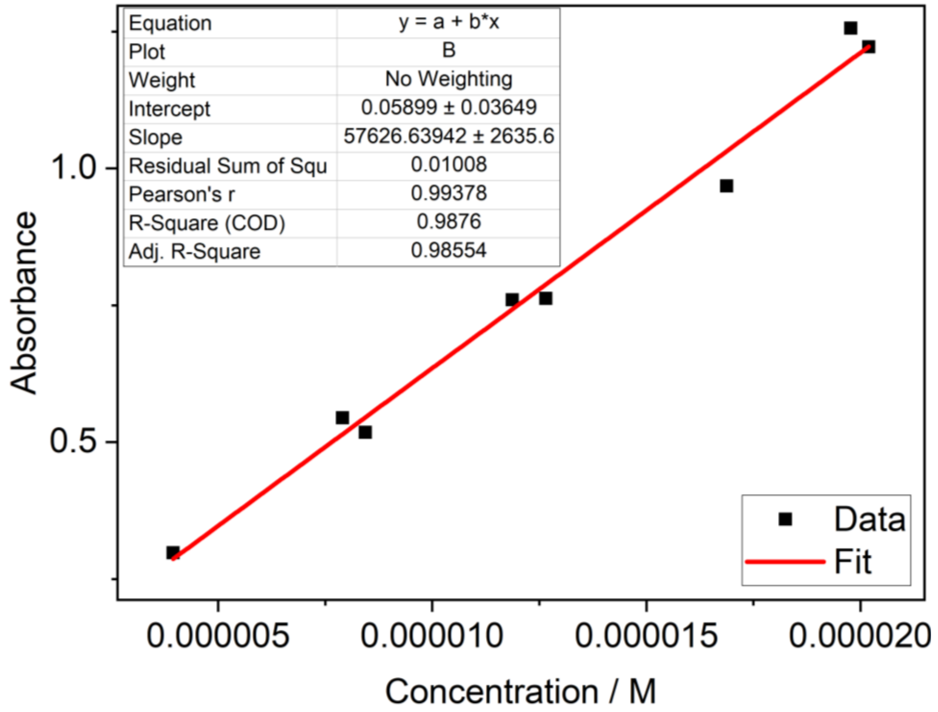

Figure S62. Plot of the absorbance at the absorption λ_{max} of **3a** versus concentration. The molar extinction coefficient (ε) is 5.8 × 10^4 L mol^{-1} cm^{-1} at 319 nm in CH_2Cl_2

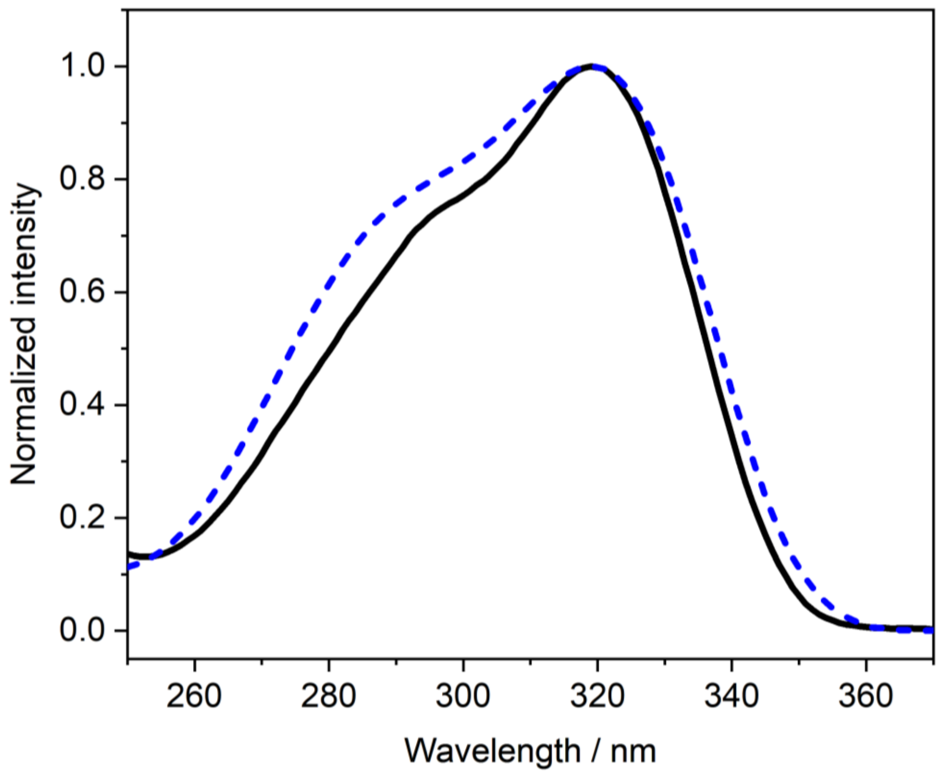

Figure S63. Comparison of the normalized absorption (black line) and excitation (blue dashed line; λ_{em} = 380 nm) spectra of **3a** in CH_2Cl_2

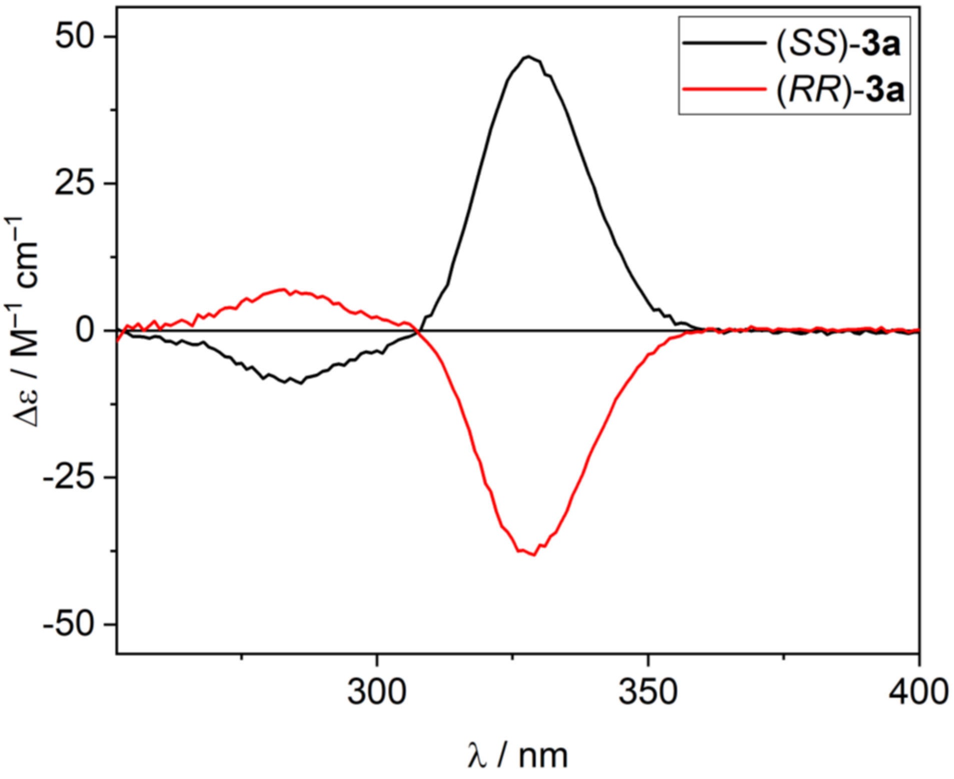

Figure S64. ECD spectra of (*RR*)/(*SS*)-**3a** in CH_2Cl_2

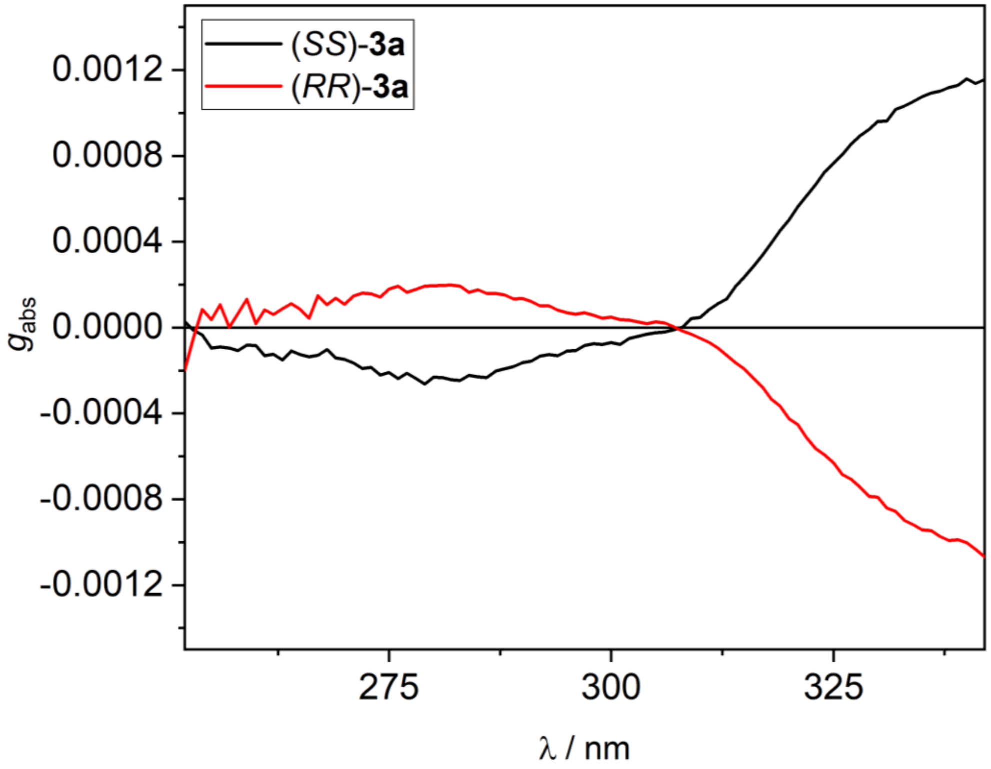

Figure S65. g_{abs} spectra of (*RR*)/(*SS*)-**3a** in CH_2Cl_2 (lowest energy transition $|g_{abs}| = 9 \times 10^{-4}$)

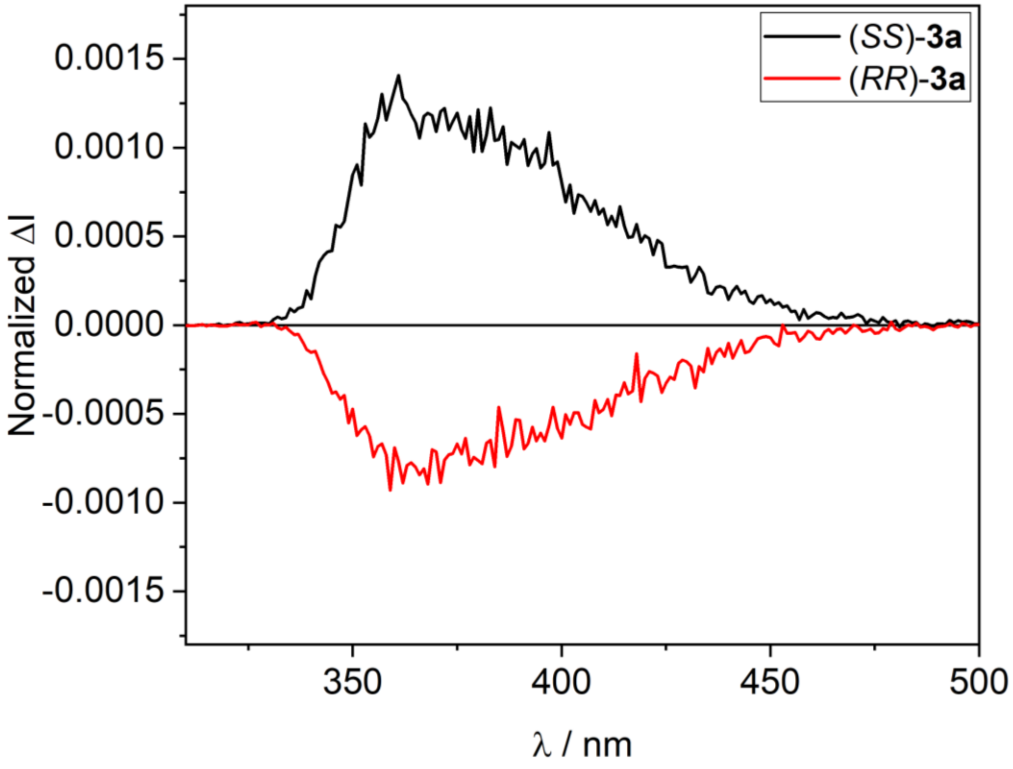

Figure S66. CPL (λ_{exc} = 340 nm) spectra of (*RR*)/(*SS*)-**3a** in CH_2Cl_2

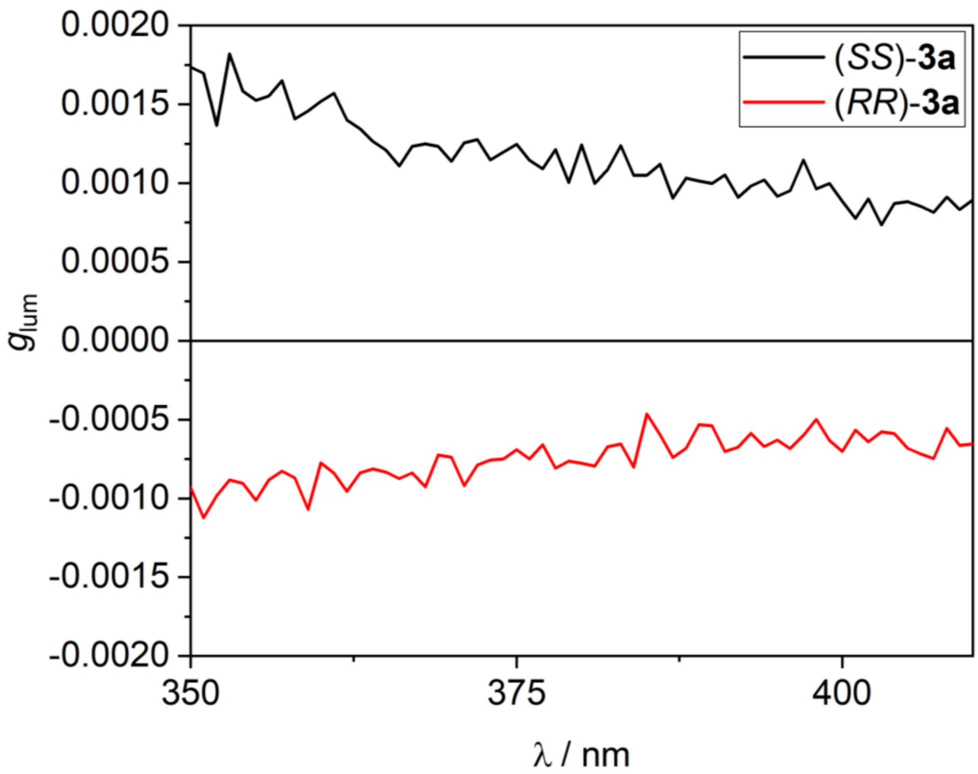

Figure S67. g_{lum} (λ_{exc} = 340 nm) spectra of (*RR*)/(*SS*)-**3a** in CH_2Cl_2 (|g_{lum}| = 1.2 × 10^{-3})

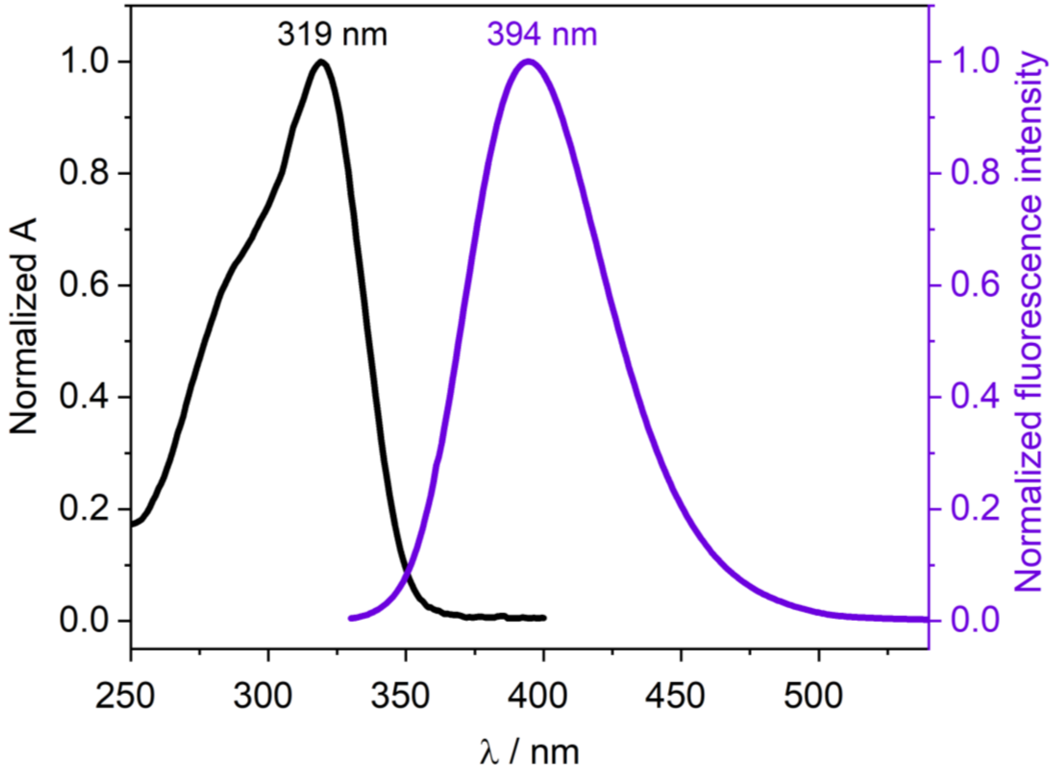

Figure S68. Normalized absorption (black line) and emission (purple line; λ_{exc} = 320 nm) spectra of **3b** in CH_2Cl_2

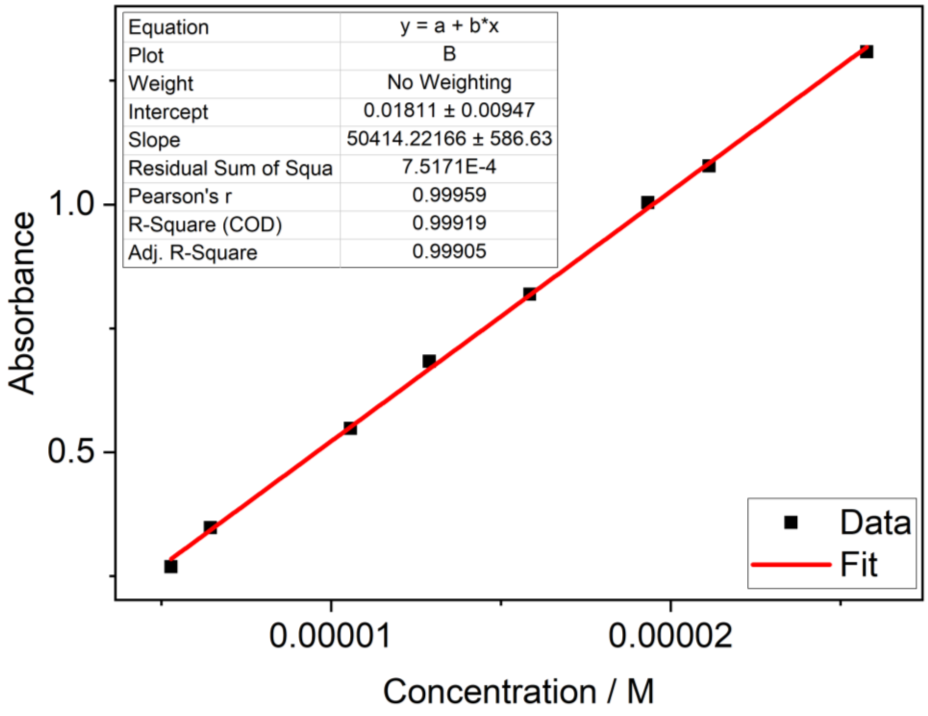

Figure S69. Plot of the absorbance at the absorption λ_{max} of **3b** versus concentration. The molar extinction coefficient (ε) is 5.0×10^4 L mol^{-1} cm^{-1} at 319 nm in CH_2Cl_2

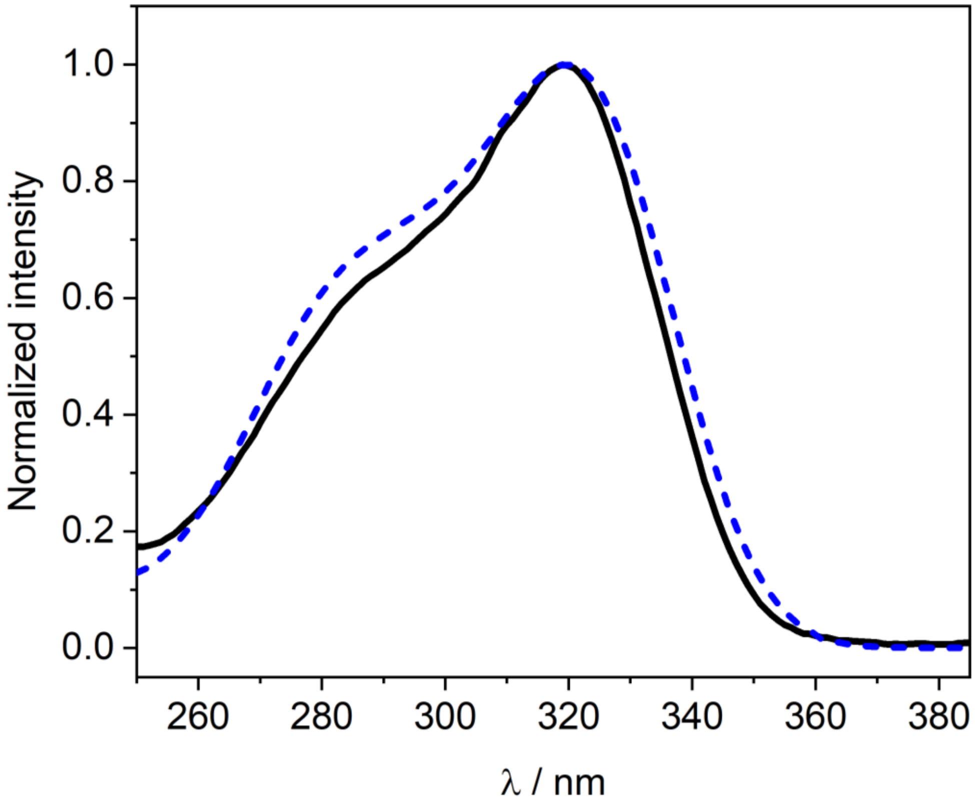

Figure S70. Comparison of the normalized absorption (black line) and excitation (blue dashed line; λ_{em} = 395 nm) spectra of **3b** in CH_2Cl_2

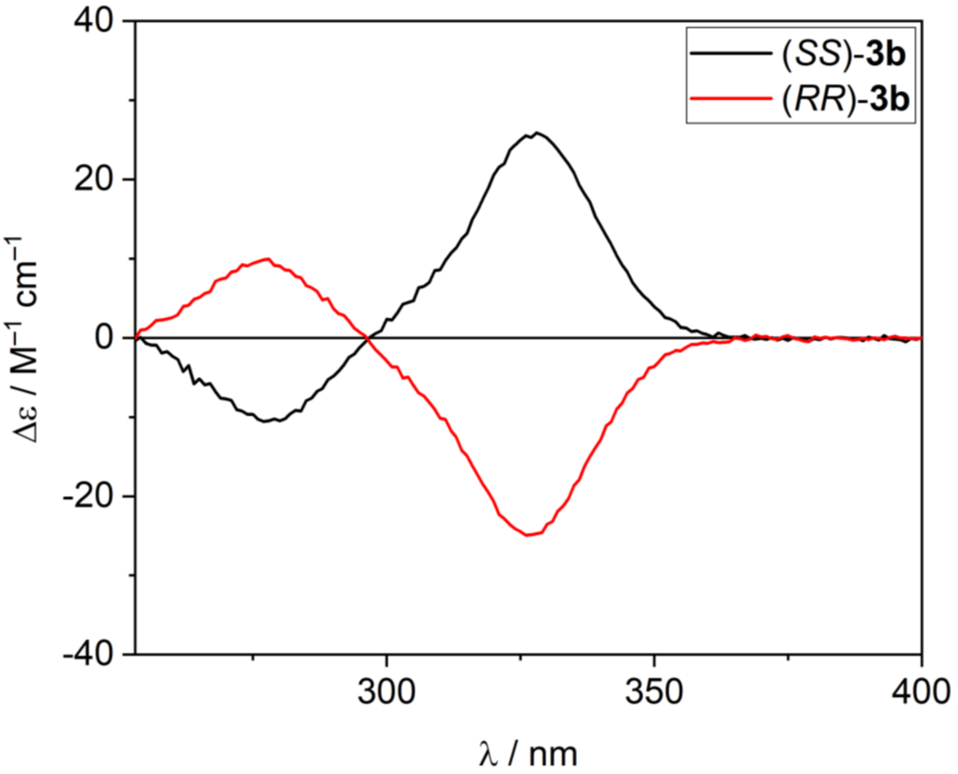

Figure S71. ECD spectra of (*RR*)/(*SS*)-**3b** in CH_2Cl_2

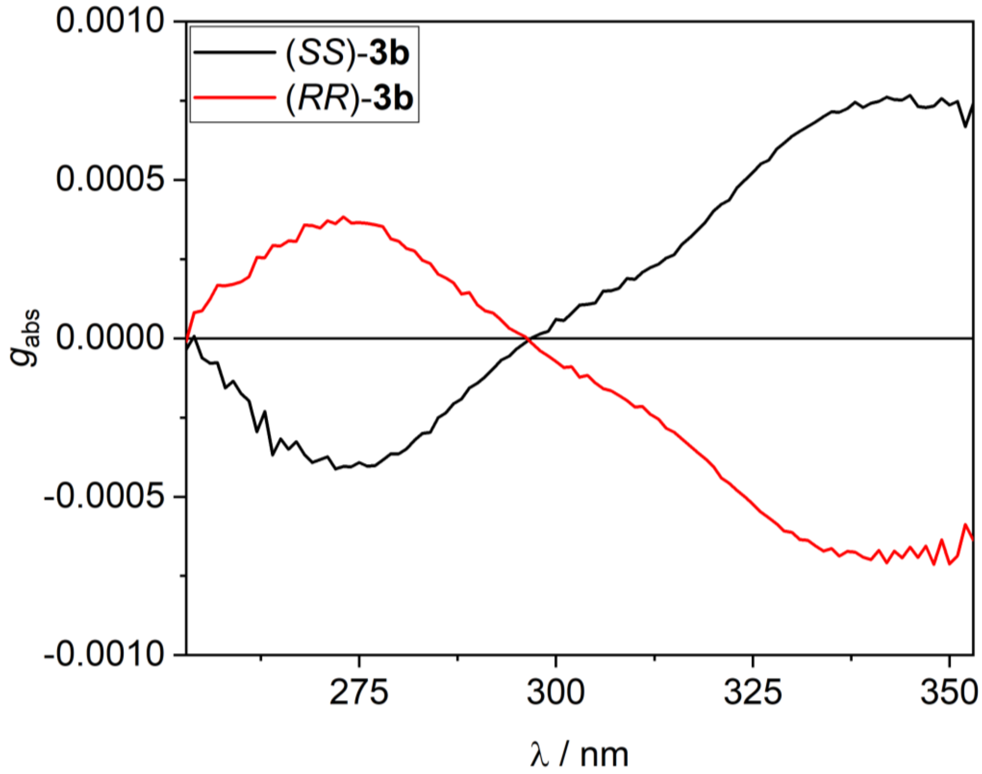

Figure S72. g_{abs} spectra of (*RR*)/(*SS*)-**3b** in CH_2Cl_2 (lowest energy transition $|g_{abs}| = 6 \times 10^{-4}$)

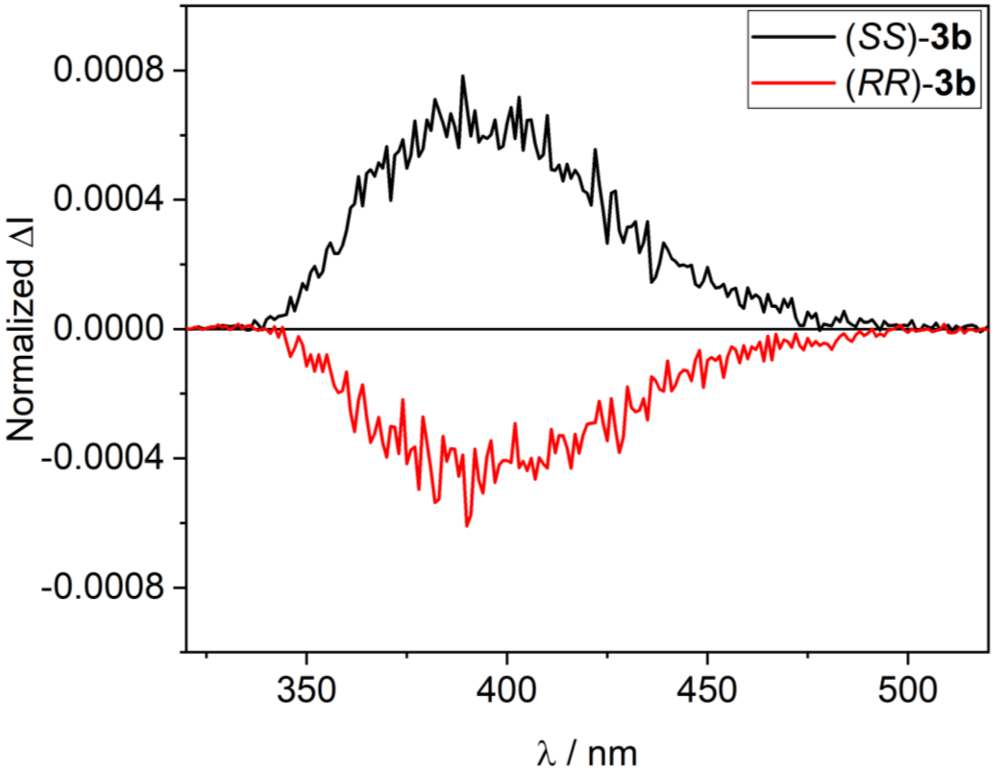

Figure S73. CPL (λ_{exc} = 342 nm) spectra of (*RR*)/(*SS*)-**3b** in CH_2Cl_2

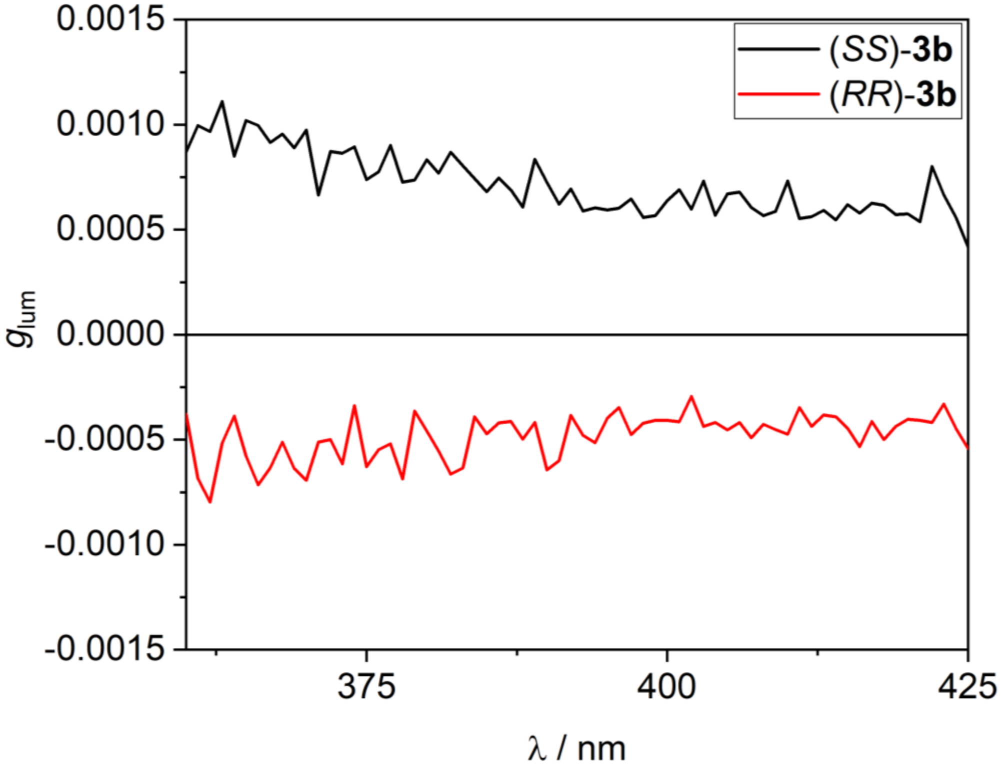

Figure S74. g_{lum} (λ_{exc} = 342 nm) spectra of (*RR*)/(*SS*)-**3b** in CH_2Cl_2 ($|g_{lum}| = 6.5 \times 10^{-4}$)

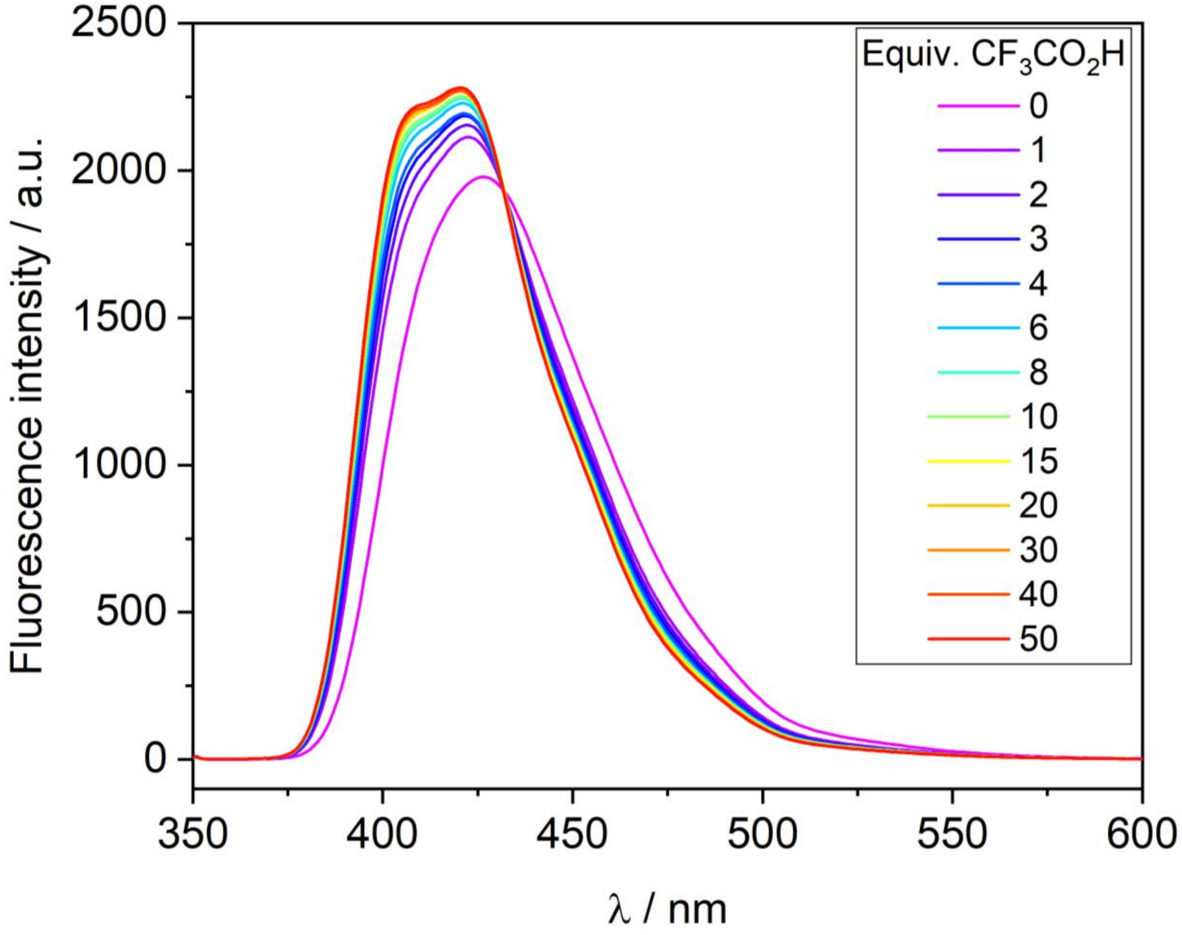

Figure S75. Fluorescence emission spectra (λ_{exc} = 340 nm) for the titration of **1b** with CF_3CO_2H (0 equiv. – 50 equiv.). Φ_F for compound **1b** with 50 equiv. of CF_3CO_2H is 0.670± 0.090.

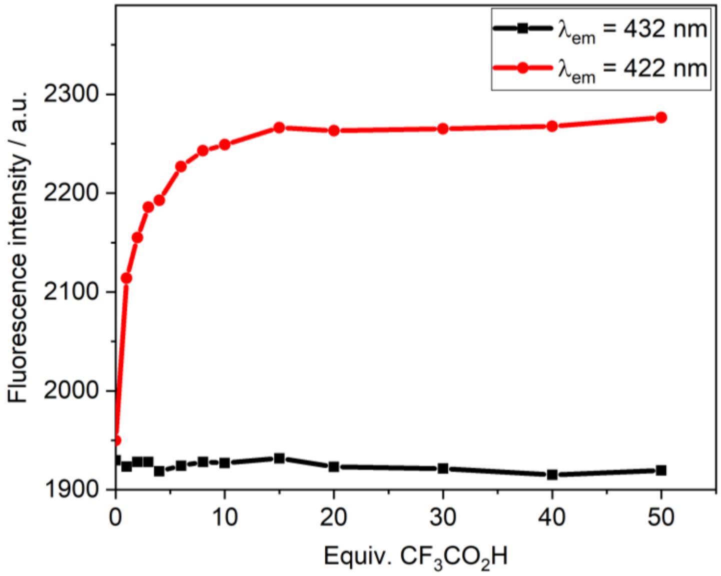

Figure S76. Evolution of the fluorescence intensity (λ_{exc} = 340 nm) at λ_{em} = 422 and 432nm of compound **1b** after the addition of increasing quantities of a CF_3CO_2H solution (0 equiv. – 50 equiv.) in CH_2Cl_2

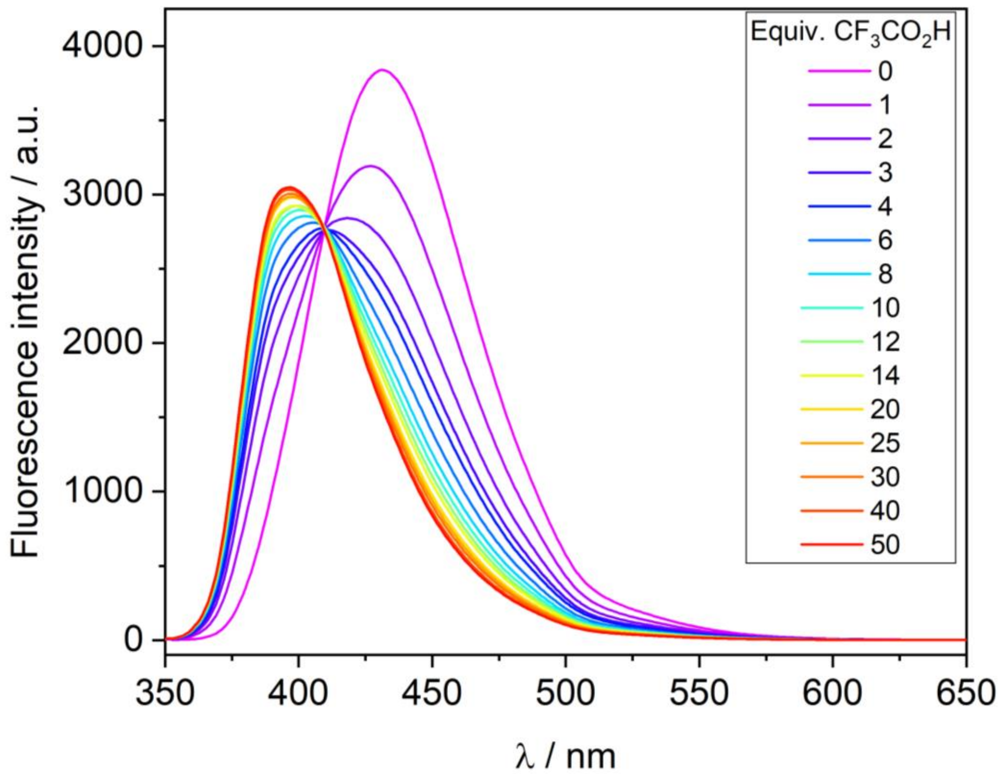

Figure S77. Fluorescence emission spectra (λ_{exc} = 340 nm) for the titration of **2b** with CF_3CO_2H (0 equiv. – 50 equiv.). Φ_F for compound **2b** with 50 equiv. of CF_3CO_2H is 0.447 ± 0.008.

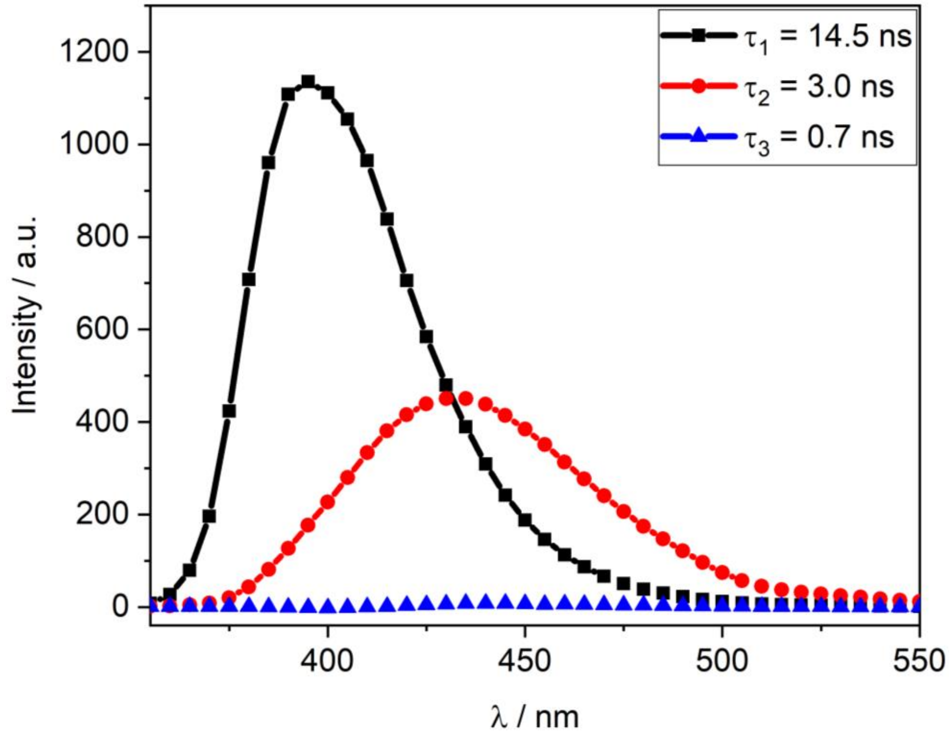

Figure S78. SAEMS spectra of compound **2b** in CH_2Cl_2 in the presence of 50 equiv. of CF_3CO_2H (λ_{exc} = 320nm).

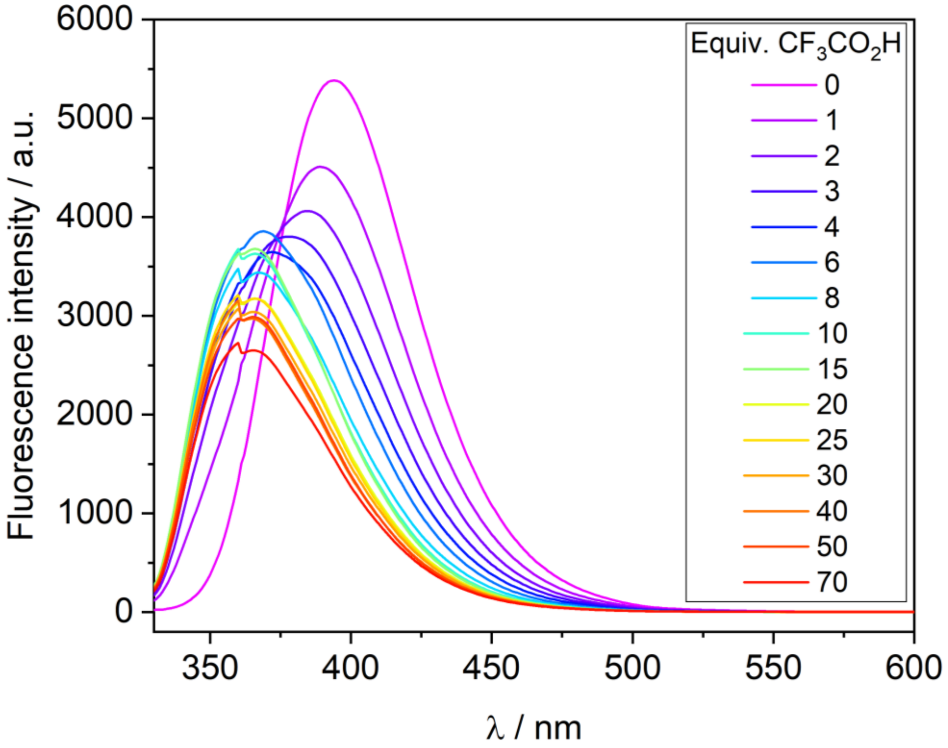

Figure S79. Fluorescence emission spectra (λ_{exc} = 320 nm) for the titration of **3b** with CF_3CO_2H (0 equiv. – 70 equiv.). Φ_F for compound **3b** with 50 equiv. of CF_3CO_2H is 0.466 ± 0.017.

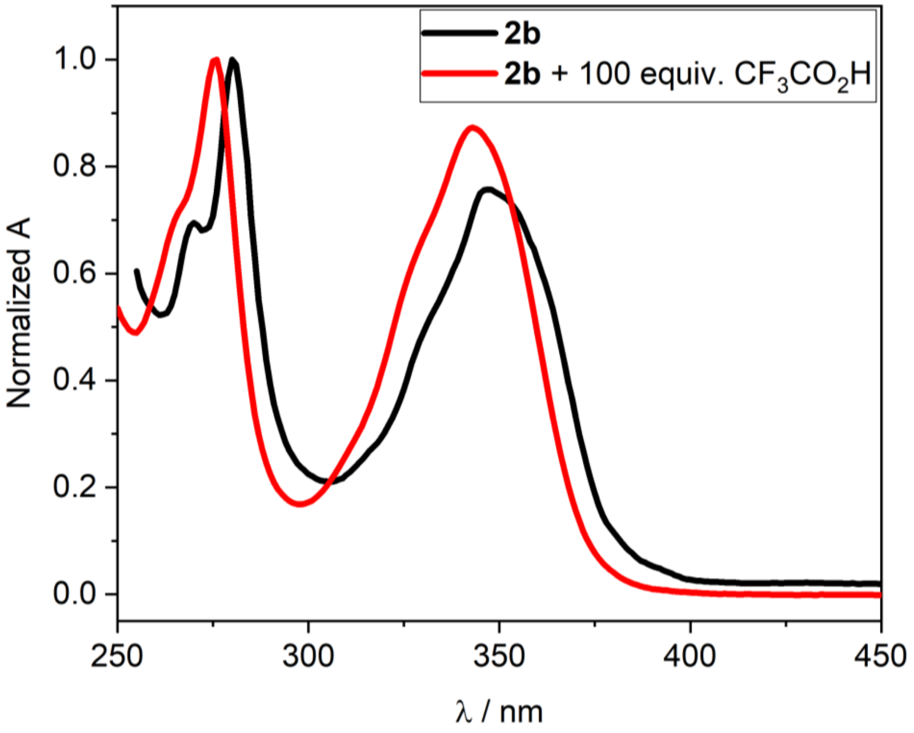

Figure S80. Normalized absorption spectra of **2b** in CH_2Cl_2 in the presence (red line) and the absence (black line) of 100 equiv. of CF_3CO_2H. The molar extinction coefficient (ε) of **2b** in the presence of 100 equiv. of CF_3CO_2H is 5.6 × 10^4 L mol^{-1} cm^{-1} at 343 nm in CH_2Cl_2.

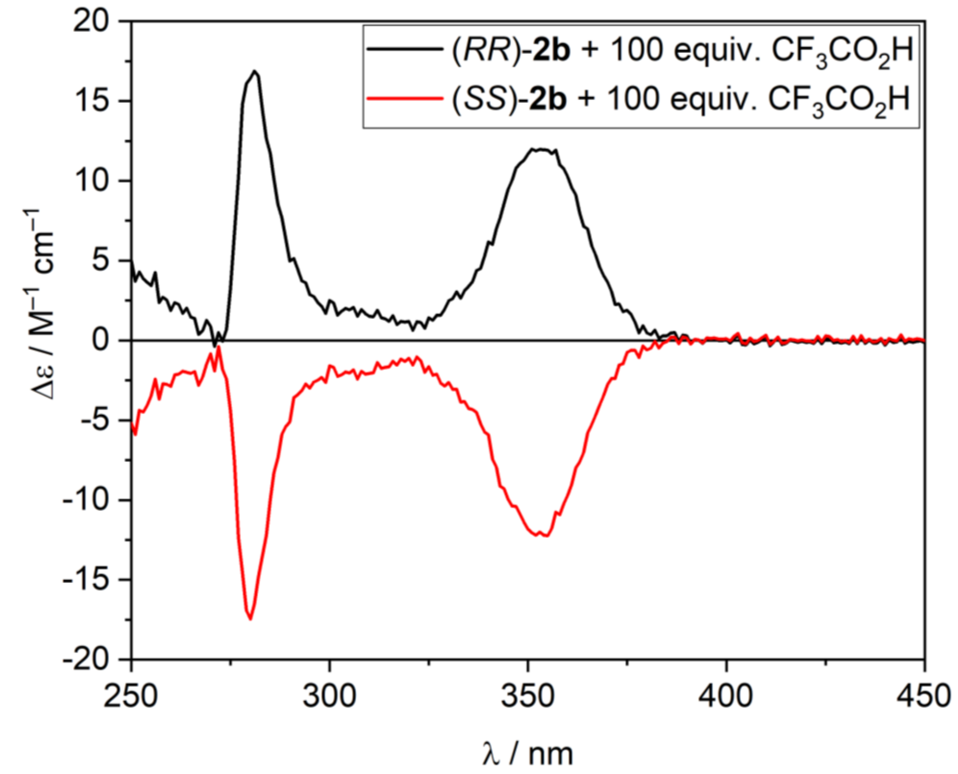

Figure S81. ECD spectra of (*RR*)/(*SS*)-**2b** in CH_2Cl_2 in the presence of 100 equiv. of CF_3CO_2H

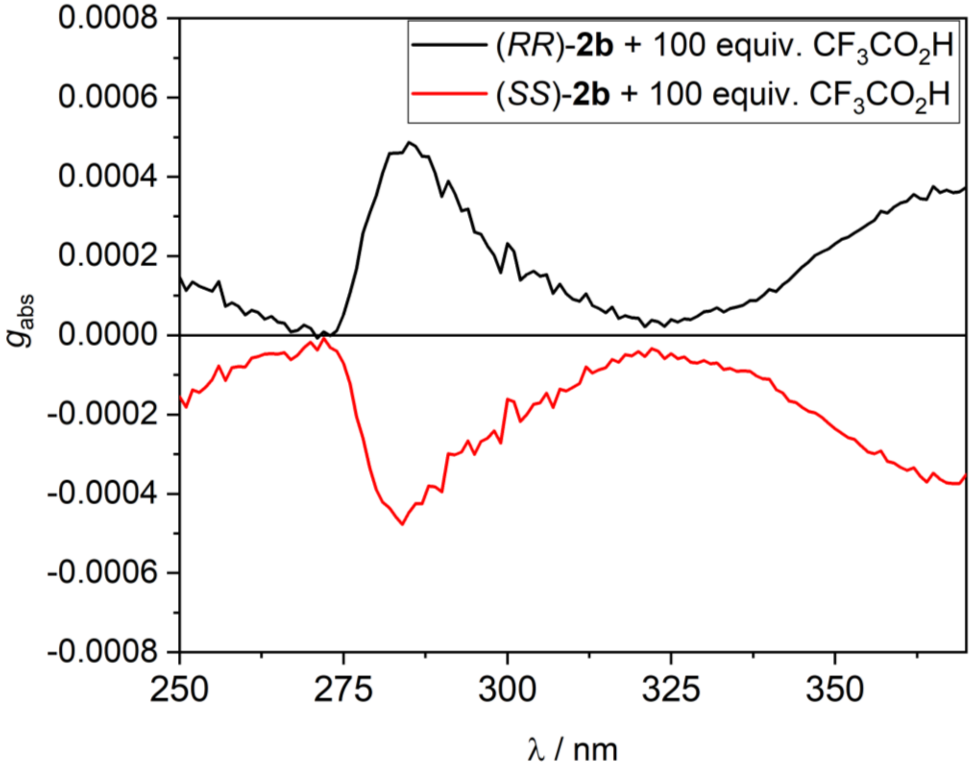

Figure S82. g_{abs} spectra of (*RR*)/(*SS*)-**2b** in CH_2Cl_2 in the presence of 100 equiv. of CF_3CO_2H (lowest energy transition $|g_{abs}| = 4 \times 10^{-4}$)

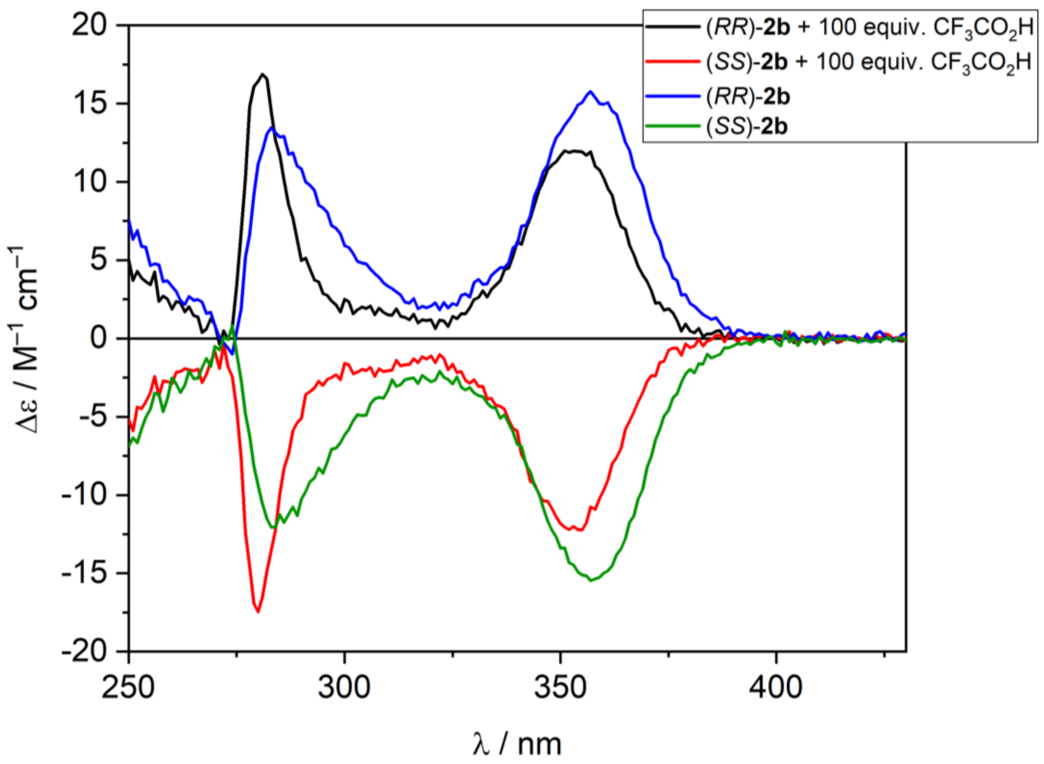

Figure S83. Comparison between the ECD spectra of (*RR*)/(*SS*)-**2b** in CH_2Cl_2 in the presence (black and red lines) and the absence (blue and green lines) of 100 equiv. of CF_3CO_2H

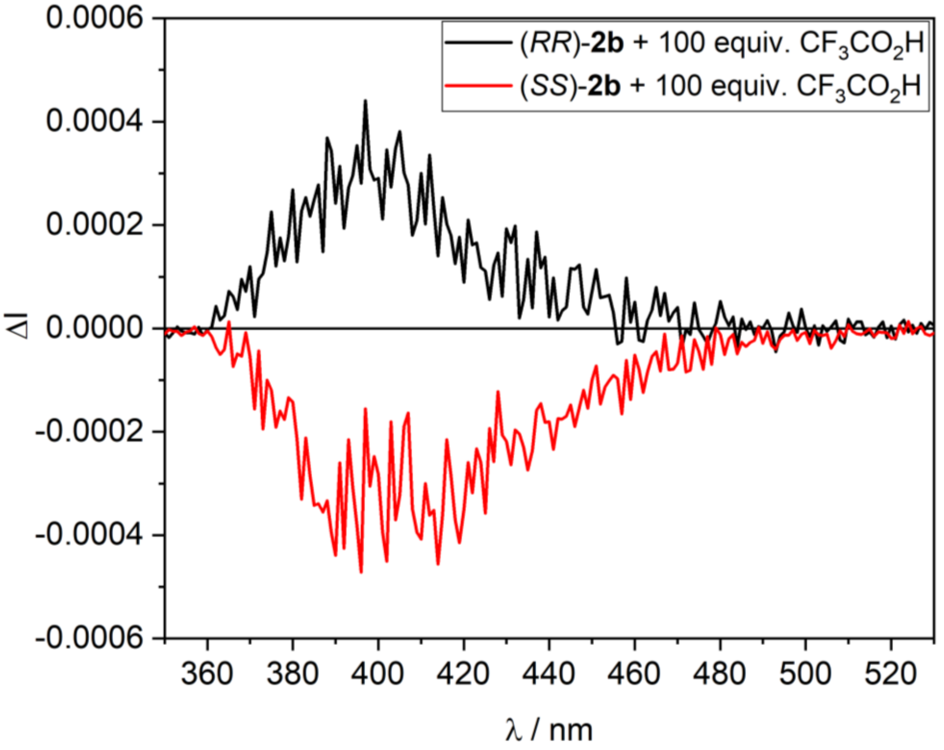

Figure S84. CPL (λ_{exc} = 372 nm) spectra of (*RR*)/(*SS*)-**2b** in CH_2Cl_2 in the presence of 100 equiv. of CF_3CO_2H

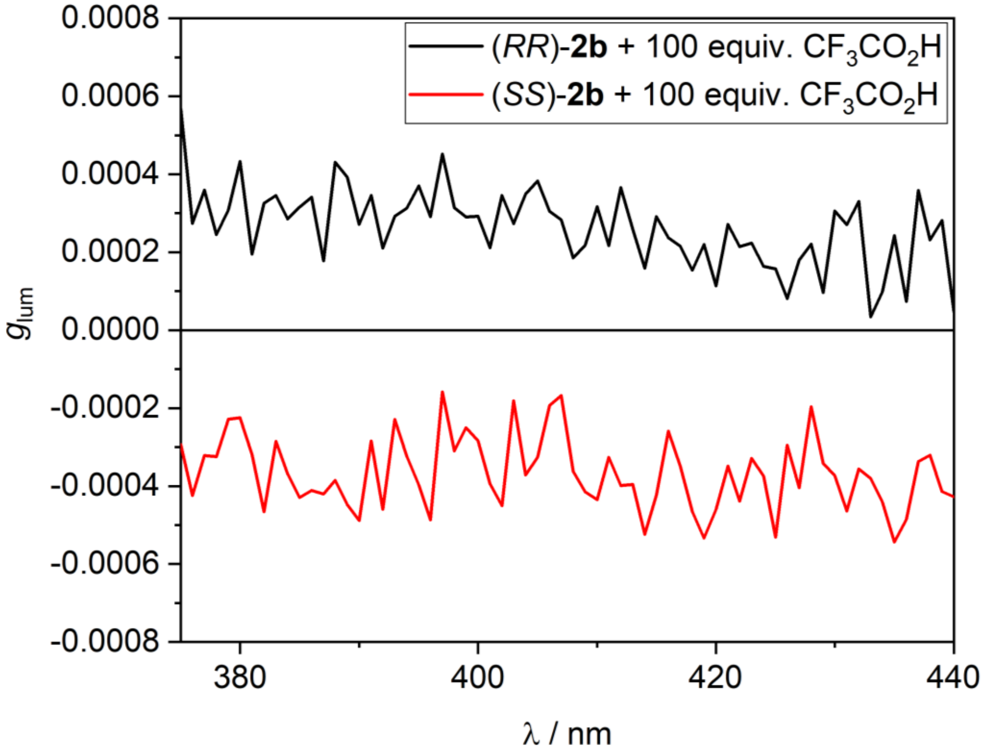

Figure S85. g_{lum} (λ_{exc} = 372 nm) spectra of (*RR*)/(*SS*)-**2b** in CH_2Cl_2 in the presence of 100 equiv. of CF_3CO_2H ($|g_{lum}| = 3 \times 10^{-4}$)

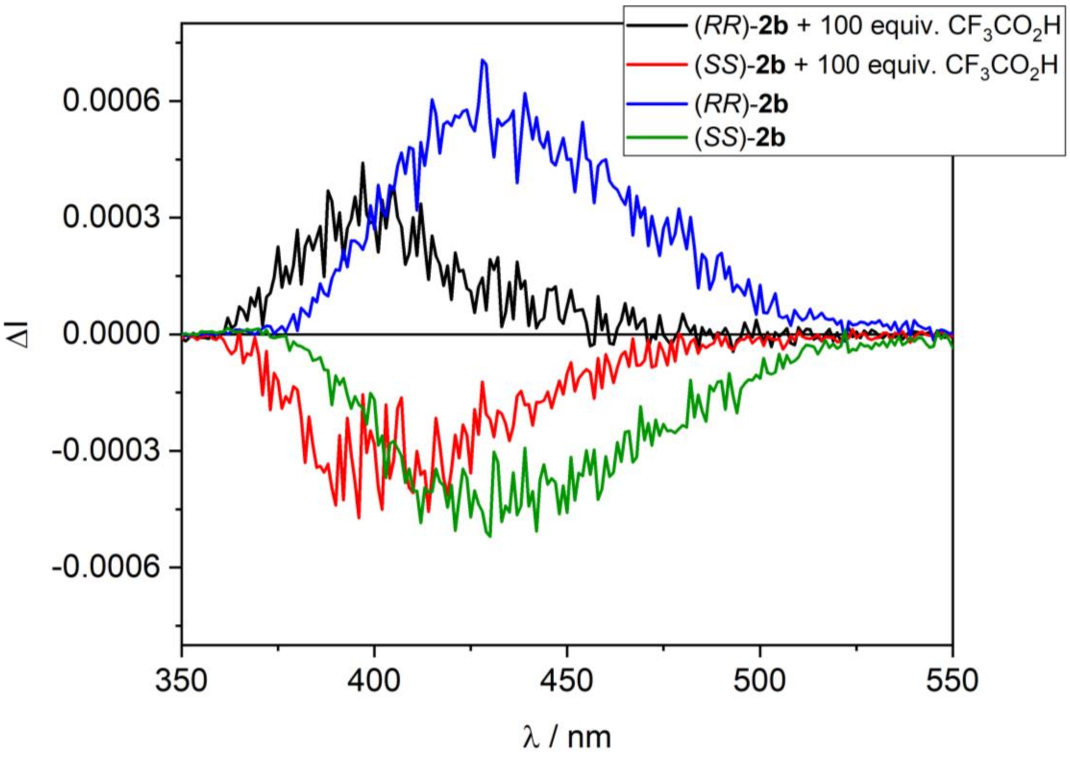

Figure S86. Comparison between the CPL (λ_{exc} = 372 nm) spectra of (*RR*)/(*SS*)-**2b** in CH_2Cl_2 in the presence (black and red lines) and the absence (blue and green lines) of 100 equiv. of CF_3CO_2H

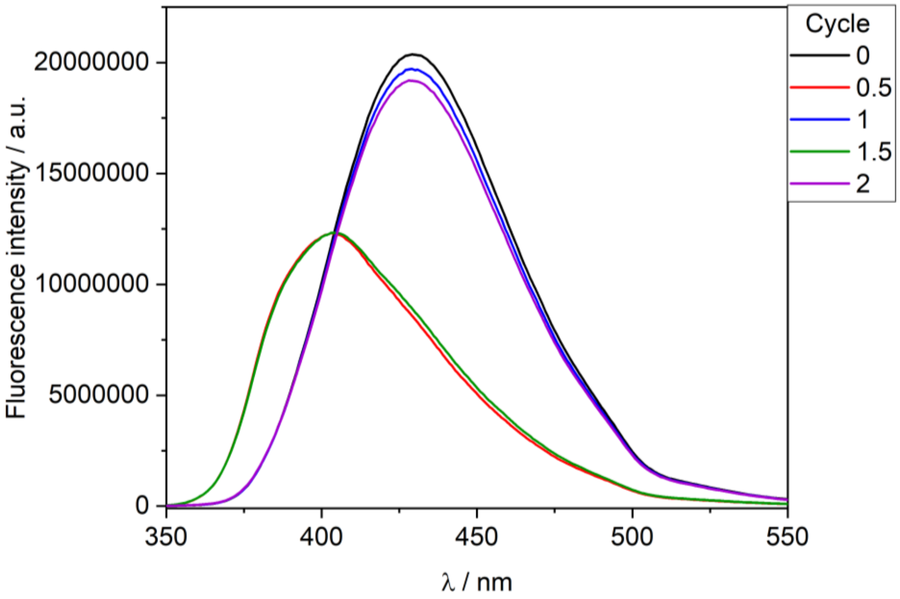

Figure S87. Switching of the fluorescence of compound **2b**: Emission (λ_{exc} = 372 nm) spectra of (*RR*)-**2b** after consecutive additions of CF_3CO_2H and Et_3N in CH_2Cl_2. Cycle 0 corresponds to the neutral solution of (*RR*)-**2b**, cycle 0.5 corresponds to the solution in cycle 0 after the addition of 100 equiv. of CF_3CO_2H, cycle 1 corresponds to the solution in cycle 0.5 after the addition of 110 equiv. of Et_3N, cycle 1.5 corresponds to the solution in cycle 1 after the addition of 140 equiv. of CF_3CO_2H, and cycle 2 corresponds to the solution in cycle 1.5 after the addition of 180 equiv. of Et_3N.

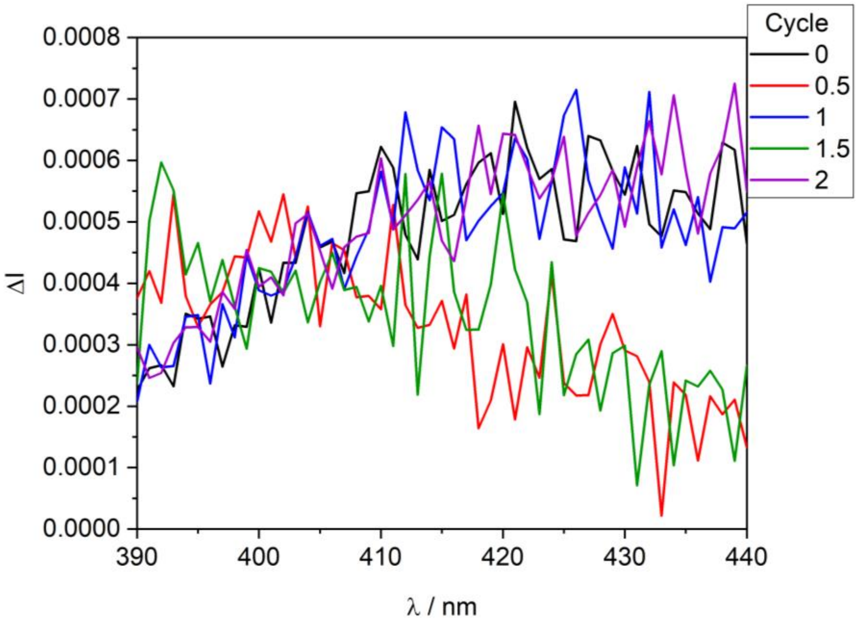

Figure S88. Switching of the CPL response of compound **2b**: CPL (λ_{exc} = 372 nm) spectra of (*RR*)-**2b** after consecutive additions of CF_3CO_2H and Et_3N in CH_2Cl_2. Cycle 0 corresponds to the neutral solution of (*RR*)-**2b**, cycle 0.5 corresponds to the solution in cycle 0 after the addition of 100 equiv. of CF_3CO_2H, cycle 1 corresponds to the solution in cycle 0.5 after the addition of 110 equiv. of Et_3N, cycle 1.5 corresponds to the solution in cycle 1 after the addition of 140 equiv. of CF_3CO_2H, and cycle 2 corresponds to the solution in cycle 1.5 after the addition of 180 equiv. of Et_3N.

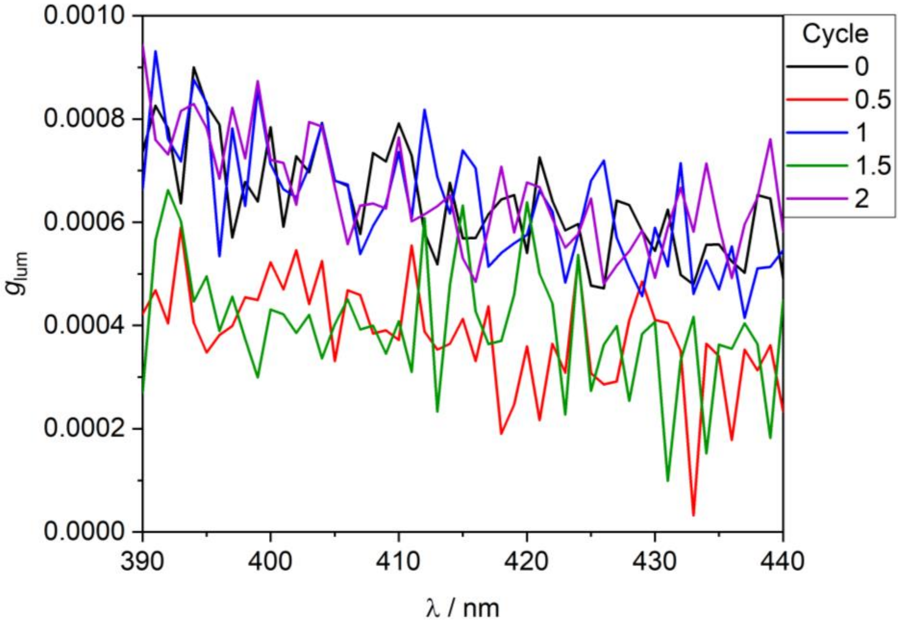

Figure S89. Switching of the CPL response of compound **2b**: g_{lum} (λ_{exc} = 372 nm) spectra of (*RR*)-**2b** after consecutive additions of CF_3CO_2H and Et_3N in CH_2Cl_2. Cycle 0 corresponds to the neutral solution of (*RR*)-**2b**, cycle 0.5 corresponds to the solution in cycle 0 after the addition of 100 equiv. of CF_3CO_2H, cycle 1 corresponds to the solution in cycle 0.5 after the addition of 110 equiv. of Et_3N, cycle 1.5 corresponds to the solution in cycle 1 after the addition of 140 equiv. of CF_3CO_2H, and cycle 2 corresponds to the solution in cycle 1.5 after the addition of 180 equiv. of Et_3N.

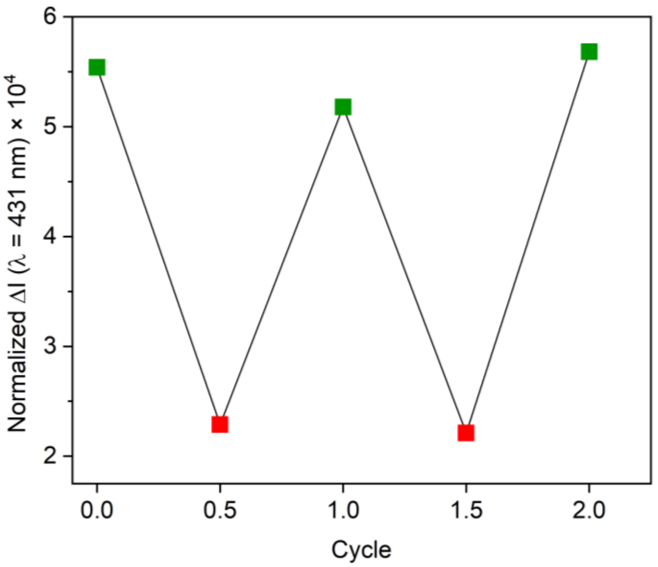

Figure S90. In situ switching of the CPL emission of (*RR*)-**2b** after consecutive addition of acid (CF_3CO_2H, red square) and base (Et_3N, green square). Cycle 0 corresponds to the neutral solution of (*RR*)-**2b**, cycle 0.5 corresponds to the solution in cycle 0 after the addition of 100 equiv. of CF_3CO_2H, cycle 1 corresponds to the solution in cycle 0.5 after the addition of 110 equiv. of Et_3N, cycle 1.5 corresponds to the solution in cycle 1 after the addition of 140 equiv. of CF_3CO_2H, and cycle 2 corresponds to the solution in cycle 1.5 after the addition of 180 equiv. of Et_3N.

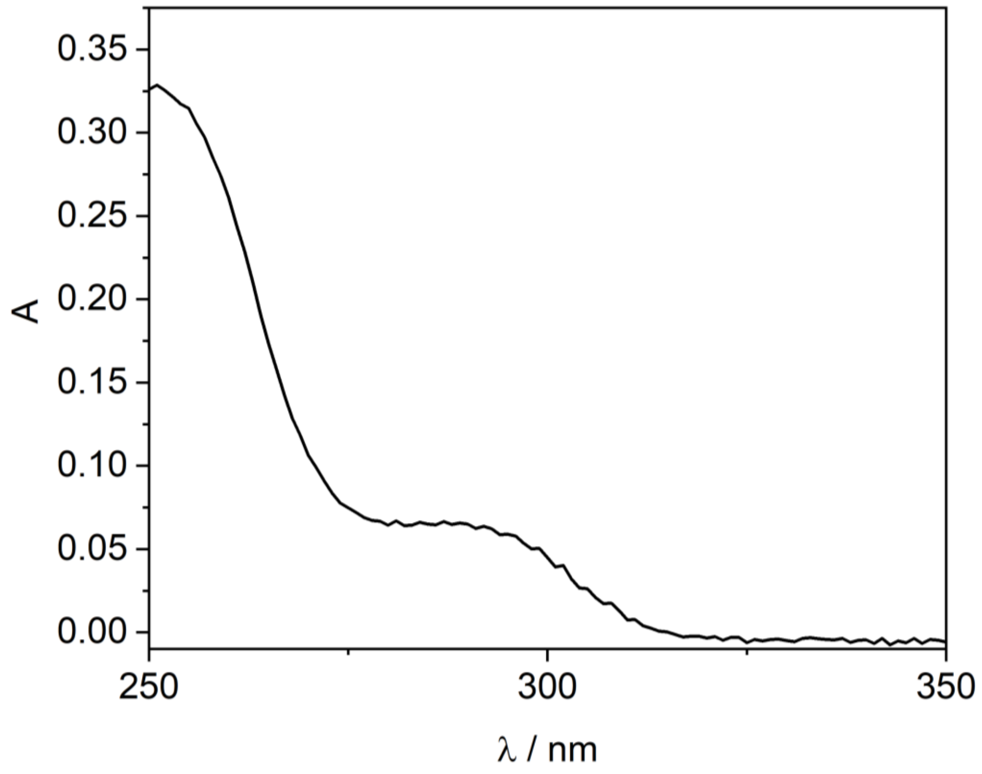

Figure S91. Absorption spectrum of (*RR*)-**5** in CH_2Cl_2

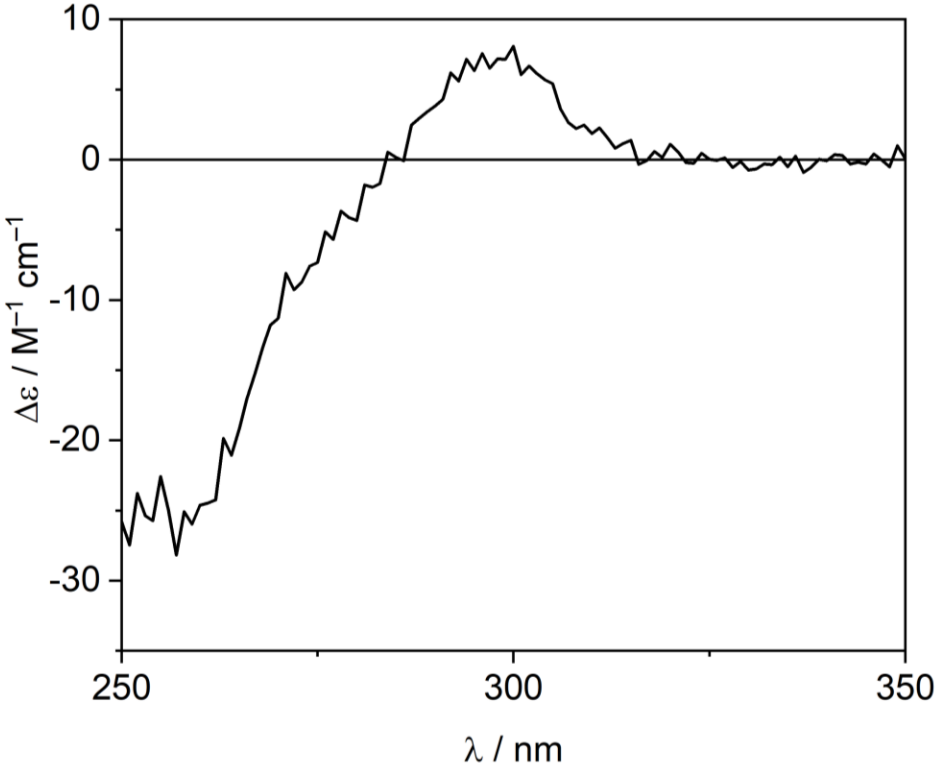

Figure S92. ECD spectrum of (*RR*)-**5** in CH_2Cl_2

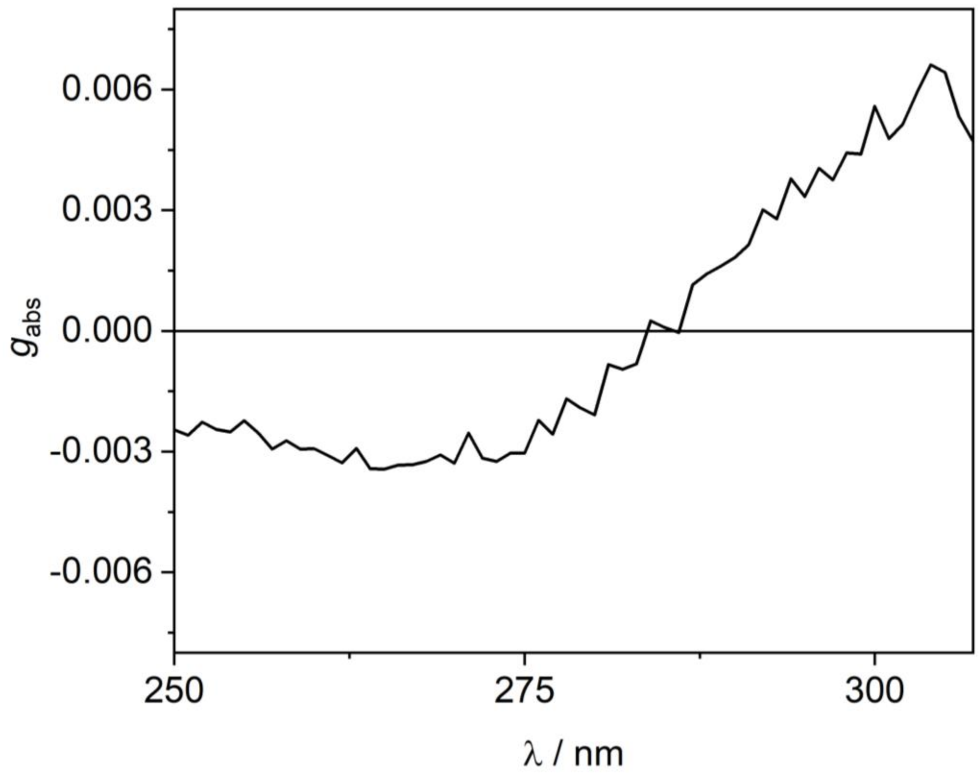

Figure S93. g_{abs} spectrum of (*RR*)-**5** in CH_2Cl_2

6. Computational Methods

DFT calculations

All the molecules were optimized and their optical properties (30 states) were computed at the CAM-B3LYP/def2tzvp level of theory, within an implicit solvation model for dichloromethane,[S7] using Gaussian09 software.[S8]

Molecular Dynamics simulations

Molecular dynamics simulations were done using GROMACS 2018.3 package.[S9] (*RR*)-**1a-3a** and (*SS*)-**1b-3b** and dichloromethane molecules were described through the generalized AMBER force field (GAFF).[S10] The conversion between AMBER and GROMACS formalisms was done using the acpype script.[S11] Atomic charges were computed following the parameterization procedure based on the HF/6-31G(d) RESP charges. The size of the cubic simulation box was defined considering a minimum distance of 3 nm from the edges of the molecule to the edges of the simulation box, ensuring a large layer of dichloromethane molecules around (*RR*)-**1a-3a** and (*SS*)-**1b-3b**. The electrostatic interactions were considered by applying periodic boundary conditions with a cutoff of 10 Å and the use of the Particle Mesh Ewald method.[S12] Different steps were then performed with (i) a minimization step using steepest descent method (ii) an equilibration step in the NVT ensemble for 1000 ps at T = 298 K, using the Berendsen weak coupling method[S13] (iii) an equilibration step in the NPT ensemble for another 1000 ps, at P = 1 bar, using the same Berendsen weak coupling method and finally (iv) a 20 ns production phase in the NPT ensemble with a time step of 2 fs, Nosé-Hoover thermostat,[S14] Parinello-Rahman barostat,[S15] with both $\tau = 1$ fs.

To average the optical and chiroptical properties of molecules **1a,b-3a,b**, 50 snapshots (structures) have been extracted from MD trajectories and their absorption and ECD spectra have been computed as described in the DFT calculations section. The resulting averaged spectrum obtained for each molecule is a simple average of those corresponding to the structures of the 50 snapshots.

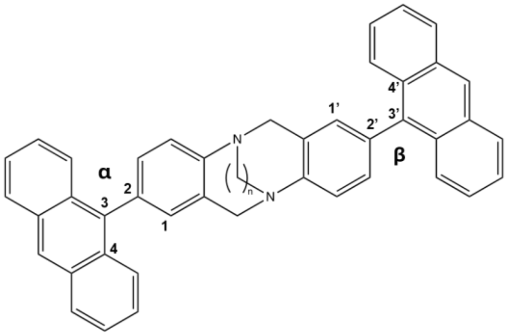

Figure S94. Definition of the two dihedral angles, α and β, illustrating the orientation of the lateral moieties with respect to the Tröger's base analogue core

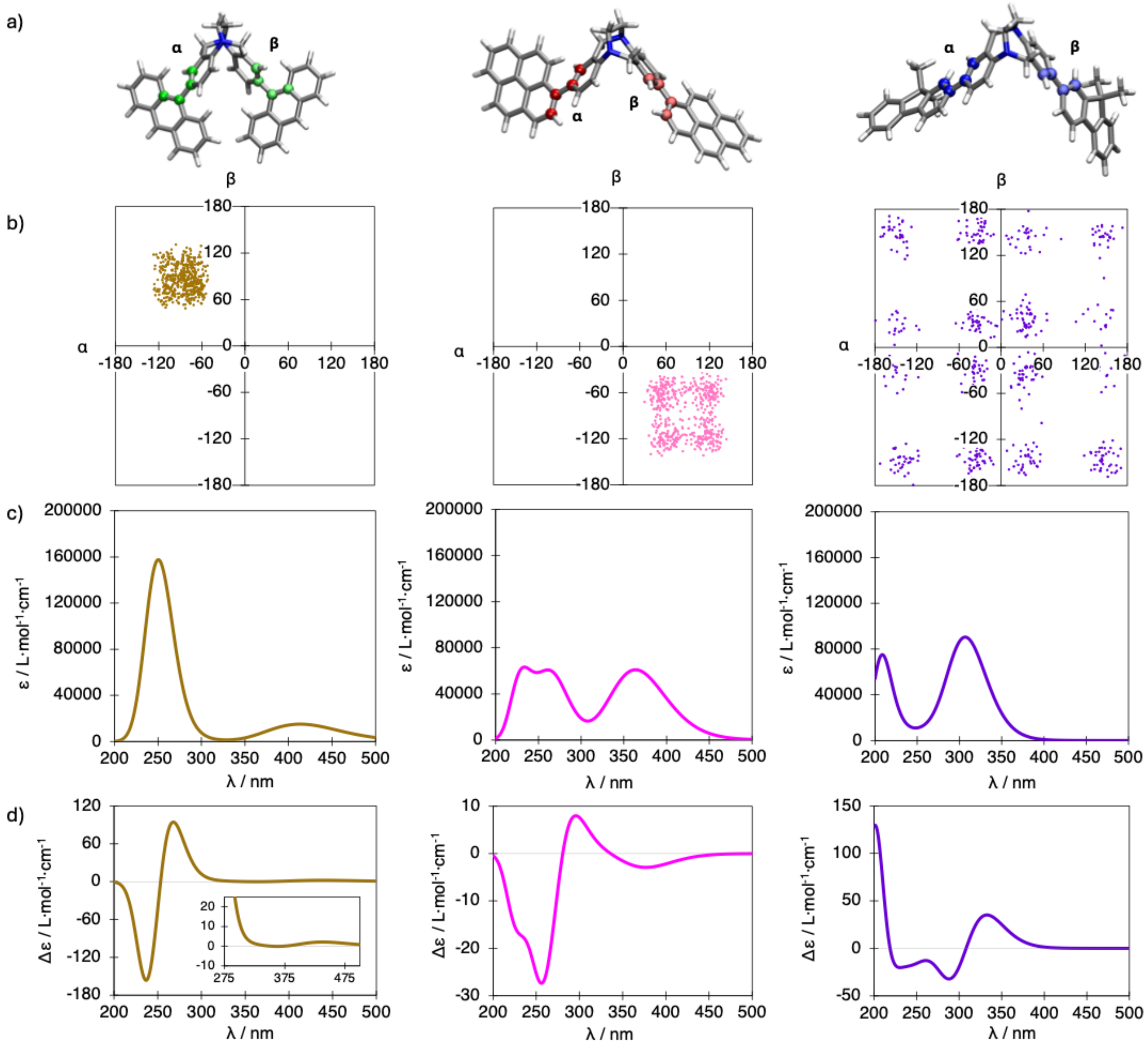

Figure S95. (a) Structures of ethano-bridged Tröger's base analogues (*SS*)-**1b-3b** showing the two dihedrals α and β. (b) Representation of the dihedral couple (α;β) of (*SS*)-**1b** (left), (*SS*)-**2b** (middle), and (*SS*)-**3b** (right) along the 20 ns MD simulation, where each point represents a snapshot of the simulation. For **1a,b-2a,b**, due to steric hindrance, only the half-rotation is possible and the dihedral angles extend over a 180° range (either [0;180] or [−180;0]). Note that for **3a,b**, since the complete rotation of the fluorene moieties is possible, the dihedral angles range from −180° to 180°. Averaged calculated (c) UV-Vis absorption and (d) ECD spectra of (*SS*)-**1b** (brown lines, left), (*SS*)-**2b** (pink lines, middle), and (*SS*)-**3b** (purple lines, right) for 50 extracted snapshots of MD simulation (full width at half maximum (FWHM) = 0.3 eV).

Table S17. Experimental absorption maxima (λ_{exp}, nm) and calculated (λ_{calc}, nm) absorption transitions of two representative structures of **1a** and **1b** extracted from MD simulations and optimized at DFT level. The oscillator strength (*f*), the attribution, the description, between parentheses, of the transition and the corresponding, after optimization, dihedral couple (α;β) are also given.

		λ_{exp}	λ_{calc}	*f*	Attribution (Description)	(α ; β)
**1a**	1	368	354<br>356	0.21<br>0.16	H−1 → L ($S_0 \rightarrow S_2$)<br>H → L ($S_0 \rightarrow S_1$)	(90 ; −80)
	2	368	354<br>357	0.21<br>0.17	H−1 → L ($S_0 \rightarrow S_2$)<br>H → L ($S_0 \rightarrow S_1$)	(90 ; −80)
**1b**	1	368	354<br>356	0.21<br>0.16	H−1 → L ($S_0 \rightarrow S_2$)<br>H → L ($S_0 \rightarrow S_1$)	(90 ; −80)
	2	368	354<br>357	0.21<br>0.17	H−1 → L ($S_0 \rightarrow S_2$)<br>H → L ($S_0 \rightarrow S_1$)	(90 ; −80)

Table S18. Experimental absorption maxima (λ_{exp}, nm) and calculated (λ_{calc}, nm) absorption transitions of four representative structures of **2a** and **2b** extracted from MD simulations and optimized at DFT level. The oscillator strength (*f*), the attribution, the description, between parentheses, of the transition and the corresponding, after optimization, dihedral couple (α;β) are also given.

		λ_{exp}	λ_{calc}	*f*	Attribution	(α ;β)
**2a**	1	280	255<br>259<br>264	0.24<br>0.63<br>0.24		(−120 ; 120)
		347	328	1.45	H−1/H → L/L+1 ($S_0 \rightarrow S_1$)	
	2	280	259<br>262	0.70<br>0.31		(−60 ; 120)
		347	328	1.61	H−1/H → L/L+1 ($S_0 \rightarrow S_1$)	
	3	280	254<br>259<br>264	0.30<br>0.60<br>0.27		(−120 ; 60)
		347	327	1.65	H−1/H → L/L+1 ($S_0 \rightarrow S_1$)	

	4	280	258	0.23		(−120 ; 120)
			264	1.24		
			266	0.27		
		347	337	1.80	H−1/H → L/L+1 ($S_0 \rightarrow S_1$)	
**2b**	1	280	253	0.84		(−60 ; 60)
			263	0.16		
			264	0.22		
		347	327	1.55	H−1/H → L/L+1 ($S_0 \rightarrow S_1$)	
	2	280	254	0.27		(−60 ; 120)
			263	0.28		
		347	328	1.27	H → L+1 ($S_0 \rightarrow S_1$)	
	3	280	253	0.64		(−60 ; 60)
			264	0.27		
		347	327	1.48	H → L ($S_0 \rightarrow S_1$)	
	4	280	253	0.54		(−120 ; 60)
			254	0.40		
			264	0.29		
		347	327	1.28	H → L ($S_0 \rightarrow S_1$)	

Table S19. Experimental absorption maxima (λ_{exp}, nm) and calculated (λ_{calc}, nm) absorption transitions of only four representative structures over the sixteen ones[a] of **3a** and **3b** extracted from MD simulations and optimized at DFT level. The oscillator strength (*f*), the attribution, the description, between parentheses, of the transition and the corresponding, after optimization, dihedral couple (α;β) are also given.

		λ_{exp}	λ_{calc}	f	Attribution	(α ;β)
**3a**	1	295	283	1.22	H−1 → L ($S_0 \rightarrow S_2$)	(40 ; 40)
		319	290	1.14	H → L+1 ($S_0 \rightarrow S_1$)	
	2	295	284	1.29	H−1 → L ($S_0 \rightarrow S_2$)	(−40 ; 40)
		319	290	1.03	H → L+1 ($S_0 \rightarrow S_1$)	
	3	295	283	1.30	H−1 → L+1 ($S_0 \rightarrow S_2$)	(40 ; −40)
		319	290	1.06	H → L+1 ($S_0 \rightarrow S_1$)	
	4	295	284	1.33	H−1 → L+1 ($S_0 \rightarrow S_2$)	(140 ; −40)
		319	291	1.00	H → L+1 ($S_0 \rightarrow S_1$)	
**3b**	1	295	283	1.12	H−1 → L ($S_0 \rightarrow S_2$)	(−140 ; −140)
		319	290	1.26	H → L+1 ($S_0 \rightarrow S_1$)	
	2	295	284	1.33	H−1 → L+1 ($S_0 \rightarrow S_2$)	(140 ; −40)
		319	290	1.01	H → L+1 ($S_0 \rightarrow S_1$)	
	3	295	283	1.22	H−1 → L ($S_0 \rightarrow S_2$)	(−140 ; 140)
		319	289	1.15	H → L+1 ($S_0 \rightarrow S_1$)	
	4	295	283	1.22	H → L+1 ($S_0 \rightarrow S_2$)	(140 ; −140)
		319	290	1.11	H−1 → L ($S_0 \rightarrow S_1$)	

[a] The choice has been made to sample only one quarter of the sixteen representative structures, with α and β ranging both between [0;180]. After the DFT optimization, the 4 structures span over the full dihedral angle range [-180;180]. We expect this behavior to happen if the three other quarters of the sixteen representative structures are sampled and thus consider this sample as sufficient in combination with MD sampling.

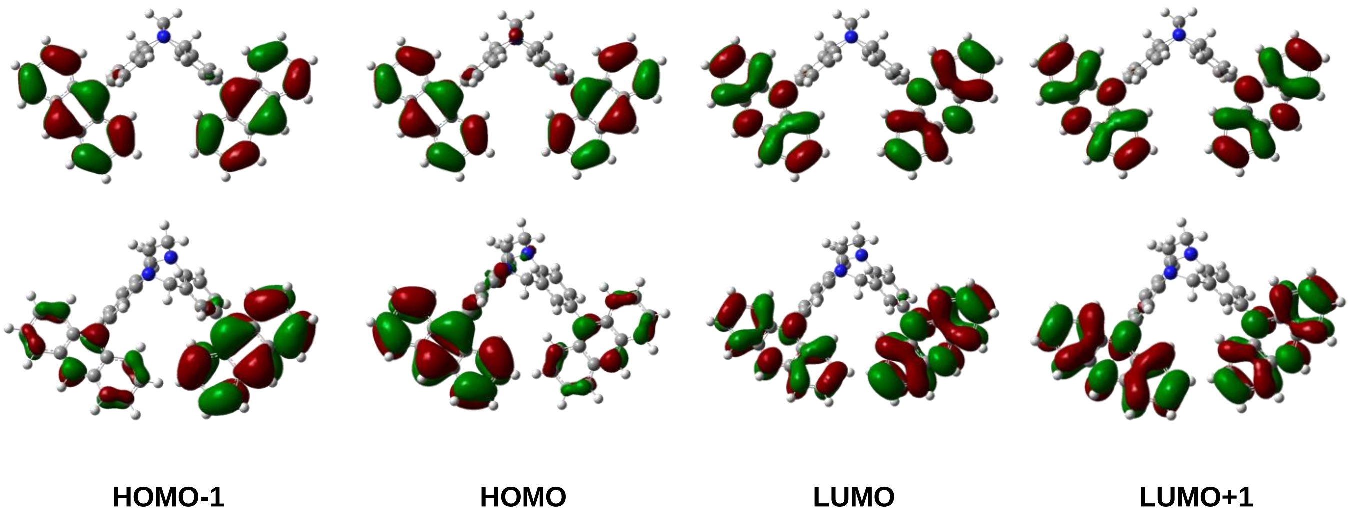

Figure S96. Representation of molecular orbitals involved in the main transition, λ_{max}, of (*RR*)-**1a** (top) and (*SS*)-**1b** (bottom). Isodensity=0.0004 a.u.

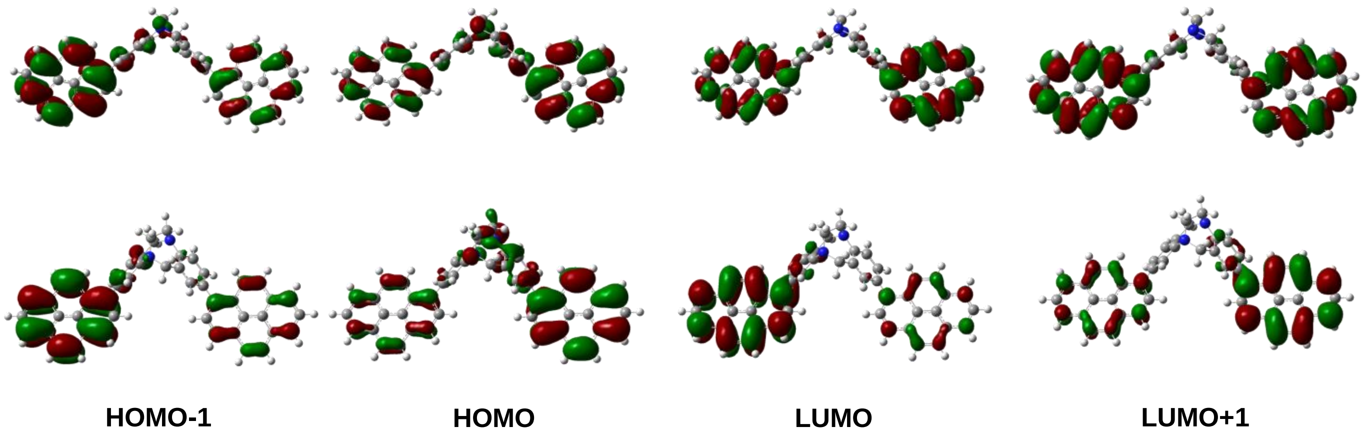

Figure S97. Representation of molecular orbitals involved in the main transition, λ_{max}, of **2a** (*RR*)-(top) and (*SS*)-**2b** (bottom). Isodensity=0.0004 a.u.

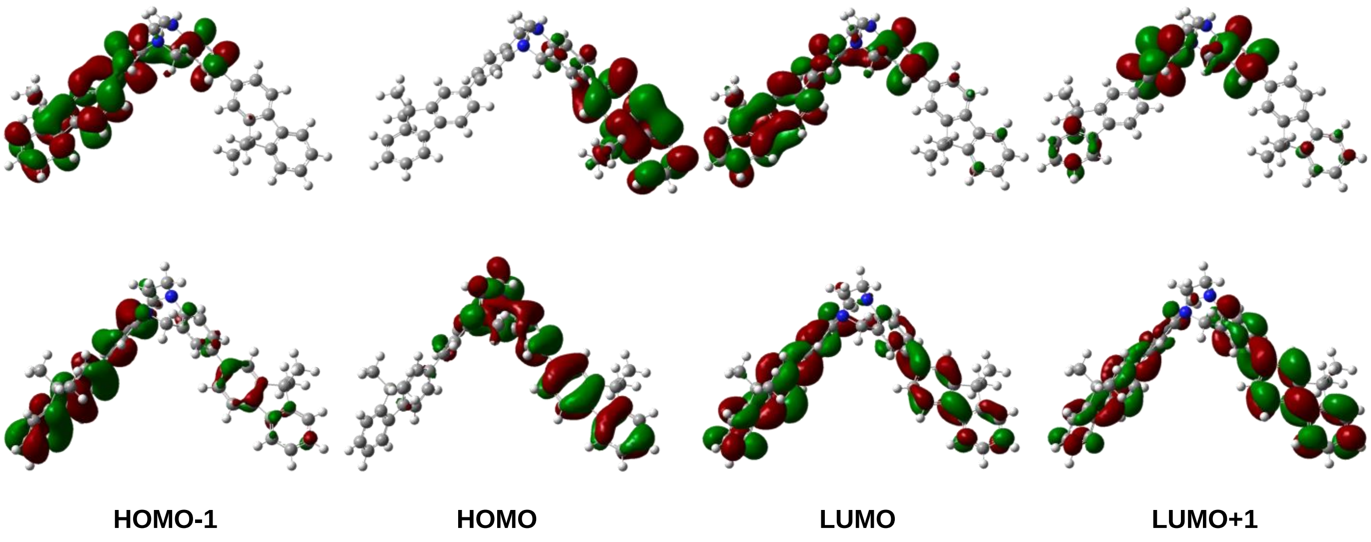

Figure S98. Representation of molecular orbitals involved in the main transition, λ_{max}, of (*RR*)-**3a** (top) and (*SS*)-**3b** (bottom). Isodensity=0.0004 a.u.

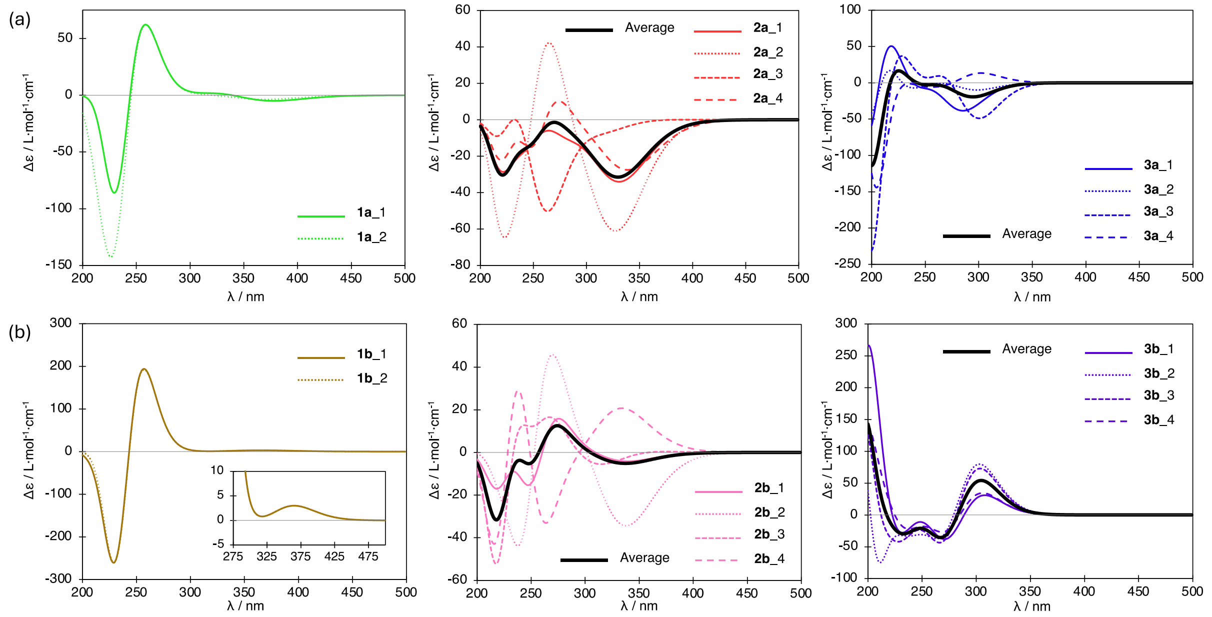

Figure S99. Calculated ECD spectra of (a) (*RR*)-**1a** (left), (*RR*)-**2a** (middle), and (*RR*)-**3a** (right) and (b) (*SS*)-**1b** (left), (*SS*)-**2b** (middle), and (*SS*)-**3b** (right) for the representative conformers of (*RR*)-**1a-3a** and (*SS*)-**1b-3b** (2 conformers for (*RR*)-**1a** and (*SS*)-**1b**, and 4 conformers for (*RR*)-**2a-3a** and (*SS*)-**2b-3b**). FWHM = 0.3 eV.

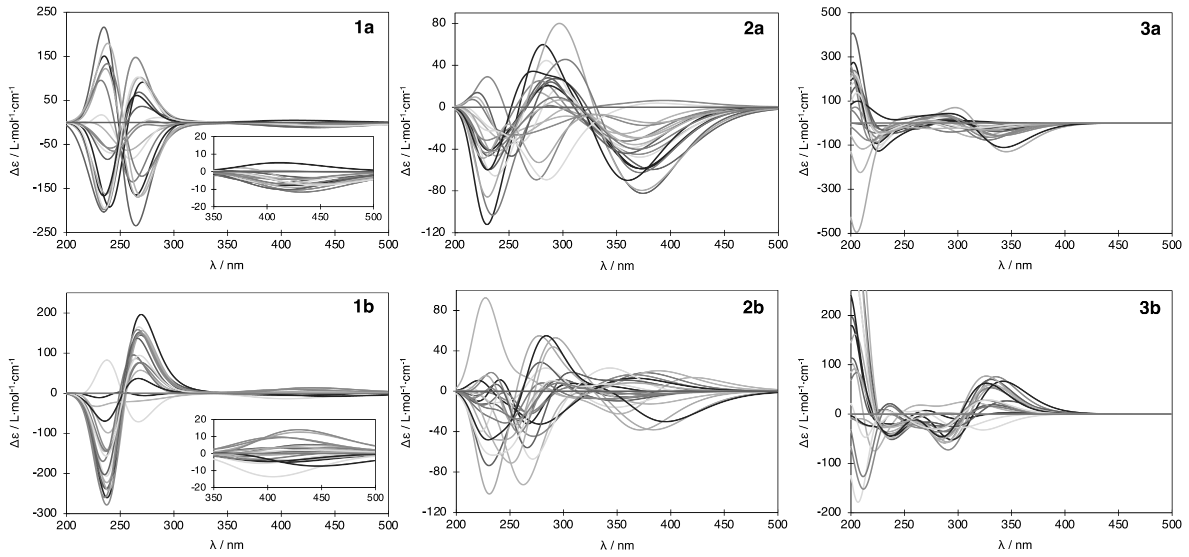

Figure S100. Calculated ECD spectra for (*RR*)-**1a-3a** and (*SS*)-**1b-3b** over 20 snapshots extracted from MD simulations. For the sake of clarity, only 20 snapshots, over the 50 snapshots extracted to obtain the average spectra, have been represented here to illustrate to variability of the calculated ECD spectra. FWHM = 0.3 eV.

7. References

[S1] J. Jensen, K. Wärnmark, *Synthesis* **2001**, *12*, 1873−1877.

[S2] D. L. Jameson, T. Field, M. R. Schmidt, A. K. DeStefano, C. J. Stiteler, V. J. Venditto, B. Krovic, C. M. Hoffman, M. T. Ondisco, M. E. Belowich, *J. Org. Chem.*, **2013**, 78, 11590-11596.

[S3] Y. Ishida, H. Ito, D. Mori, K. Saigo, *Tetrahedron Lett.* **2005**, *46*, 109–112.

[S4] J. Olmsted, *J. Phys. Chem.* **1979**, *83*, 2581–2584.

[S5] B. Valeur, M. N. Berberan-Santos, *Molecular Fluorescence. Principles and Applications, 2nd ed.*, Wiley-VCH, Weinheim, **2012**.

[S6] J. Lakowicz, *Principles of Fluorescence Spectroscopy, 3rd ed.*, Springer-Verlag, New York, **2006**.

[S7] J. Tomasi, B. Mennucci, R. Cammi, *Chem. Rev.* **2005**, *105*, 2999–3094.

[S8] M. J. Frisch, G. W. Trucks, H. B. Schlegel, G. E. Scuseria, M. A. Robb, J. R. Cheeseman, G. Scalmani, V. Barone, G. A. Petersson, H. Nakatsuji, X. Li, M. Caricato, A. V. Marenich, J. Bloino, B. G. Janesko, R. Gomperts, B. Mennucci, H. P. Hratchian, J. V. Ortiz, A. F. Izmaylov, J. L. Sonnenberg, D. Williams-Young, F. Ding, F. Lipparini, F. Egidi, J. Goings, B. Peng, A. Petrone, T. Henderson, D. Ranasinghe, V. G. Zakrzewski, J. Gao, N. Rega, G. Zheng, W. Liang, M. Hada, M. Ehara, K. Toyota, R. Fukuda, J. Hasegawa, M. Ishida, T. Nakajima, Y. Honda, O. Kitao, H. Nakai, T. Vreven, K. Throssell, J. A. Montgomery, Jr., J. E. Peralta, F. Ogliaro, M. J. Bearpark, J. J. Heyd, E. N. Brothers, K. N. Kudin, V. N. Staroverov, T. A. Keith, R. Kobayashi, J. Normand, K. Raghavachari, A. P. Rendell, J. C. Burant, S. S. Iyengar, J. Tomasi, M. Cossi, J. M. Millam, M. Klene, C. Adamo, R. Cammi, J. W. Ochterski, R. L. Martin, K. Morokuma, O. Farkas, J. B. Foresman, D. J. Fox, *Gaussian 09, Revision C.01*, Gaussian, Inc., Wallingford CT, **2016**.

[S9] M. J. Abraham, T. Murtola, R. Schulz, S. Páll, J. C. Smith, B. Hess, E. Lindahl, *SoftwareX* **2015**, *1–2*, 19–25.

[S10] J. Wang, R. M. Wolf, J. W. Caldwell, P. A. Kollman, D. A. Case, *J. Comput. Chem.* **2004**, *25*, 1157–1174.

[S11] (a) J. Wang, W. Wang, P. A. Kollman, D. A. Case, *J. Mol. Graph.* **2006**, *25*, 247–260. (b) P. R. Batista, A. Wilter, E. H. A. B. Durham, P. G. Pascutti, *Cell Biochem. Biophys.* **2006**, *44*, 395–404.

[S12] (a) T. Darden, D. York, L. Pedersen, *J. Chem. Phys.* **1993**, *98*, 10089–10092. (b) U. Essmann, L. Perera, M. L. Berkowitz, T. Darden, H. Lee, L. G. Pedersen, *J. Chem. Phys.* **1995**, *103*, 8577–8593.

[S13] H. J. C. Berendsen, J. P. M. Postma, W. F. van Gunsteren, A. DiNola, J. R. Haak, *J. Chem. Phys.* **1984**, *81*, 3684–3690.

[S14] (a) S. Nosé, M. Klein, *Mol. Phys.* **1983**, *50*, 1055–1076. (b) S. Nosé, *J. Chem. Phys.* **1984**, *81*, 511–519. (c) W. G. Hoover, *Phys. Rev. A* **1985**, *31*, 1695–1697.

[S15] M. Parrinello, A. Rahman, *J. Appl. Phys.* **1981**, *52*, 7182–7190.
